# Upregulation of Glycolytic Enzymes, Mitochondrial Dysfunction and Increased Cytotoxicity in Glial Cells Treated with Alzheimer’s Disease Plasma

**DOI:** 10.1371/journal.pone.0116092

**Published:** 2015-03-18

**Authors:** Tharusha Jayasena, Anne Poljak, Nady Braidy, George Smythe, Mark Raftery, Mark Hill, Henry Brodaty, Julian Trollor, Nicole Kochan, Perminder Sachdev

**Affiliations:** 1 Bioanalytical Mass Spectrometry Facility, MW Analytical Centre, University of New South Wales, Sydney, Australia; 2 Centre for Healthy Brain Ageing, School of Psychiatry, University of New South Wales, Sydney, Australia; 3 Neuropsychiatric Institute, the Prince of Wales Hospital, Sydney, Australia; 4 School of Medical Sciences, University of New South Wales, Sydney, Australia; 5 Dementia Collaborative Research Centre, University of New South Wales, Sydney, Australia; Temple University School of Medicine, UNITED STATES

## Abstract

Alzheimer’s disease (AD) is a neurodegenerative disorder associated with increased oxidative stress and neuroinflammation. Markers of increased protein, lipid and nucleic acid oxidation and reduced activities of antioxidant enzymes have been reported in AD plasma. Amyloid plaques in the AD brain elicit a range of reactive inflammatory responses including complement activation and acute phase reactions, which may also be reflected in plasma. Previous studies have shown that human AD plasma may be cytotoxic to cultured cells. We investigated the effect of pooled plasma (n = 20 each) from healthy controls, individuals with amnestic mild cognitive impairment (aMCI) and Alzheimer’s disease (AD) on cultured microglial cells. AD plasma and was found to significantly decrease cell viability and increase glycolytic flux in microglia compared to plasma from healthy controls. This effect was prevented by the heat inactivation of complement. Proteomic methods and isobaric tags (iTRAQ) found the expression level of complement and other acute phase proteins to be altered in MCI and AD plasma and an upregulation of key enzymes involved in the glycolysis pathway in cells exposed to AD plasma. Altered expression levels of acute phase reactants in AD plasma may alter the energy metabolism of glia.

## Introduction

Alzheimer’s disease (AD) is a neurodegenerative disorder that results in the progressive and irreversible loss of cholinergic neurons in specific areas of the brain [1]. Amnestic Mild Cognitive Impairment (aMCI) is considered to be a pre-dementia stage of AD [2], with a proportion of aMCI cases progressing to AD with time. AD is characterised by an abnormal accumulation of amyloid β (Aβ) and tau proteins, increased oxidative stress and redox metals in the brain all of which are associated with an immunological response [3]. Aβ primarily accumulates extracellularly and eventually leads to the formation of amyloid plaques, the main pathological hallmark of AD. The accumulation of Aβappears also appears to occur in synaptic mitochondria leading to impaired respiration and increased oxidative stress [4].

Damage to the blood-brain barrier is thought to occur in AD, and this may increase movement of proteins between the brain and the vasculature [5]. It is therefore possible that AD and its precursor, MCI, may be associated with the presence of specific biomarkers detectable in plasma and recent work has successfully used a panel of plasma biomarkers to predict disease severity and progression from MCI to dementia [6]. There are a number of proposed plasma biomarkers for AD, some of which reflect increased protein, lipid and nucleic acid oxidation and reduced activities of antioxidant enzymes in the AD brain [7–13]. AD has been reported to be associated with reduced plasma levels of vitamin A, C and E [9]. Isoprostanes, which arise from free-radical-mediated peroxidation of polyunsaturated fatty acids, are elevated in the AD brain, CSF and plasma [11]. 4-hydroxynonenal, another product of lipid peroxidation, is also increased in AD plasma [8].

A variety of inflammatory markers are increased with the onset of AD pathology, including cytokines and chemokines, coagulation factors and acute-phase reactive proteins as well as reactive astrocytes and activated microglial cells, the main cells involved in the neuroinflammation process [3,14]. Previous studies have shown that upregulation of the acute phase protein clusterin in plasma, is associated with prevalence, rate of progression, brain atrophy and disease severity in AD patients [15,16]. Other studies however have found no difference and suggest against the idea that acute phase protein changes in the CNS can be detected in plasma [17,18]. Alternatively, AD may be associated with a more widespread immune dysregulation, detectable in plasma.

Previous studies investigating the effects of human AD plasma on cells in culture have found differential effects on protein expression and cell biology. One study aimed to determine if exposure to serum from AD patients would affect markers for AD brain lesions [19], and found that 24 hour exposure to AD serum increased four molecular markers characteristic of AD senile plaques and neurofibrillary tangles (NFTs) in rat hippocampal neurons [19]. These markers were Alz-50, beta-amyloid, MAP2 and ubiquitin [19]. This stimulation of AD markers by human serum suggests that the genesis of both neuronal plaques and tangles may arise from exposure of susceptible neurons to toxic serum factors and/or failure to detoxify these factors. Another study found that antibodies in serum of patients with AD caused immunolysis of cholinergic nerve terminals from the rat cerebral cortex, supporting the hypothesis that autoimmune mechanisms may operate in the pathogenesis of AD [20].

Other studies have shown that serum of multiple sclerosis patients causes demyelination in rat CNS explant cultures and also induces cytotoxicity in rat oligodendrocytes in culture [21,22]. Demyelination was present not only in multiple sclerosis sera but was also found in sera from patients with other neurological diseases and complement was shown to be a factor involved in the effect [23,24]. In yet another study, human serum from patients with septic shock was shown to induce apoptosis in human cardiac myocytes [25]. This work demonstrated the utility of examining effects of disease plasma on cell culture systems, to facilitate the study of both disease markers and disease mechanisms.

Since previous studies indicate that AD plasma may contain oxidative stress markers as well as cytotoxic factors, we investigated the effect of the addition of pooled control, MCI and AD plasma from 20 individuals each on a microglial cell line. Cell viability, proliferation and mitochondrial function were investigated following 48 hour treatment with non heat-inactivated plasma and plasma in which complement proteins had been deactivated. We also tested the effect of commercially purchased complement factors alone and in combination on cultured glia. We then undertook proteomic analysis of the plasma from each group and iTRAQ quantitative proteomic analysis of cell extracts exposed to plasma from each group to investigate possible plasma protein alterations unique to MCI or AD, to detect any protein aberrations within the cells treated with the plasma and to correlate these finding to cell viability and mitochondrial function assays measured *in vitro*.

## Materials and Methods

### Subjects

Age matched healthy control (n = 20), amnestic mild cognitive impairment (aMCI, n = 20), and probable AD (n = 20) plasmas were pooled and used in both the cell culture and plasma proteomics experiments. AD patients were recruited from the Memory Clinic of the Department of Old Age Psychiatry of the Prince of Wales Hospital and participants in a clinical drug trial of donepezil (Aricept). All met the NINCDS-ADRDA criteria [26] for probable AD. The aMCI subjects were recruited from the Memory and Ageing Study, a longitudinal study of community dwelling individuals aged 70–90 [27]. The diagnosis of aMCI was determined using the Petersen Criteria as follows [28]: (i) subjective complaint of memory impairment, (ii) objective impairment in memory (performance >1.5 SD below normal for age on a standardised memory test) (iii) essentially preserved general cognitive function (MMSE ≥ 24) (iv) intact functional activities as indicated by instrumental activities of daily living; and (v) not meeting DSM-IV criteria for dementia. Healthy control subjects had a normal performance for age on a range of neuropsychological tests and intact functional activities. Ethics committee approval was obtained from the University of New South Wales (UNSW) and the South Eastern Sydney Illawarra Area Health Service (SESIAHS) ethics committees and written informed consent was obtained from all participants.

### Cell Culture

CHME-5 cells are a human microglial cell line obtained from embryonic fetal human microglia through transformation with SV-40 virus [29,30] and were a generous gift from Prof Gilles Guillemin (Macquarie University, Sydney, Australia). These cells express antigens present on adult human microglia, secrete pro-inflammatory cytokines upon activation, exhibit properties of primary human microglia and have been successfully used as a model of microglial activation by others [29,31]. Cells were maintained in RPMI1640 cell culture medium, supplemented with 10% heat inactivated foetal bovine serum, 2 mM l-glutamine, and 1% penicillin/streptomycin, at 37°C in a humidified atmosphere containing 95% air/5% CO_2_. Before experimentation, cells were seeded into 24 or 96 well culture plates to a density of approximately 1x10^4^ or 2x10^3^ cells respectively. Cells were left overnight and then supplemented with up to 20% (by volume) heat-inactivated and non heat-inactivated control, MCI or AD plasma for 48 hours. For the cell viability and iTRAQ proteomic analyses cells were washed to remove all plasma, and lysed in RIPA buffer (Thermo Fisher Scientific, IL, USA) followed by sonication. For the complement factor experiments cells were seeded into plates and treated with 1, 5 or 10 μg of each complement factor or the complement standard (in a total volume of 200μL cell culture media) and then incubated for 48 hours. These concentrations are within the physiological range of these proteins in plasma [32].

### MTT Cell Proliferation Assay

In actively proliferating cells, an increase in 3-[4,5-dimethylthiazol-2-yl]-2,5-diphenyl tetrazolium bromide (MTT) conversion in cells relative to controls represents an increase in cellular proliferative activity. Conversely, in cells that are undergoing apoptosis, MTT conversion, and thus biological activity, decreases. Cell proliferation was analysed using established protocols [33,34].

### NAD(H) Assay

Damaged cells show mitochondrial dysfunction, which results in decreased cellular nicotinamide adenine dinucleotide (NAD) levels. Intracellular NAD(H) concentration was quantified using the thiazolyl blue microcycling assay established by Bernofsky and Swan and adapted here for the 24 well plate format [35].

### Lactate Dehydrogenase (LDH) Leakage

Cytoplasmic enzyme leakage has been shown to be a useful tool for measuring early cellular damage or impairment [12], and has also been used as a sign of cytotoxicity [36,37]. LDH is released from cells due to loss of membrane integrity. Therefore LDH was measured in the cell culture medium as well as in cell homogenates as another measure of cell viability.

### XF24 Microplate-Based Respirometry as a measure of mitochondrial function

To determine the effect of human control, MCI and AD plasma on oxygen consumption rates (OCRs; an indicator of mitochondrial respiration) and extracellular acidification rates (ECARs; a measure of glycolytic flux) in the microglial cell line, the Seahorse XF24, extracellular flux analyzer (Seahorse Bioscience, North Billerica, MA, USA) was employed and assays performed as previously described [38–40]. The basal control ratio (BCR) and the uncoupling ratio (UCR) were determined as previously described [41]. Essentially, the BCR is a measure of how close the basal level of respiration is to the maximum level of respiration. The closer this ratio is to 1, the greater the mitochondrial malfunction. The UCR is a measurement of mitochondrial functional integrity and measures the ratio of uncoupled to physiologically normal respiration levels. The greater the maximum level of respiration, the greater the mitochondrial functional integrity.

### Fractionation of Plasma

Control, MCI and AD plasma from 20 subjects was pooled and fractionated by two methods. To fractionate it into its protein and metabolite fractions, a PD10 column separation method was used. The PD10 column was washed with MilliQ water before the addition of 500 μL of plasma. The flow-through was collected as 750 μL fractions topping the column with MilliQ water. In total 20 fractions were collected and the absorbances read at 280nm. For the proteomics analysis, fractionation into low and high abundant protein fractions was undertaken using an MARS-Hu6 column (Agilent Technologies, CA, USA) according to the manufacturer’s instructions. The MARS-Hu6 column depletes the top 6 contaminating proteins (albumin, IgG, IgA, transferrin, haptoglobin and antitrypsin) from plasma. This eliminates the masking effect of highly abundant proteins so lower-abundant proteins can be more easily detected. The low abundance fraction produced was used for the proteomic analysis experiments.

### Proteomics of MCI and AD plasma

A one dimensional SDS 4–12% NuPAGE (Thermo Fisher Scientific Inc, MA, USA) gel was run using the low abundance fraction from each of the three groups. The gel was colloidal coomassie stained [42], and the lanes uniformly cut into 7–8 bits using a gridcutter and mount from The Gel Company, CA, USA. The gel bits were trypsin digested and then analysed using mass spectrometry as outlined in detail in S1 Methods.

Peak lists were generated by MassLynx (version 4.0 SP1, Micromass) using the Mass Measure program and submitted to the database search program Mascot (version 2.2, Matrix Science, London, England). Search parameters were: Precursor and product ion tolerances ± 0.25 and 0.2 Da respectively; Met(O) and Cys-carboxyamidomethyl were specified as variable modification, enzyme specificity was trypsin, one missed cleavage was possible on the NCBInr database.

Scaffold Q+ (version 4.3.4), Proteome Software, Portland, OR, USA) was used to identify any altered proteins between the groups. The Scaffold programme uses mass spectrometric data to identify protein changes in biological samples by collating Mascot data and using the ProteinProphet algorithm [43]. We compared the normalised total spectral count values from Scaffold [44] with the emPAI values from Mascot [45], which uses a different algorithm for spectral counting.

### iTRAQ Proteomics of Cell Lysates Treated with Human Control, MCI and AD Plasma

Two biological replicates of cells treated with 10% (by volume) non-heat inactivated fetal bovine serum, human control, MCI or AD plasma were washed to remove all plasma/serum and then lysed using RIPA buffer and probe sonicated. Total protein concentrations were determined using the Bicinchoninic acid (BCA) protein assay kit (Pierce, IL, USA). The total protein (120 μg) from each sample was reduced with 2 μl of 5mM tris-(2-carboxyethyl) phosphine (TCEP) for 60 min at 60°C followed by alkylation with 200 mM iodoacetamide (1 μL) for 10 min at room temperature. To remove any buffer components incompatible with mass spectrometry a buffer exchange was performed with 50 mM NaHCO_3_ using Microcon centrifugal filter devices with a 3,000 Da nominal molecular weight limit membrane (Millipore, MA, USA) to give a final protein concentration of 1 μg/μl.

For tryptic digestion 100 μg total protein from each sample was incubated overnight at 37°C with 4 ug of trypsin. Samples were labelled using the 8-plex iTRAQ kit (Applied Biosystems, CA, USA). Each iTRAQ reporter label was mixed with a biological replicate of cell lysate sample, pH adjusted to basic (ca pH = 9) with 2 μl of 50 mM of Na_2_CO_3_ and incubated for 2 hours. The reporter masses for the samples were labelled as follows: fetal bovine serum; 113 and 117, human control plasma; 114 and 118, human MCI plasma; 115 and 119, human AD plasma; 116 and 121.

Sample clean-up was performed using a strong cation exchange cartridge (Applied Biosystems, CA, USA) and a syringe pump at a flow rate of 9.5 ml/hr, and using the manufacturer’s protocol. Sample was then passed through a C18 Peptide Macrotrap (Michrom Bioresources, CA, USA). The flow-through from the C18 step was passed through an Oasis cartridge (Waters, MA, USA) to maximise peptide recovery and the two eluants pooled and dried under vacuum, resuspended in 0.2% heptafluorobutyric acid and then analysed using mass spectrometry as outlined in detail in S1 Methods.

Protein identification and quantification was performed using the MS/MS data (WIFF files) and the Paragon algorithm as implemented in Protein Pilot v4.0 software (Applied Biosystems, CA, USA) using the NCBInr database. Only proteins identified with a ProteinPilot unused score of ≥ 1.3 (greater than 95% confidence in sequence identity) were accepted as previously described [46,47]. The unused score is a ProteinPilot generated value for the level of confidence in protein sequence identification. As an approximate guide, ProteinPilot unused scores give the following percentage levels of confidence; score ≥1.3(≥95% confidence), score ≥2(≥99% confidence), score ≥3 (≥99.9% confidence) [46]. The only fixed modification used was iodoacetamide alkylation of cysteine residues. Mass tolerances were set to 50ppm for the precursor and 0.2 Da for the fragment ions. Autobias correction was applied to correct for any systematic bias in total protein concentration during sample pooling. Both biological replicates for the three human plasma types (control, MCI and AD) were compared to the fetal bovine serum control (iTRAQ reporter 117) and data exported to Microsoft Excel software (Microscoft, WA, USA).

Protein interactions between dysregulated proteins were determined using the web-based bioinformatics tool STRING v9.1 (http://string-db.org). STRING has a database that collates information on protein-protein interactions and associations. It scores and weights connections and provides predicted interaction network maps from literature mining searches. The 27 proteins which were significantly deregulated in glia treated with AD plasma, but not deregulated in either control or MCI plasma treated glia were analysed in STRING v9.1. MCL clustering was used with the 2 clusters option picked and with the confidence view selected to display the strength of evidence for protein associations and analysis of enrichment was also performed.

### Statistics

All cell viability values are presented as means ± SEM. Statistical comparisons were performed using two-tailed student *t*-tests assuming equal variance. Differences between treatment groups were considered statistically significant at the p < 0.05 level. Scaffold values are represented as normalised total spectral counts and p-values for significantly altered proteins were obtained using the ProteinProphet algorithm of the Scaffold Q+ software (Proteome Software, OR, USA). All iTRAQ values are presented as ratios of cells treated with human plasma to cells treated with fetal bovine serum control. Ratios and p-values for significantly altered proteins were obtained through the Paragon algorithm of the Protein Pilot v4.0 software (Applied Biosystems, CA, USA).

## Results

### Cell Proliferation

Cells treated with non heat-inactivated pooled control and AD plasma for 48 hours showed a significant decrease in cell proliferation in cells treated with AD plasma compared to controls (Table 1). The addition of MCI plasma to the cells also caused a similar drop in cell proliferation, though not reaching statistical significance (Table 1). Mild heat treatment at 56°C for 30 minutes is an established approach for inactivating complement proteins [48,49]. Such heat treatment prevented the effects of MCI and AD plasma on cell proliferation (Table 1).

**Table 1:** Cell viability as measured by MTT absorbance (abs) at 570nm, LDH release and intracellular NAD levels of microglial cells after 48 hour incubation in pooled, non heat inactivated and heat inactivated MCI and AD plasma.

		Non Heat Inactivated Plasma									Heat Inactivated Plasma					
		Control	Control	Control	MCI	MCI	MCI	AD	AD	AD	Control	Control	MCI	MCI	AD	AD
		MTT abs	NAD (ng)	LDH Activity (U/L)	MTT abs	NAD (ng)	LDH Activity (U/L)	MTT abs	NAD (ng)	LDH Activity (U/L)	MTT abs	NAD (ng)	MTT abs	NAD (ng)	MTT abs	NAD (ng)
**5% plasma**	**Mean**	0.21	10.71		0.23	10.84		0.14	10.3							
	**SEM**	0.05	3.84		0.05	1.26		0.03*	1.82							
**10% plasma**	**Mean**	0.13	3.61	**Media** 3.35 **Lysate** 11.4	0.09	2.35	**Media** 2.7 **Lysate** 13.06	0.04	0.89	**Media** 6.09 **Lysate** 7.76	0.13		0.11		0.11	
	**SEM**	0.03	1.25	**Media** 0.36 **Lysate** 1.19	0.02	0.85	**Media** 0.3 **Lysate** 1.22	0.01**	1.3*	**Media** 1.05* **Lysate** 1.0	0.03		0.04		0.02	
**20% plasma**	**Mean**	0.06	0.79		0.06	0.56		0.03	0			14.02		12.3		12.1
	**SEM**	0.01	0.14		0.004	0.07		0.004**	0**			1.15		0.60		0.65

* p ≤ 0.05 vs Control,

** p ≤ 0.01 vs Control

Cell viability was determined by measurement of cell proliferation, intracellular NAD levels and LDH activity in cell culture media and cell lysate homogenates. n = 9 (nine replicates) for cell proliferation measurements, n = 6 (six replicates) for NAD concentration and LDH activity. Plasma used for the measurements were obtained from the pooled plasma of 20 patients from each of the three groups (Control, MCI and AD) investigated. Three concentration levels of plasma were tested: 5%, 10% and 20% plasma as a percentage of total media volume.

**Fig 1:**
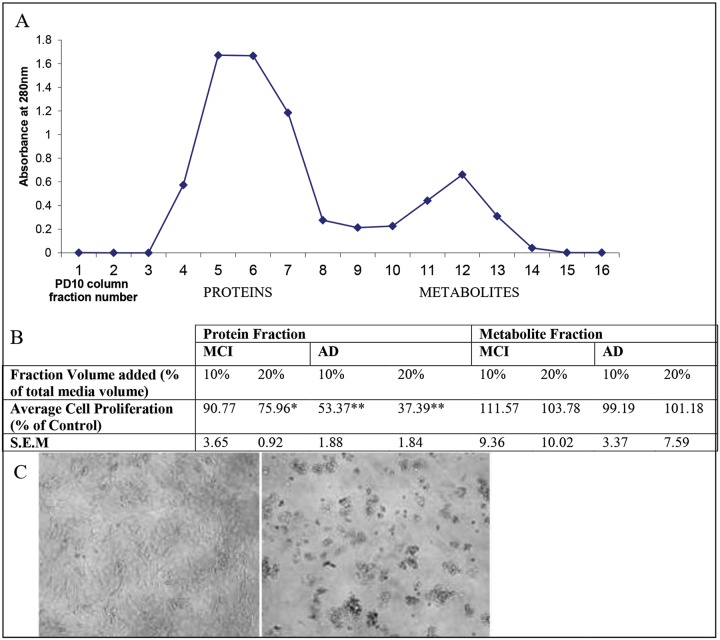
Fractionation of non heat inactivated control plasma into protein and metabolite fractions and the effects of plasma treatment on cell proliferation. Panel A: Fractionation of non heat inactivated control plasma into protein and metabolite fractions using PD10 column Panel B: Effect of these fractions on cell proliferation. Three replicates were performed. Plasma used for the measurements were obtained from the pooled plasma of 20 patients from each of the three groups (Control, MCI and AD). * p ≤ 0.01 vs Control, ** p ≤ 0.001 vs Control. Panel C: Images of microglia after 48 hour incubation with non heat inactivated 20% control plasma (left) and 20% AD plasma (right), showing increased toxicity and reduced cell proliferation in the AD plasma treated cells.

To determine whether the effect on cell proliferation was exclusively due to the protein component or to both the protein and low molecular weight components of plasma we separated proteins and metabolites. Plasma was fractionated using a PD10 column into its protein and metabolite portions to determine which portion of the plasma was causing the cytotoxic effect (Fig. 1). Addition of these two fractions to the cells showed that it was exclusively the protein portion which was initiating the reduction in cell proliferation (Fig. 1). Protein fractions of both MCI and AD plasma were found to significantly reduce cell proliferation (Fig. 1). The metabolite fraction of the plasma had no significant effect on microglial proliferation (Fig. 1).

Treatment of the cells with complement factors C1q, C1 inhibitor, C4, C5 and C9 effected a downward trend in cell proliferation with increasing concentration, but did not reach significance (Table 2). In combination, the factors were found to reduce cell proliferation at the highest concentration tested. The human complement standard which contains the factors C1q, C2, C3, C4, C5, C6, C7, C8, C9 and factor B was found to be the most potent at preventing cell proliferation (Table 2).

**Table 2:** Cell viability of microglial cells after 48 hour incubation with human complement components (C1q, C1 inhibitor, C4, C5 and C9), both individually and in combination with each other; and a human complement standard containing complement components C1q, C2, C3, C4, C5, C6, C7, C8, C9 and factor B.

	Cell Proliferation (abs at 570nm)	Complement C1q	Complement C1 inhibitor	Complement C4	Complement C5	Complement C9	C1q + C4	C5 + C9	C1q, C1inhib, C4, C5 + C9	Complement Standard	Complement Standard NAD concentration (ng)
Control (0μg/μl)	Average	0.317	0.609	0.454	0.375	0.324	0.384	0.356	0.378	0.511	0.114
	SEM	0.019	0.07	0.025	0.031	0.014	0.025	0.018	0.013	0.004	0.003
0.005μg/μl	Average	0.3	0.593	0.458	0.382	0.333	0.377	0.371	0.377	0.426	0.104
	SEM	0.042	0.02	0.019	0.028	0.0098	0.019	0.025	0.019	0.031	0.010
0.025μg/μl	Average	0.31	0.571	0.461	0.364	0.336	0.366	0.359	0.331*	0.210**	0.094
	SEM	0.018	0.03	0.02	0.025	0.0096	0.025	0.021	0.015	0.036	0.063
0.05μg/μl	Average	0.267	0.49	0.435	0.355	0.344	0.289*	0.265*	0.292*	0.129**	0.063*
	SEM	0.0075	0.05	0.019	0.03	0.021	0.027	0.013	0.024	0.018	0.0024

Cell viability was determined by MTT assay of cell proliferation and intracellular NAD levels (for complement standard samples). Replicates included n = 6 for cell proliferation measurements and, n = 3 for NAD concentrations.

* p ≤ 0.05 vs Control,

** p ≤ 0.01 vs Control

### NAD levels and LDH Leakage

Incubation with non heat inactivated plasma caused a significant drop in cell viability as reflected in lower NAD levels, for cells treated with both MCI and AD plasma, compared to controls (Table 1). This result was again prevented by plasma heat inactivation (Table 1). The addition of human complement standard containing C1q, C2, C3, C4, C5, C6, C7, C8, C9 and factor B was found to significantly reduce NAD levels in the microglia (Table 2).

A significant increase in LDH leakage into the cell culture medium was seen in cells incubated with non heat inactivated AD plasma (Table 1). A concurrent decrease was seen in the amount of intracellular LDH in these same cells (Table 1).

### Mitochondrial Function and Cellular Bioenergetics

**Fig 2:**
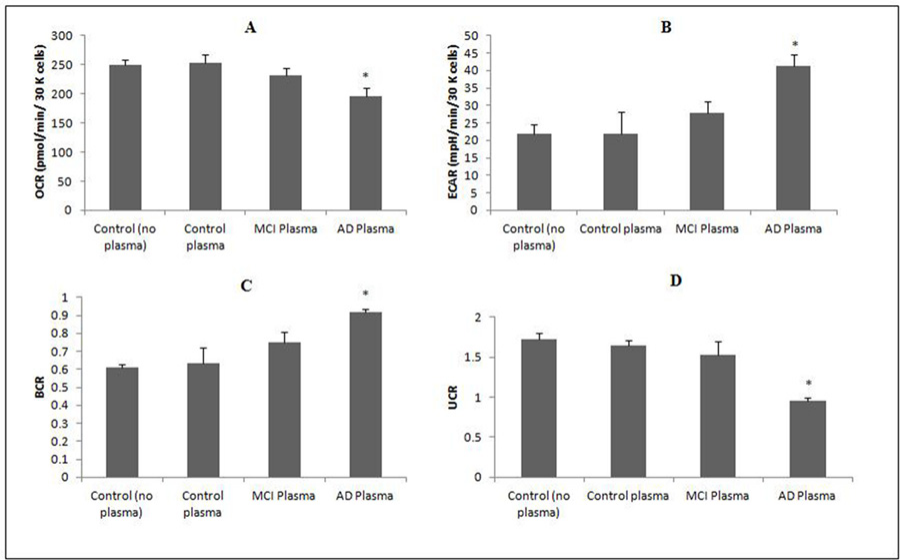
Effects of human plasma on cellular bioenergetics in a microglial cell line. **(A)** Effect of human plasma on oxygen consumption rates (OCR) in a microglial cell line for 48 hours. *p<0.05 compared to non-treated cells (control); (n = 4 for each treatment group). **(B)** Effect of human plasma on extracellular acidification rates (ECAR) in a microglial cell line for 48 hours. *p<0.05 compared to non-treated cells (control); (n = 4 for each treatment group). **(C)** Effect of human plasma on the basal control ratio (BCR) in a microglial cell line for 48 hours. *p<0.05 compared to non-treated cells (control); (n = 4 for each treatment group). **(D)** Effect of human plasma on the uncoupling ratio (UCR) in a microglial cell line for 48 hours. *p<0.05 compared to non-treated cells (control); (n = 4 for each treatment group).

To determine whether mitochondrial bioenergetic mechanisms are associated with AD pathogenesis, we assessed mitochondrial function in glial cells treated with human plasma using the Seahorse XF24 (Seahorse Bioscience, MA, USA). We observed a significant decrease in OCRs and an increase in ECAR for cells treated with AD plasma and MCI plasma effected similar trends though did not achieve statistical significance (Fig. 2). We also observed a significant increase in the BCR and a decline in the UCR in microglial cells after 48 hour incubation with AD plasma (Fig. 2), consistent with impaired mitochondrial function and increased shift towards glycolysis.

### Plasma fractionation and 1D gel electrophoresis

**Fig 3:**
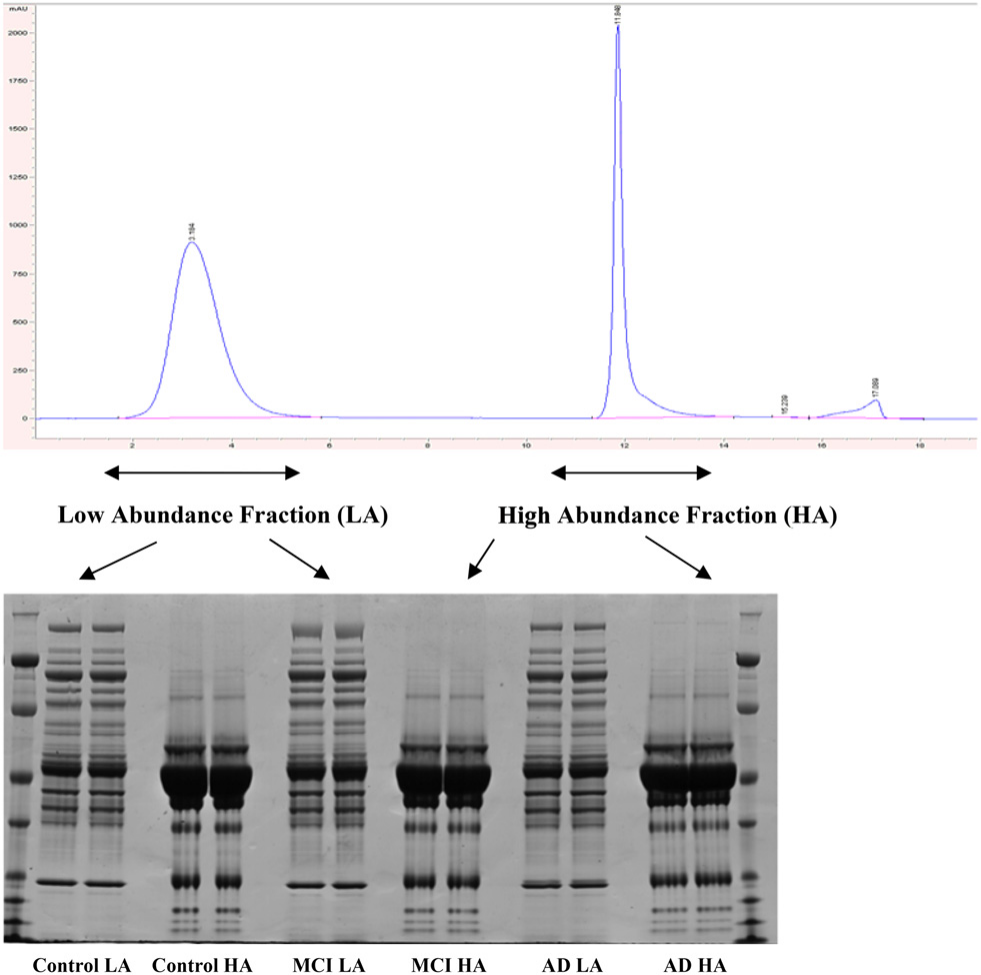
Chromatogram of fractionation using Hu6 column and 1D SDS/PAGE of these fractions. Low abundant proteins are eluted first (first peak on chromatogram) and high abundant proteins are eluted after (second peak). Gel shows significant depletion of high abundant proteins in the low abundant fractions. Loading was 50 μg/lane. First and last lanes contained molecular weight markers. Each fraction was run in duplicate.

Fractionation using the MARS-Hu6 column provided a baseline separation of low and high abundant proteins (Fig. 3). These fractions were run on a 1D SDS NuPage gel and proteins were shown to be effectively separated with a substantial depletion of high abundant proteins, revealing many lower abundant protein bands in the low abundant fraction (Fig. 3).

### Proteomics of MCI and AD plasma

Following proteomic analysis of pooled control, MCI and AD plasma, normalised total spectral counts using Scaffold software and emPAI values from Mascot showed complement component 2, fibronectin and fibrinogen to be significantly increased in the AD groups compared to the control group (Table 3). Thrombin was decreased in the MCI and AD patients compared to controls (Table 3). A full list of proteins identified and normalised spectral counts in human control, MCI and AD plasma can be found in S1 Table and the peptide false discovery rate analysis can be found in S1 Fig.

**Table 3:** Average normalised spectral counts (obtained from Scaffold) and emPAI values (obtained from Mascot) of significantly deregulated proteins identified in pooled plasma samples between the Control, MCI and AD groups.

		Complement component 2 (gi|14550407)			Complement component 1 inhibitor (gi|114642584)			Complement component 4 binding protein (gi|4502503)			Fibronectin (gi|109658664)			Thrombin (gi|119588383)			Fibrinogen (gi|70906435)			Alpha-1B-glycoprotein (gi|46577680)		
		C	MCI	AD	C	MCI	AD	C	MCI	AD	C	MCI	AD	C	MCI	AD	C	MCI	AD	C	MCI	AD
**Normalised Spectral Count**	**Average**	0	0.82	2.77	9.98	7.45	11.30	4.18	3.38	4.51	18.9	15.1	32.2	10.34	5.57	6.29	39.78	35.93	43.19	6.08	8.32	7.64
	**S.E.M**	0	0.50	0.29	1.0	0.46	2.10	0.51	0.46	0.83	0.18	2.55	2.29	1.18	0.50	0.88	5.58	2.42	7.25	0.49	0.70	1.01
	**p-value**		0.015#	0.000074**		p = 0.06 vs AD						0.0025##	0.0012**		0.098*	0.033*					0.040*	
**emPAI value**	**Average**	0.08	0.06	0.145	0.74	0.62	0.95	0.35	0.18	0.26	0.39	0.30	0.64	0.83	0.46	0.48	5.40	4.74	7.87	0.78	0.86	0.75
	**S.E.M**	0.010	0.020	0.014	0.090	0.06	0.13	0.027	0.037	0.029	0.068	0.065	0.033	0.068	0.055	0.033	0.48	0.62	0.71	0.10	0.032	0.029
	**p-value**		0.014#	0.025*		p = 0.06 vs AD				0.013*		0.0071##	0.0018**		0.0057*	0.0034*		0.016#	0.036*		0.050#	

Values are averages from 4 replicates. Plasma used was obtained from the pooled plasma of 20 patients from each of the three groups (Control, MCI and AD) with depletion of high abundance proteins.

* p ≤ 0.05 vs Control

** p ≤ 0.01 vs Control

# p ≤ 0.05 vs AD

## p ≤ 0.01 vs AD

### iTRAQ proteomic analysis of cell lysates treated with Control, MCI and AD plasma

**Fig 4:**
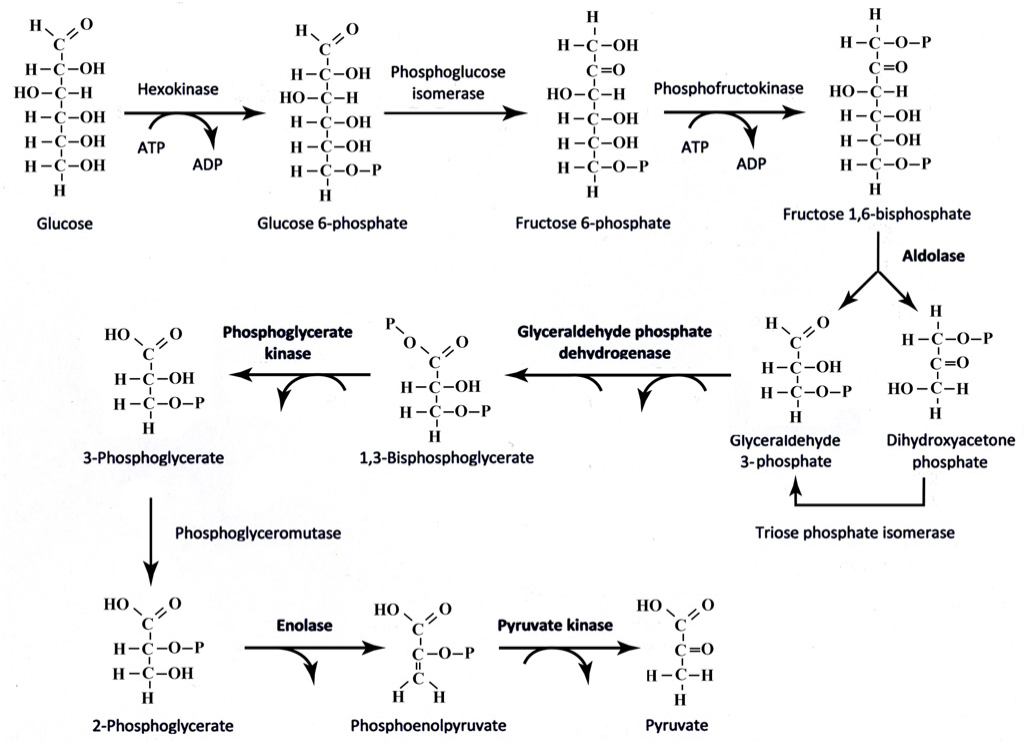
Glycolysis Pathway highlighting enzymes which were shown to be upregulated in cells treated with AD plasma.

**Fig 5:**
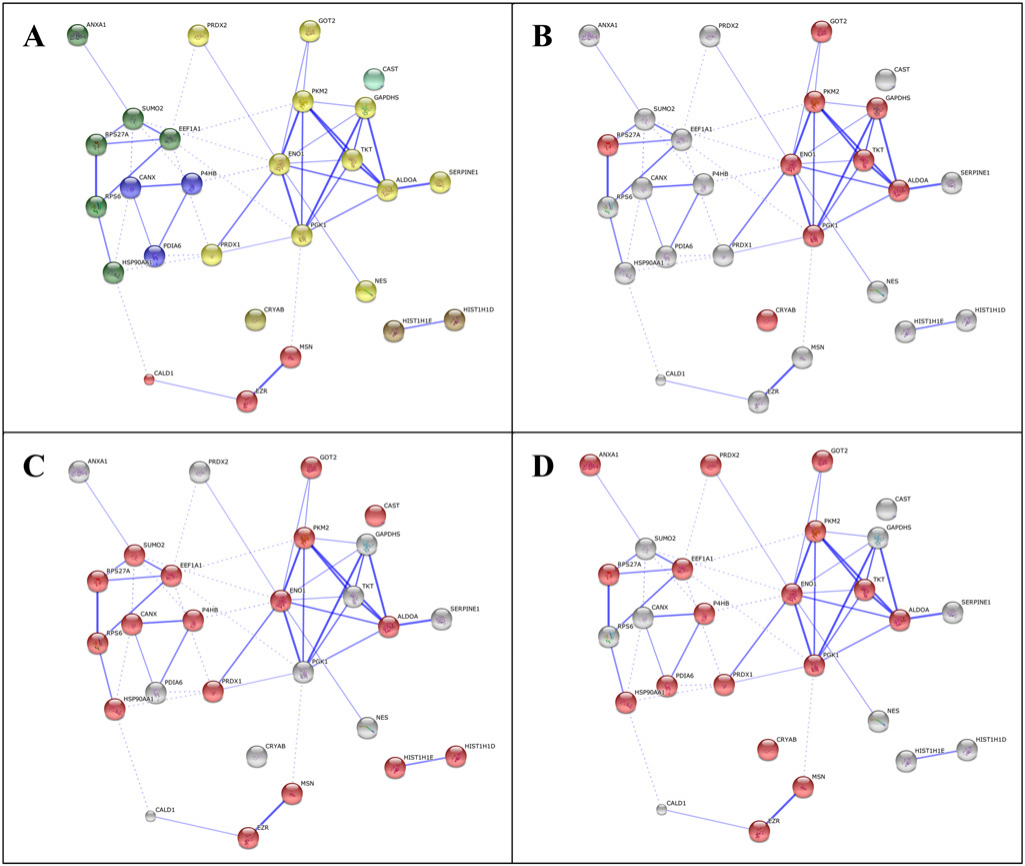
The 27 proteins which were significantly deregulated in glia treated with AD plasma, but not deregulated in either control or MCI plasma treated glia (shown in Table 4) were analysed in STRING v9.1. MCL clustering was used with the 2 clusters option picked and with the confidence view selected to display the strength of evidence for protein associations (panel A). Analysis of enrichment was also performed and the most significantly enriched biological process was glucose metabolic process (FDR p-value = 1.759x10E-8, with the 9 proteins involved in this process shown in panel B). Other distinct biological processes which were also significantly enriched included response to hydrogen peroxide (FDR p-value = 3.559x10E-2 with 4 proteins involved; ANXA1, PRDX1, PRDX2, CRYAB) and membrane to membrane docking (FDR p-value = 4.299x10E-2 with 2 proteins involved; MSN, EZR). Several molecular functions were also enriched, the most significant being RNA binding (FDR p-value = 5.340x10E-9 with 17 proteins involved shown in panel C). Another distinct and significantly enriched molecular function is thioredoxin peroxidase activity (FDR p-value = 9.220x10E-3 with 4 proteins involved; PRDX1, PRDX2). Several cellular components were also enriched, the most significant of these being extracellular vesicle exosome (FDR p-value = 5.019x10E-9 with 17 proteins involved as shown in panel D). Multiple other significantly enriched cellular components were also observed, and all enriched protein groups are shown in Table 5.

Differential protein expression in glial cells treated with fetal bovine serum (control) or human plasma from control, MCI and AD subjects were analysed with two biological replicates performed using an 8-plex iTRAQ experimental design. In total, 791 proteins were identified with 95% or greater confidence in correct protein sequence identification and 750 proteins with a false discovery rate of 5% (see S2 Table for full summary of identified proteins and S3 Table for full false discovery rate analysis). Fourty-one proteins were found altered between the MCI and AD groups of cellular lysates (Table 4). The highest numbers of dysregulated proteins were found in the cells treated with AD plasma (Table 4, 27 proteins highlighted in bold). Interestingly a significant number of proteins involved in the glycolysis cycle were shown to be upregulated in this group, namely glyceraldehyde-3-phosphate dehydrogenase, phosphoglycerate kinase, enolase, aldolase and pyruvate kinase. These enzymes catalyse five of the ten enzyme reactions of the pathway and their functions are shown in Fig. 4. Transketolase was also shown to be significantly elevated in both the AD plasma treated cell sample replicates. This enzyme is part of the pentose phosphate pathway and connects this pathway to glycolysis. Analysis of protein interactions of the 27 dysregulated glial proteins treated with AD plasma using the online STRING v9.1 tool confirmed a significant enrichment of proteins involved in glucose metabolism (Fig. 5 and Table 5).

**Table 4:** Dysregulated proteins in glial cells treated with human control, MCI and AD plasma compared to FBS (non human serum control) following iTRAQ analysis.

			Replicate 1		Replicate 2		Replicate 1		Replicate 2		Replicate 1		Replicate 2	
Protein Function	Accession #	Name	Ctrl:FBS	PVal	Ctrl:FBS	PVal	MCI:FBS	PVal	MCI:FBS	PVal	AD:FBS	PVal	AD:FBS	PVal
**Glycolysis**	gi|7669492	**glyceraldehyde-3-phosphate dehydrogenase**	0.97	0.524	1.02	0.603	0.98	0.676	0.98	0.655	**1.27**	3.4E-5*	**1.32**	1.0E-4*
	gi|4505763	**phosphoglycerate kinase 1**	1.07	0.175	1.05	0.322	1.05	0.279	1.06	0.207	**1.2**	7.0E-5*	**1.31**	1.1E-4*
	gi|4503571	**alpha-enolase isoform 1**	0.93	0.12	0.98	0.753	0.99	0.719	0.98	0.553	**1.19**	2.7E-4*	**1.22**	1.0E-4*
	gi|342187211	**fructose-bisphosphate aldolase A**	0.99	0.829	1	0.989	1.02	0.836	1	0.991	**1.2**	0.004*	**1.22**	0.001*
	gi|33286418	**pyruvate kinase isozymes M1/M2**	0.98	0.746	1.02	0.726	1.01	0.869	1.03	0.6	**1.22**	0.008*	**1.22**	0.002*
	gi|4507521	**transketolase isoform 1**	0.98	0.686	1.01	0.881	1.04	0.323	0.99	0.749	**1.13**	0.022*	**1.16**	0.002*
**Chaperone**	gi|20070125	**protein disulfide-isomerase precursor**	0.98	0.566	0.96	0.352	0.93	0.057	1.01	0.821	**0.93**	0.026*	0.98	0.478
	gi|153792590	**heat shock protein HSP 90-alpha**	1.03	0.679	1.05	0.601	1.06	0.512	1.06	0.473	**1.3**	0.036*	1.2	0.059
	gi|66933005	**calnexin precursor**	1	0.942	0.98	0.7	0.94	0.315	0.93	0.149	0.92	0.266	**0.88**	0.010*
	gi|5031973	**protein disulfide-isomerase A6 precursor**	0.97	0.429	0.98	0.662	0.94	0.134	1	0.952	0.96	0.546	**0.91**	0.019*
	gi|21361657	protein disulfide-isomerase A3 precursor	0.96	0.427	0.98	0.653	**0.9**	0.013*	1	0.871	0.92	0.117	0.89	0.06
	gi|16507237	78 kDa glucose-regulated protein precursor	0.99	0.806	0.97	0.729	**0.92**	0.038*	0.98	0.643	0.91	0.101	0.91	0.108
**Cytoskeletal**	gi|4505257	**moesin**	1.08	0.265	1.14	0.243	1.12	0.106	1.03	0.721	**1.22**	0.021*	**1.2**	0.028*
	gi|21614499	**ezrin**	1.06	0.498	0.95	0.718	1.05	0.555	1.13	0.446	**1.31**	0.033*	0.9	0.712
	gi|38176300	**nestin**	0.99	0.773	1	0.984	0.97	0.518	1.04	0.354	0.94	0.173	**0.85**	0.004*
	gi|44680105	**caldesmon isoform 1**	0.91	0.154	0.97	0.773	0.9	0.174	0.94	0.478	0.87	0.085	**0.86**	0.027*
	gi|19920317	cytoskeleton-associated protein 4	**0.85**	0.049*	**0.77**	0.012*	0.92	0.229	**0.79**	0.032*	**0.74**	1.0E-4*	**0.77**	0.019*
	gi|62414289	vimentin	0.97	0.23	1	0.965	**0.9**	2.4E-4*	0.99	0.778	0.97	0.261	0.96	0.277
**Proteolysis**	gi|4506713	**ubiquitin-40S ribosomal protein S27a**	0.98	0.738	0.82	0.159	1.06	0.286	1.04	0.594	0.84	0.066	**0.77**	0.022*
	gi|66346681	**plasminogen activator inhibitor 1**	1.04	0.587	0.99	0.922	1	0.999	0.93	0.476	0.97	0.612	**0.79**	0.023*
	gi|54792069	**small ubiquitin-related modifier 2**	1.15	0.291	1.02	0.948	1.46	0.083	1.41	0.052	1.23	0.26	**1.29**	0.043*
	gi|109637759	**calpastatin isoform f**	0.86	0.228	0.75	0.138	0.81	0.24	0.88	0.46	0.82	0.206	**0.74**	0.045*
**Translation**	gi|4503471	**elongation factor 1-alpha 1**	0.97	0.493	1.02	0.76	0.93	0.225	0.97	0.563	**1.13**	0.024*	**1.24**	0.014*
	gi|17158044	**40S ribosomal protein S6**	0.95	0.62	0.54	0.063	0.83	0.091	0.7	0.172	**0.74**	0.041*	0.72	0.178
	gi|15431288	60S ribosomal protein L10a	1.14	0.206	**1.31**	0.037*	1.19	0.098	**1.27**	0.048*	1.41	0.079	1.06	0.732
	gi|214010226	40S ribosomal protein S24 isoform d	0.68	0.062	**0.66**	0.015*	**0.75**	0.011*	0.66	0.085	0.6	0.063	**0.64**	0.022*
	gi|124494254	proliferation-associated protein 2G4	0.97	0.897	0.99	0.974	**1.36**	0.040*	1.22	0.194	1.24	0.276	1.15	0.146
**Transcription**	gi|4885379	**histone H1.4**	0.86	0.158	0.78	0.053	0.79	0.063	0.96	0.61	**0.58**	0.006*	0.79	0.349
	gi|4885377	**histone H1.3**	1.04	0.538	1.21	0.386	0.94	0.438	1.18	0.369	**0.75**	0.012*	0.96	0.613
	gi|4885381	histone H1.5	**0.83**	0.011*	0.77	0.057	**0.79**	0.006*	0.9	0.164	**0.65**	3.4E-4*	0.85	0.205
	gi|4885375	histone H1.2	0.87	0.059	**0.73**	0.002*	**0.74**	0.002*	0.95	0.468	**0.54**	0.001*	0.76	0.102
**Immune response**	gi|4502101	**annexin A1**	1	0.999	0.99	0.848	0.93	0.161	1.02	0.794	**0.87**	0.023*	**0.83**	0.042*
	gi|50845388	annexin A2 isoform 1	1.04	0.339	**1.1**	0.021*	**0.89**	8.0E-4*	0.99	0.647	0.95	0.192	0.93	0.1
	gi|48255891	glucosidase 2 subunit beta	1.04	0.679	1.13	0.292	1.14	0.081	**1.16**	0.012*	1.01	0.914	1.05	0.584
**Antioxidant**	gi|32189392	**peroxiredoxin-2 isoform a**	1.34	0.096	1.2	0.268	1.28	0.15	1.16	0.454	**1.19**	0.026*	1.28	0.146
	gi|4505591	**peroxiredoxin-1**	1.01	0.871	0.95	0.379	1.01	0.871	0.96	0.479	1.07	0.223	**1.17**	0.021*
**Cell Growth Regulation**	gi|4503057	**alpha-crystallin B chain**	1.04	0.429	1.02	0.718	1.04	0.45	0.99	0.931	**1.24**	0.002*	**1.26**	0.043*
	gi|19743823	integrin beta-1 isoform 1A precursor	1.32	0.102	1.05	0.81	1.14	0.259	**1.52**	0.038*	1.35	0.069	1.08	0.829
**Fatty Acid Metabolism**	gi|4557585	**fatty acid-binding protein, brain**	0.85	0.22	0.84	0.176	0.8	0.228	0.87	0.353	0.9	0.247	**0.83**	0.049*
	gi|4758504	3-hydroxyacyl-CoA dehydrogenase	1.17	0.445	1.09	0.795	1.33	0.097	**1.57**	0.015*	1.18	0.49	1.14	0.381
**Energy Metabolism**	gi|19923437	GTP:AMP phosphotransferase	1.4	0.083	1.27	0.371	**1.46**	0.047*	1.14	0.44	1.43	0.052	1.43	0.149

Cells were incubated with plasma in two 24 well plates, 3 wells for each of the plasma types were pooled from each plate to obtain two biological replicates for the 8-plex iTRAQ experiment. iTRAQ reporter ratios and p-values for altered proteins are shown for both replicates. Proteins found to be dysregulated in MCI and AD treated cells are shown in table. Proteins dysregulated only in cells treated with AD plasma are highlighted in bold. Full list of identified proteins can be found in S2 Table.

* p ≤ 0.05 vs Fetal Bovine Serum Control

**Table 5:** STRING v9.1 analysis of the 27 proteins deregulated only in glia exposed to AD plasma (shown in Table **4**). for enrichment in gene ontology biological processes. Glucose metabolism was found to be the most significant biological process, and is also highlighted in the STRING network map (Fig. 5).

**Molecular Function Enrichment GO_ID**	**Term**	**Number of Proteins**	**p-value**	**p-value FDR**	**p-value Bonferroni**
GO:0003723	RNA Binding	17	1.94E-12	5.34E-09	7.51E-09
GO:0044822	Poly(A) RNA Binding	16	2.75E-12	5.34E-09	1.07E-08
GO:0008379	Thioredoxin peroxidase activity	2	8.88E-06	9.22E-03	3.45E-02
GO:0003676	Nucleic acid binding	16	9.50E-06	9.22E-03	3.69E-02
GO:0051920	Peroxiredoxin activity	2	2.95E-05	2.29E-02	1.15E-01
**Biological Process Enrichment GO_ID**	**Term**	**Number of Proteins**	**p-value**	**p-value FDR**	**p-value Bonferroni**
GO:0006006	Glucose metabolic process	9	1.42E-12	1.76E-08	1.76E-08
GO:0019318	Hexose metabolic process	9	7.28E-12	4.51E-08	9.02E-08
GO:0005996	Monosaccharide metabolic process	9	3.63E-11	1.50E-07	4.49E-07
GO:0046364	Monosaccharide biosynthetic process	6	2.23E-10	6.89E-07	2.75E-06
GO:0016051	Carbohydrate biosynthetic process	7	7.56E-10	1.87E-06	9.35E-06
GO:0006094	Gluconeogenesis	5	5.43E-09	1.12E-05	6.73E-05
GO:0019319	Hexose biosynthetic process	5	9.29E-09	1.64E-05	1.15E-04
GO:0042542	Response to hydrogen peroxide	4	2.30E-05	3.56E-02	2.85E-01
GO:0022614	Membrane to membrane docking	2	3.13E-05	4.30E-02	3.87E-01
GO:0016584	Nucleosome positioning	2	6.56E-05	8.12E-02	8.12E-01
**Cellular Component Enrichment GO_ID**	**Term**	**Number of Proteins**	**p-value**	**p-value FDR**	**p-value Bonferroni**
GO:0070062	Extracellular vesicular exosome	17	1.04E-11	5.02E-09	1.51E-08
GO:0044421	Extracellular region part	18	1.23E-09	4.44E-07	1.77E-06
GO:0005576	Extracellular region	18	1.08E-07	3.06E-05	1.57E-04
GO:0031988	Membrane-bounded vesicle	15	1.27E-07	3.06E-05	1.84E-04
GO:0031982	Vesicle	15	1.83E-07	3.77E-05	2.64E-04
GO:0043233	Organelle lumen	16	2.50E-07	4.32E-05	3.62E-04
GO:0042470	Melanosome	5	2.98E-07	4.32E-05	4.32E-04
GO:0005829	Cytosol	15	1.22E-06	1.47E-04	1.77E-03
GO:0070013	Intracellular organelle lumen	13	5.33E-05	5.93E-03	7.71E-02
GO:0031254	Cell trailing edge	2	7.00E-05	6.76E-03	1.01E-01
GO:0001931	Uropod	2	7.00E-05	6.76E-03	1.01E-01
GO:0030016	Myofibril	4	1.41E-04	1.28E-02	2.05E-01
GO:0016323	Basolateral plasma membrane	4	1.51E-04	1.29E-02	2.19E-01
GO:0043292	Contractile fiber	4	1.66E-04	1.33E-02	2.40E-01
GO:0060205	Cytoplasmic membrane-bound vesicle lumen	3	2.64E-04	1.91E-02	3.82E-01
GO:0031983	Vesicle lumen	3	2.64E-04	1.91E-02	3.82E-01
GO:0031528	Microvillus membrane	2	2.98E-04	2.05E-02	4.31E-01
GO:0016023	Cytoplasmic membrane-bounded vesicle	7	3.68E-04	2.42E-02	5.32E-01
GO:0005719	Nuclear euchromatin	2	5.70E-04	3.43E-02	8.24E-01
GO:0000791	Euchromatin	2	7.98E-04	4.62E-02	1.00E+00

## Discussion

A variety of studies have looked at the effects of plasma on cell cultures in different diseases. For example, Brewer et al found 24 hr exposure of human serum from AD patients to rat hippocampal neurons increased four molecular markers characteristic of Alzheimer senile plaques and neurofibrillary tangles [19]. Another study has shown that Parkinsonian serum has complement-dependent toxicity to rat dopaminergic neurons [50]. A study using a differentiated neuronal cell line investigated the susceptibility of neuronal cells to human complement. It was found that human serum caused lysis of the neurons by complement, as tested by cell viability. The effect was lost when cells were treated with complement-depleted serum by heat inactivation [51].

Our cell culture results also showed that the loss of cell viability and reduction in cell proliferation caused by AD plasma can be prevented by inhibiting the activity of plasma complement proteins. Alterations in peripheral proteins may reflect changes in the brain, especially since damage to the blood-brain-barrier (BBB) resulting in increased permeability has been reported in MCI and AD [52,53]. This suggests that complement may have the capacity to play a role in the cell loss seen in AD. Complement factors may work synergistically to cause loss in cell viability. We observed reduced cell viability when cells were exposed to the complement standard mixture, as compared to addition of single complement factors (Table 2). However in all cases we observed a downward trend in cell viability as complement concentration levels increased regardless of the number of complement proteins present. The data achieved statistical significance with exposure to as few as two complement factors (Table 2), indicating that the full spectrum of complement proteins are not necessary for cytotoxicity. Indeed it has been found that treatment of a transgenic mouse model with an agonist to a single complement receptor, C5aR, decreased pathology and improved behavioural performance [54].

There is significant evidence for the involvement of inflammation in the pathogenesis of Alzheimer’s disease. In the AD brain, damaged neurons and highly insoluble Aβ peptide deposits and NFTs provide stimuli for inflammation [3,14]. Various neuroinflammatory mediators including complement activators and inhibitors, chemokines, cytokines, radical oxygen species and inflammatory enzymes have been shown to be altered in AD [3,14]. Another prominent feature of AD neuropathology is the association of activated proteins of the classical complement pathway with the lesions. The full-range of classical pathway complement proteins from C1q to C5b-9, known as the membrane attack complex, has been found highly localised with Aβ deposits in neuritic plaques [55,56]. It is also present in dystrophic neurites in AD. The fact that complement activation has progressed until the final membrane attack complex stage and the observation that complement regulators have also been found in association with the AD lesions indicates a disturbance in the regulatory mechanisms controlling complement activation in this disease [57–60].

Aβ itself can induce complement-mediated toxicity against neurons in culture, suggesting that Aβ-induced complement activation may contribute to the neuropathogenesis of AD [56,61]. Hyperphosphorylated tau protein, the main component of NFTs, is also a potent stimulator of the complement cascade. Purified NFTs have been shown to activate the complement system in plasma, resulting in a significant increase in levels of membrane attack complexes [62]. Tau and Aβ are both able to increase inflammatory responses and cytokine production. Since the complement system is strongly activated in AD, it could possibly participate either in the exacerbation or amelioration of the pathology. Because Aβ deposits and extracellular NFTs are present during early preclinical until terminal stages of AD, their ability to activate complement provides a mechanism for initiating and sustaining chronic, low-level inflammatory responses that may accumulate over the disease course. This supports the idea that the complement system cascade intervention might be a useful pharmacological approach to treat early stages of AD.

Proteomic analysis of MCI and AD plasma in this study revealed a number of proteins which were significantly altered between the three groups. The majority of these proteins were acute phase reactants, including proteins which were related to the complement system (Table 3). This supports the results from other published studies using mainly proteomic techniques which have shown changes in complement protein levels and other acute phase proteins in AD plasma [5,47,63,64]. A summary of proteins found altered in such studies is provided in Table 6 and some of the proteins found in our study overlap with those found by other groups using larger cohorts of patients. One study has also shown a correlation between brain hippocampal volume changes and plasma levels of acute phase proteins, including complement [63].

**Table 6:** Summary of previous studies showing changes in acute phase proteins in Alzheimer’s disease.

Name	Site of effect	Function	Modification	Reference
Alpha-2-macroglobulin	plasma	Inhibitor of coagulation; inhibitor of fibrinolysis	Increased in MCI and AD	[5,47]
Complement C3	plasma	Most abundant protein of the complement system, enhances response	Increased in AD	[101–104]
Complement C4	plasma	Protein involved in the complement system and undergoes cleavage	Increased in AD	[5,105]
C4b-binding protein	plasma	Inhibits C4 and binds necrotic cells	Decreased in MCI and AD	[47]
Complement C5	plasma	Fifth component of the complement pathway	Increased in MCI	[47]
Complement C9	mRNA and protein levels, vascular amyloid deposits	Involved in MAC formation	Increased in AD brain areas, increased deposition in vascular plaques	[101,102,106]
Complement factor H	plasma	Regulation of alternative pathway of the complement system, ensuring no damage to host tissue	Increased in AD	[5,107]
Fibrinogen	plasma	Involved in blood clotting	Decreased in AD	[47,104,108,109]
Haptogloblin	Plasma, CSF	Binds free haemoglobin thereby reducing its oxidative activity	Increased in AD plasma, decreased in CSF of MCI and AD patients. Other studies show increase in CSF of AD	[110,111]
Hemopexin	plasma and CSF	Binds heme, preserves iron levels in the body	Increased in AD	[105,112,113]
Thrombin	Brain tissue, amyloid plaques, neurofibrillary tangles	Coagulation protein that converts fibrinogen into fibrin, also catalyses other coagulation related reactions	Increased in AD	[114–116]
Transthyretin	Plasma, CSF	Carrier of the thyroid hormone thyroxine	Decreased in AD	[47,112,117,118]

Interestingly one of the complement proteins that was reduced in the AD plasma, complement 4 binding protein (C4BP) is a complement inhibitor which is detected in Aβ plaques and on apoptotic cells in the AD brain [65]. *In vitro*, C4BP binds apoptotic and necrotic but not viable brain cells. It also binds to Aβ(1–42) peptide directly and limits the extent of complement activation by Aβ [65]. C4BP levels in CSF of dementia patients and controls were low compared to levels in plasma and correlated with CSF levels of other inflammation-related factors [65]. Therefore it possibly protects against excessive complement activation in AD brains.

Fibronectin is present in plaques of AD brains and may modify biosynthesis of APP in microglia [66]. Addition of Aβ to cultured astrocytes has been shown to induce a marked increase in the production of fibronectin [67]. This suggests that *in vivo* fibronectin accumulation in senile plaques may be the result, at least in part, of the response of reactive astrocytes to the presence of Aβ. Fibrinogen is associated with an increased risk of AD and vascular dementia [68]. Our study found fibronectin and fibrinogen to be significantly increased in the AD group compared to controls (Table 3).

Furthermore, we have also shown that treatment with AD plasma can affect cellular bioenergetics in a microglial cell line, by increasing glycolysis to compensate for declining oxygen consumption and mitochondrial respiration (Fig. 2). The reduction in cerebral glucose metabolism, as measured by FDG-PET, is a common diagnostic tool for AD. Positron emission tomography (PET) imaging has identified a strong correlation between the spatial distribution of increased glycolysis, and Aβplaques in the AD brain [69]. It is estimated that aerobic glycolysis accounts for up to 90% of glucose consumed [70]. By contrast, a recent neuroimaging study which correlated multimodal neuronal parameters including glucose metabolism and hippocampal volume with Aβ deposition in cognitively normal older individuals, did not find any association between the multimodal neurodegenerative biomarkers [71]. However, diminished neuronal integrity and cognitive function correlated with an increased Aβ burden in brain regions that are most affected by AD pathology [71]. Increased glycolysis was associated with better verbal episodic memory in individuals with elevated amyloid levels in another study [72]. The increased shift towards glycolysis may occur in regions of the brain most vulnerable to insult, or may occur in response to Aβ accumulation during ageing. Loss of this protective mechanism may increase the vulnerability of certain brain regions to Aβ-induced neurotoxicity.

Quantitative iTRAQ analysis of glial cells treated with human AD plasma showed the highest number of dysregulated proteins (Table 4). The most significantly enriched biological process was glucose metabolism (Fig. 5 and Table 5), and a significant number of upregulated glycolytic proteins were found (highlighted in bold in Fig. 4), which is in agreement with the ECAR effect we found in cellular biogenetics using mitochondrial function assays (Fig. 2). Increased expression of glyceraldehyde-3-phosphate dehydrogenase, phosphoglycerate kinase, enolase, aldolase and pyruvate kinase may increase glycolytic flux leading to the accumulation of pyruvate and thus stimulating anaerobic metabolism to lactic acid. We found an increased level of LDH activity in the cell culture media and an increase in extracellular acidification, as indicated by the increase in ECAR in the microglial cells exposed to human AD plasma (Fig. 2). These findings suggest a role for mitochondrial bioenergetic deficits in AD pathogenesis. Our study is consistent with previous PET metabolic analyses in individuals with AD, MCI, or incipient to late AD [73]. Our findings are also consistent with microarray analyses and activity assays of ageing, incipient AD, and AD human samples and rodent models which indicate that genes and the catalytic activity of several glycolytic enzymes are altered in AD or MCI patients [74,75]. Similarly, increased amyloid production and nerve cell atrophy have been shown to induce mitochondrial dysfunction [76]. Overexpression of pyruvate dehydrogenase kinase and lactate dehydrogenase in neurons has been shown to provide resistance to Aβ toxicity and reduces mitochondrial respiration and oxidative stress [77]. Previous proteomic studies have also revealed that enzymes involved in energy metabolism show altered oxidative modification in the AD brain [78]. A recent study has also shown a number of proteins significantly oxidised in the Down syndrome brain with and without AD pathology [79]. A significant number of proteins involved in energy metabolism were identified including some of the glycolysis enzymes which we found altered in our study.

We also report an increase in the protein expression of the enzyme transketolase in microglial cells in response to human AD plasma. Transketolase is a thiamine-dependent enzyme which catalyses the first reaction in the pentose phosphate pathway. Transketolase alterations have been previously identified in (i) several probable AD patients regardless of age-of-onset and severity of disease; (ii) all early-onset AD patients and APOE ε4/4 carriers; and (iii) nearly half of asymptomatic AD relatives [80]. Increased transketolase activity has also been correlated with increased levels of BACE1, the key rate-limiting enzyme for the production of the Aβ peptide [80].

Increased oxidative stress, mitochondrial dysfunction and alterations to energy metabolism have all been implicated as early events in the pathogenesis of AD. Cellular models for AD have been shown to display functional impairment of the mitochondrial respiratory chain, and a decrease in oxygen respiration and ATP production [81]. All functional measures highlight biogenetic impairment of the AD cells with a correlation to the accumulation of amyloid peptides [81].

Factors which induce the upregulation of glycolysis in glial cells treated with AD plasma remain unclear. Apart from the acute phase proteins found altered in our study, small molecules in plasma may also play a role in the upregulation of glycolytic enzymes. A recent study of metabolomic profiling of plasma from an AD mouse model found significant changes to several metabolites involved in energy metabolism, linking neuroinflammation with metabolic disturbance in AD [82]. Since oxidative stress is thought be an important factor in the pathogenesis of AD, the effects of this may also be detected in the circulation in levels of markers such as isoprostanes. Isoprostanes have been shown to be elevated in the AD brain and CSF [83], however recent evidence suggests this may not be reflected in plasma [84], consequently additional work is warranted. Aerobic glycolysis has also been correlated spatially with amyloid deposition in AD brains [69]. It has also been shown that elevated levels of the enzymes pyruvate dehydrogenase kinase and lactate dehydrogenase provide resistance to amyloid and other neurotoxins [77]. The ability of the brain to maintain expression of these enzymes involved in mitochondrial energy metabolism may explain why some individuals could show high levels of amyloid deposition without neurodegeneration [77,85–87]. In our study, the indicators of increased glycolysis in microglia may be a compensatory action caused by the loss of cell viability and mitochondrial function following exposure to AD plasma. Altered activities of key glycolytic enzymes have also been found in hippocampal, frontal and temporal cortex of AD brains and thought to possibly be related to the astrocytosis that occurs in AD [74].

Chaperone proteins are thought to be involved in the pathogenesis of several neurodegenerative and amyloidogenic diseases [88,89]. A number of chaperone proteins such as protein disulfide isomerases were found to be downregulated in cells treated with MCI and AD plasma. Protein disulfide isomerases can inhibit the aggregation of misfolded proteins and are also involved in modulating apoptosis and endoplasmic reticulum redox balance [90]. It has been shown that the proinflammatory activation of microglia suppresses mitochondrial function and increases glycolysis and overexpression of mitochondrial chaperone mortalin can attenuate this effect [91]. Heat shock proteins are chaperone proteins which have a important impact on the proteotoxic effects of tau and Aβ accumulation. Immunohistochemical studies and expression analyses in AD brain tissue have shown that expression levels of a number of heat shock proteins are upregulated and it has been hypothesised that this effect may be due to a hybridisation of activated glia and dysregulated/stressed neurons [92,93]. Dysregulated chaperone proteins in the cells in our study may reflect a homeostatic attempt to clear toxic plasma proteins and protect mitochondrial function.

Cytoskeletal proteins are another group of altered proteins identified in our iTRAQ proteomics data (Table 4). Nestin expression is seen during pathological situations and is a marker of cell proliferation and is reduced in the cells treated with AD plasma, supporting our MTT data (Table 1 and Fig. 1). Ezrin and moesin are involved in crosslinking actin filaments with plasma membranes and stabilising microtubules respectively [94,95] and both were found to be upregulated in cells treated with AD plasma. The antioxidant proteins peroxiredoxins were also found to be elevated in cells exposed to AD plasma which may also be another indication of a compensatory mechanism to attempt to attenuate the toxic effects of AD plasma.

In conclusion, this study shows that plasma expression levels of acute phase proteins are altered in AD and MCI, supporting a role for increased inflammatory activity in this disease which is detectable in the plasma. Cells exposed to AD plasma show an upregulation of glycolysis possibly as a compensatory mechanism in response to compromised mitochondrial function. Together our observations lend support to an emerging body of evidence that inflammation and metabolism are closely linked processes, which are regulated by transcriptional and protein translation events [96,97]. In the CNS, complement proteins are synthesised by a variety of cells including neurons, microglia, astrocytes, oliogendrocytes and endothelial cells [98]. Since disruptions in the blood-brain-barrier have been reported in AD there is a possible source of increased complement levels in the AD brain from plasma. It is however likely that there may be other thermolabile factors in disease plasma which facilitate the cytotoxic and glycolytic effects in microglia, one example may be micro RNAs as they are emerging as important factors in neurogenesis, synaptic plasticity and AD [99,100]. Other yet to be characterised substances may also make a significant contribution. This study shows that the use of biological assays in combination with proteomic analysis may help uncover possible mechanisms of disease and may be complementary techniques to validate cellular changes and effects in a range of biological samples.

## Supporting Information

S1 Fig Scaffold peptide false discovery analysis. Peptide FDR analysis using Scaffold v4 with peptide ROC curve.
(PDF)

S1 Methods Detailed mass spectrometry protocols. Detailed mass spectrometry protocols for proteomics of depleted control, MCI and AD plasma and iTRAQ proteomic analysis of microglial cell lysates treated with control, MCI and AD plasma.
(PDF)

S1 Table Protein summary of human control, MCI and AD plasma. Name and accession of proteins identified in human control, MCI and AD plasma and normalised spectral counts for replicates from Scaffold v4 software.
(PDF)

S2 Table Proteomic of glial cells treated with human plasma. Protein summary of identified proteins with unused score ≥ 1.3 in glial cells treated with control, MCI and AD plasma, showing unused score, percentage coverage, accession number, name, number of peptides, iTRAQ ratios and p-values for both biological replicates. Significantly upregulated proteins are highlighted in red and downregulated proteins in blue.
(PDF)

S3 Table False discovery rate analysis summary for iTRAQ. FDR analysis summary at the protein, peptide and spectral levels for iTRAQ experiment from Protein Pilot v4.
(PDF)
