# Supplementary material for: Upregulation of Glycolytic Enzymes, Mitochondrial Dysfunction and Increased Cytotoxicity in Glial Cells Treated with Alzheimer’s Disease Plasma
Source: PLoS One. 2015 Mar 18;10(3):e0116092. doi: 10.1371/journal.pone.0116092 (PMC4364672; doi:10.1371/journal.pone.0116092)

Scaffold False Discovery Rate Analysis

Scaffold Version: Scaffold_4.3.4

Protein Grouping Strategy: Experiment-wide grouping with binary peptide-protein weights

Peptide Thresholds: 95.0% minimum

Protein Thresholds: 99.0% minimum and 2 peptides minimum

107 Proteins identified at 99.0% minimum

Peptide FDR: 5.2% (Prophet)

Figure S1 - Scaffold Peptide ROC Plot

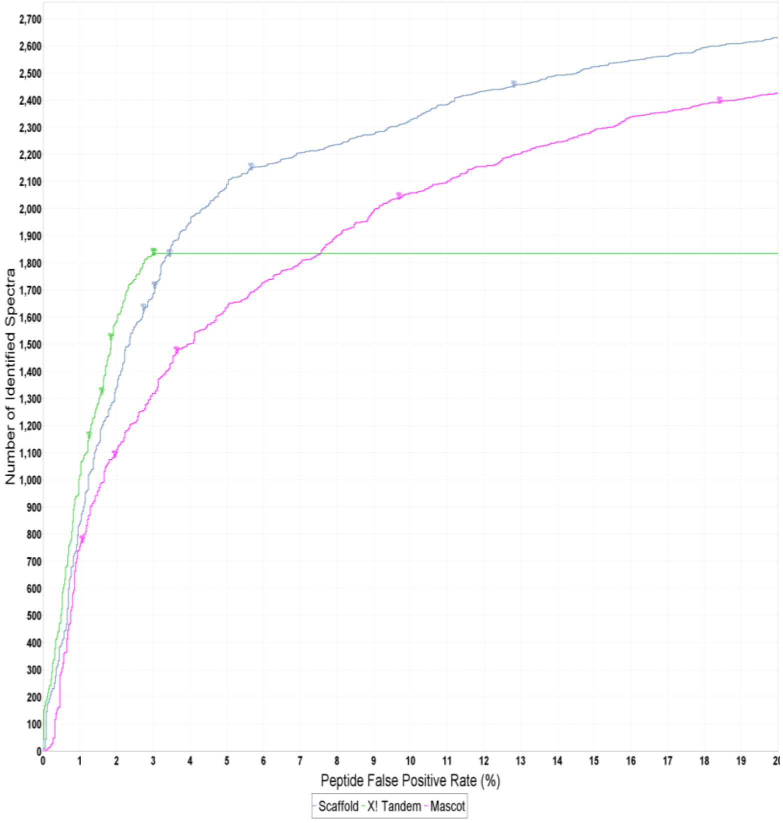

Supplement: S1 Fig — Peptide FDR analysis using Scaffold v4 with peptide ROC curve. (PDF) [file pone.0116092.s001.pdf]
