# Supplementary material for: Upregulation of Glycolytic Enzymes, Mitochondrial Dysfunction and Increased Cytotoxicity in Glial Cells Treated with Alzheimer’s Disease Plasma
Source: PLoS One. 2015 Mar 18;10(3):e0116092. doi: 10.1371/journal.pone.0116092 (PMC4364672; doi:10.1371/journal.pone.0116092)
Supplement: S1 Methods — Detailed mass spectrometry protocols for proteomics of depleted control, MCI and AD plasma and iTRAQ proteomic analysis of microglial cell lysates treated with control, MCI and AD plasma. (PDF) [file pone.0116092.s002.pdf]

Supplementary Methods S1

Detailed Mass Spectrometry Protocol for Proteomic Analysis of Human Control, MCI and AD Plasma

Digested peptides were separated by nano-LC using a Cap-LC autosampler system (Waters, Milford MA). Samples (5 µl) were concentrated and desalted onto a micro C18 precolumn (500 µm x 2 mm, Michrom Bioresources, Auburn, CA) with H_2O:CH_3CN (98:2, 0.05 % HFBA) at 15 ul/min. After a 4 min wash the pre-column was automatically switched (Valco 10 port valve, Houston, TX) into line with a fritless nano column (75 µm x ~12 cm) containing Magic C18 (~10cm, 200Å, Michrom) manufactured according to Gatlin [35]. Peptides were eluted using a linear gradient of H_2O:CH_3CN (98:2, 0.1 % formic acid) to H_2O:CH_3CN (55:45, 0.1 % formic acid) at ~300 nl/min over 30 min. The precolumn was connected via a fused silica capillary (10 cm, 25 µ) to a low volume tee (Upchurch Scientific) where HV (2400 V) was applied and the column tip positioned ~ 1 cm from the Z-spray inlet of a QTof Ultima API hybrid tandem mass spectrometer (Micromass, Manchester, UK). Positive ions were generated by electrospray and the QTof operated in data dependent acquisition mode (DDA). A Tof MS survey scan was acquired (m/z 350-1700, 1 s) and the 2 largest multiply charged ions (counts > 20) were sequentially selected by Q1 for MS-MS analysis. Argon was used as collision gas and an optimum collision energy chosen (based on charge state and mass). Tandem mass spectra were accumulated for up to 2 s (m/z 50-2000).

Detailed Mass Spectrometry Protocol for iTRAQ Proteomic Analysis of Cell Lysates Treated with Human Control, MCI and AD Plasma

Chromatography was performed using an LC Packings capillary HPLC system (Dionex, Amsterdam, Netherlands), comprised of an Ultimate pump system, Switchos valve unit and Famos autosampler. Samples (iTRAQ labelled peptide mixtures) were captured onto a C18 pre-column cartridge (500 µm x 2 mm, Michrom Bioresources, Auburn, CA). After a 10 min wash to remove any residual buffers or salts, the pre-column was automatically switched into line with a capillary column (75 µm x ~12 cm) containing C18 reverse phase packing material (ReproSil-Pur 120 C18-AQ, 1.9 µM, Dr Maisch, Ammerbuch-Entringen, Germany). Peptides were eluted using a 240 min linear gradient of H_2O:CH_3CN (98:2, 0.1 % formic acid) to H_2O:CH_3CN (20:80, 0.1 % formic acid) at ~300 nl/min. The precolumn was connected via a fused silica capillary (10 cm, 25 µ) to a low

volume tee (Upchurch Scientific, WA, USA) where high voltage (2300 V) was applied and the column tip positioned ~ 1 cm from the spray inlet of a TripleTOF 5600 hybrid tandem mass spectrometer (ABSciex, CA, USA). Positive ions were generated by electrospray and the instrument operated in information dependent acquisition (IDA) mode. A TOF MS survey scan was acquired (m/z 350-1700, 0.75 s) and the three largest multiply charged ions (counts > 20, charge state ≥ 2 and ≤ 4) were sequentially selected by Q1 for MS-MS analysis. Nitrogen was used as collision gas and an optimum collision energy automatically chosen (based on charge state and mass). Tandem mass spectra were accumulated for up to 2.5 s (m/z 65-2000).
