# Supplementary material for: Upregulation of Glycolytic Enzymes, Mitochondrial Dysfunction and Increased Cytotoxicity in Glial Cells Treated with Alzheimer’s Disease Plasma
Source: PLoS One. 2015 Mar 18;10(3):e0116092. doi: 10.1371/journal.pone.0116092 (PMC4364672; doi:10.1371/journal.pone.0116092)
Supplement: S1 Table — Name and accession of proteins identified in human control, MCI and AD plasma and normalised spectral counts for replicates from Scaffold v4 software. (PDF) [file pone.0116092.s003.pdf]

Table S1 - Normalised Total Spectra Counts for Control, MCI and AD plasma using Scaffold v4.3.4 (significantly altered proteins in bold)

			AD plasma replicates				MCI Plasma replicates				Control plasma replicates			
**#**	**Identified Proteins (107)**	**Accession Number**	**AD1**	**AD2**	**AD3**	**AD4**	**MCI1**	**MCI2**	**MCI3**	**MCI4**	**C1**	**C2**	**C3**	**C4**
1	Alpha-2-macroglobulin precursc	gi|112911 (+2)	100.27	99.586	119.25	107.24	131.22	99.621	116.67	104.21	149.75	162.1	122.29	98.57
2	apolipoprotein B precursor [Hor	gi|105990532 (+1)	93.535	112.74	89.439	89.941	131.95	124.53	90.063	73.558	74.876	93.006	57.683	95.39
3	Chain A, Human Serum Albumin	gi|122920512 (+4)	86.052	115.24	83.051	65.726	137.05	132.62	75.735	67.428	81.562	90.349	73.834	71.543
4	complement component 3 prec	gi|115298678	94.283	84.554	112.86	103.78	110.08	104.6	112.58	112.38	116.33	102.31	124.6	111.29
5	keratin 1 [Homo sapiens], gi|39	gi|11935049	53.876	57.622	83.051	53.619	35.721	39.226	96.204	149.16	52.815	47.832	101.52	71.543
6	fibrinogen, alpha polypeptide is	gi|11761629 (+2)	39.659	30.69	40.461	32.863	32.076	29.886	47.078	34.736	31.421	30.559	41.532	41.336
7	proapolipoprotein	gi|178775	41.155	35.701	27.684	34.593	46.657	39.848	28.656	28.606	63.511	53.146	23.073	31.797
8	hCG2001591 [Homo sapiens]	gi|119623974 (+2)	41.155	35.074	29.813	39.782	39.367	43.584	30.703	32.692	36.77	39.195	29.995	42.926
9	Chain A, Trypsin In Complex Wit	gi|110590762 (+3)	23.945	17.537	10.648	17.296	29.889	29.264	12.281	6.1298	40.112	35.209	16.151	22.258
**10**	**fibrinogen, beta chain prepropr**	**gi|70906435**	**36.666**	**30.69**	**63.885**	**41.511**	**34.263**	**31.754**	**34.797**	**42.909**	**32.758**	**27.902**	**50.761**	**47.695**
11	keratin 10 (epidermolytic hyperl	gi|119581085 (+2)	26.938	40.711	53.238	13.837	18.225	15.566	47.078	63.342	28.079	20.594	64.605	28.617
12	similar to Keratin, type I cytoske	gi|114667176	19.455	21.295	27.684	20.756	13.851	17.434	32.75	67.428	20.725	21.923	34.61	12.719
13	fibrinogen gamma chain, isoforr	gi|119625320 (+4)	29.183	23.174	12.777	13.837	28.431	25.528	20.469	12.26	28.079	30.559	18.459	25.437
14	keratin 2 [Homo sapiens], gi|64	gi|47132620	29.183	27.558	63.885	19.026	19.683	21.169	55.266	55.169	31.421	24.58	46.146	27.027
15	ceruloplasmin (ferroxidase), iso	gi|119599289 (+3)	23.945	21.921	17.036	13.837	23.328	20.547	18.422	12.26	16.713	17.937	13.844	17.488
**16**	**Fibronectin 1 [Homo sapiens], g**	**gi|109658664 (+11)**	**32.924**	**38.206**	**29.813**	**27.674**	**19.683**	**18.056**	**14.328**	**8.1731**	**18.719**	**19.266**	**18.459**	**19.078**
17	Complement factor H precursor	gi|158517847 (+3)	20.204	13.779	23.425	22.485	19.683	7.4716	22.516	20.433	14.039	18.601	23.073	19.078
18	inter-alpha (globulin) inhibitor H	gi|119606782 (+3)	19.455	22.548	8.518	15.567	9.4771	10.585	12.281	8.1731	11.365	15.944	11.537	12.719
19	hemopexin [Homo sapiens], gi|	gi|11321561 (+6)	11.224	12.527	10.648	5.1889	15.309	14.943	14.328	8.1731	16.045	16.608	11.537	9.539
20	inter-alpha (globulin) inhibitor H	gi|119585668 (+3)	20.952	10.648	17.036	13.837	14.58	11.83	14.328	10.216	13.371	12.622	9.2293	20.668
21	Chain A, Crystal Structure Of Th	gi|28373620 (+1)	14.217	11.9	8.518	10.378	11.664	13.075	8.1875	12.26	15.376	13.951	9.2293	15.898
22	inter-alpha (globulin) inhibitor H	gi|119585664 (+1)	13.469	20.042	12.777	10.378	10.206	12.453	16.375	16.346	7.3539	11.294	16.151	11.129
23	Chain A, Tertiary Structures Of T	gi|2098257 (+3)	15.714	12.527	17.036	15.567	17.496	13.698	10.234	10.216	16.713	15.28	18.459	17.488
24	Complement factor B [Homo sa	gi|13278732 (+2)	11.224	8.7686	4.259	5.1889	6.5611	9.9621	2.0469	4.0866	10.028	11.294	4.6146	6.3594
25	Chain I, 2.5a Crystal Structure O	gi|52695711 (+1)	12.721	11.274	8.518	12.107	13.122	11.207	6.1407	6.1298	10.028	7.972	6.922	17.488
26	apolipoprotein A-IV precursor [H	gi|178757 (+2)	11.224	9.3949	2.1295	1.7296	10.206	8.7168	4.0938	4.0866	9.3595	8.6363	2.3073	4.7695
27	gelsolin isoform 32	gi|114626427 (+2)	11.972	7.5159	4.259	10.378	10.206	9.9621	8.1875	10.216	8.691	9.9649	4.6146	6.3594

28	Chain A, Crystal Structure Of Cle	gi|443345	6.7345	5.6369	12.777	19.026	5.1031	6.8489	14.328	10.216	4.0112	6.6433	16.151	12.719
**29**	**complement component 1 inhi**	**gi|114642584 (+8)**	**9.7276**	**7.5159**	**10.648**	**17.296**	**8.0191**	**7.4716**	**6.1407**	**8.1731**	**8.0225**	**9.9649**	**9.2293**	**12.719**
30	Complement component 5 [Hor	gi|109731812 (+2)	5.9862	6.2633	4.259	8.6482	5.1031	5.6037	4.0938	6.1298	8.0225	8.6363	2.3073	11.129
**31**	**Alpha-1B-glycoprotein precurso**	**gi|46577680 (+1)**	**6.7345**	**6.2633**	**10.648**	**6.9185**	**8.0191**	**6.8489**	**10.234**	**8.1731**	**6.6854**	**6.6433**	**4.6146**	**6.3594**
32	plasminogen	gi|387026 (+1)	6.7345	8.7686	2.1295	0	4.3741	8.7168	4.0938	0	5.3483	8.6363	6.922	1.5898
33	Kininogen 1 [Homo sapiens]	gi|37748641 (+2)	8.9794	6.2633	6.3885	12.107	9.4771	4.981	2.0469	6.1298	4.6798	5.979	11.537	6.3594
34	apolipoprotein E mutant E3K	gi|364011 (+1)	6.7345	5.6369	10.648	5.1889	4.3741	5.6037	8.1875	6.1298	4.0112	5.3146	9.2293	9.539
35	alpha-1-microglobulin/bikunin p	gi|4502067 (+1)	6.7345	6.8896	4.259	5.1889	5.8321	6.2263	4.0938	2.0433	5.3483	4.6503	6.922	3.1797
**36**	**coagulation factor II (thrombin)**	**gi|119588383 (+4)**	**4.4897**	**5.6369**	**6.3885**	**8.6482**	**5.8321**	**6.2263**	**4.0938**	**6.1298**	**8.691**	**9.3006**	**13.844**	**9.539**
37	Angiotensinogen (serpin peptida	gi|15079348 (+10)	9.7276	8.1422	10.648	8.6482	8.0191	7.4716	10.234	8.1731	8.0225	8.6363	4.6146	7.9492
38	alpha-1-acid glycoprotein 1 prec	gi|1197209 (+2)	6.7345	6.2633	4.259	3.4593	7.2901	5.6037	4.0938	4.0866	2.0056	5.979	2.3073	6.3594
39	Alpha-2-HS-glycoprotein precurs	gi|112910 (+2)	6.7345	3.758	6.3885	6.9185	8.0191	4.3584	8.1875	2.0433	6.6854	5.3146	11.537	11.129
40	haptoglobin, isoform CRA_a [Ho	gi|119579598	9.7276	2.5053	2.1295	3.4593	6.5611	7.4716	4.0938	2.0433	3.3427	1.993	2.3073	1.5898
41	apolipoprotein H precursor [Hor	gi|153266841 (+2)	5.238	4.3843	0	1.7296	2.916	4.3584	2.0469	0	2.6742	5.3146	0	4.7695
42	apolipoprotein J precursor	gi|178855 (+6)	4.4897	1.879	2.1295	1.7296	3.645	2.4905	4.0938	4.0866	2.6742	1.993	0	7.9492
43	Chain A, The Structure Of Penta	gi|576259	4.4897	5.0106	4.259	3.4593	3.645	3.1132	6.1407	4.0866	4.0112	5.979	2.3073	3.1797
44	apolipoprotein D, apoD [human,	gi|619383	2.9931	2.5053	4.259	3.4593	2.916	1.8679	4.0938	2.0433	2.6742	2.6573	4.6146	0
45	retinol binding protein 4, plasma	gi|119570453 (+6)	2.9931	2.5053	2.1295	1.7296	1.458	3.7358	0	0	2.6742	2.6573	2.3073	1.5898
46	paraoxonase 1 [Homo sapiens],	gi|19923106	4.4897	2.5053	4.259	1.7296	5.1031	2.4905	4.0938	4.0866	4.0112	3.3216	4.6146	1.5898
47	Vitronectin [Homo sapiens], gi|:	gi|13477169 (+2)	2.9931	3.1316	6.3885	3.4593	3.645	4.3584	4.0938	6.1298	3.3427	4.6503	6.922	3.1797
**48**	**complement component 4 bind**	**gi|4502503**	**3.7414**	**3.1316**	**4.259**	**6.9185**	**3.645**	**3.7358**	**4.0938**	**2.0433**	**4.6798**	**2.6573**	**4.6146**	**4.7695**
49	histidine-rich glycoprotein precu	gi|4504489	4.4897	6.8896	0	1.7296	1.458	1.8679	4.0938	4.0866	2.0056	1.993	6.922	3.1797
50	Keratin, type I microfibrillar 48 k	gi|125090	0	0	0	17.296	0	6.2263	0	0	0.6685	3.986	0	0
51	similar to afamin	gi|114594313 (+1)	2.9931	3.758	2.1295	0	1.458	3.1132	2.0469	4.0866	1.3371	1.993	4.6146	3.1797
52	complement component 6, isofo	gi|119576417 (+4)	4.4897	3.1316	0	6.9185	2.916	1.8679	4.0938	0	2.6742	2.6573	4.6146	1.5898
53	unnamed protein product [Hom	gi|158261613 (+1)	4.4897	3.1316	2.1295	1.7296	3.645	3.7358	2.0469	2.0433	3.3427	2.6573	0	7.9492
54	type II intermediate-filament pr	gi|246276	0	0	0	19.026	0	4.981	0	0	0	0	0	0
55	complement component 7, isofo	gi|119576411 (+4)	3.7414	4.3843	2.1295	3.4593	1.458	3.7358	4.0938	2.0433	2.6742	3.3216	0	1.5898
56	heparin cofactor II isoform 1 [Pa	gi|114685230 (+5)	1.4966	1.2527	2.1295	0	1.458	0.6226	0	2.0433	0.6685	1.3287	2.3073	4.7695
57	complement component 9, isofo	gi|119576390 (+3)	5.238	3.758	2.1295	3.4593	4.3741	3.7358	4.0938	2.0433	2.6742	2.6573	4.6146	6.3594
58	complement component 1, q su	gi|150036344 (+5)	2.9931	1.879	2.1295	1.7296	2.916	1.8679	2.0469	2.0433	2.0056	2.6573	0	3.1797
59	serine (or cysteine) proteinase i	gi|114665618 (+6)	2.2448	1.879	2.1295	3.4593	1.458	1.8679	4.0938	2.0433	2.0056	0.6643	6.922	7.9492
60	unnamed protein product [Hom	gi|16553735 (+2)	2.2448	1.2527	4.259	3.4593	1.458	1.8679	4.0938	2.0433	1.3371	2.6573	4.6146	4.7695

61	inter-alpha (globulin) inhibitor H	gi|119585666 (+5)	1.4966	1.2527	2.1295	1.7296	2.187	1.8679	2.0469	0	1.3371	1.3287	2.3073	3.1797
62	Complement component 8, gam	gi|109731764 (+4)	2.2448	1.2527	2.1295	1.7296	1.458	1.2453	4.0938	2.0433	2.6742	1.3287	0	3.1797
63	unnamed protein product [Hom	gi|1335054	2.2448	0.6263	0	0	1.458	1.2453	8.1875	6.1298	2.0056	1.993	2.3073	3.1797
64	serum amyloid A4, constitutive	gi|10835095 (+3)	2.9931	1.2527	2.1295	0	0	1.8679	0	2.0433	0.6685	0.6643	0	1.5898
65	keratin 5 [Homo sapiens], gi|14	gi|119395754 (+1)	0	10.021	4.259	0	0	0	6.1407	6.1298	5.3483	0	2.3073	6.3594
66	hCG2040284 [Homo sapiens]	gi|119598461 (+2)	2.2448	2.5053	0	0	1.458	2.4905	0	0	2.0056	1.3287	0	0
67	alpha-1-acid glycoprotein 2 prec	gi|29170378 (+2)	1.4966	1.879	4.259	1.7296	3.645	3.7358	2.0469	0	2.0056	3.986	0	1.5898
68	Complement C1r subcomponen	gi|115204 (+6)	2.2448	2.5053	0	1.7296	0.729	1.8679	0	2.0433	1.3371	2.6573	0	1.5898
69	Chain A, Human Apolipoprotein	gi|157831482	0	0	0	5.1889	0	0	2.0469	0	0	0	2.3073	0
70	alpha S1 casein	gi|159793187 (+6)	0	0	4.259	0	0	0	6.1407	8.1731	0	0	6.922	0
71	similar to Ficolin (collagen/fibrir	gi|114555009 (+4)	1.4966	1.879	2.1295	1.7296	1.458	1.2453	2.0469	0	1.3371	1.3287	2.3073	1.5898
72	C-type lectin domain family 3, m	gi|156627579 (+2)	0.7483	1.879	0	1.7296	0.729	1.2453	2.0469	0	2.0056	1.3287	2.3073	3.1797
73	protein S (alpha)	gi|114588068 (+5)	0	0.6263	0	0	0	1.2453	4.0938	0	0.6685	1.3287	0	1.5898
74	Kallikrein B, plasma (Fletcher fac	gi|109659056 (+4)	0	3.1316	0	0	0	1.2453	0	0	2.0056	3.3216	0	0
75	hair type II keratin intermediate	gi|1308	0	0	0	20.756	0	8.0942	0	0	0	2.6573	0	0
76	alpha-2-glycoprotein 1, zinc	gi|114614917 (+6)	2.2448	1.2527	0	0	1.458	0.6226	0	2.0433	0.6685	0.6643	0	1.5898
77	hypothetical protein LOC50742	gi|134085840	0	0	0	17.296	0	7.4716	0	0	0	0.6643	0	0
78	alpha-2-plasmin inhibitor [Homo	gi|115583663 (+5)	0	1.2527	0	0	1.458	0.6226	0	0	1.3371	0.6643	2.3073	0
79	apolipoprotein M [Homo sapien	gi|22091452 (+1)	0.7483	0.6263	0	0	0	1.8679	0	0	0.6685	0	0	0
80	Rubber elongation factor protei	gi|132270	0.7483	0	0	1.7296	0	0	4.0938	2.0433	0	1.3287	2.3073	1.5898
81	complement factor B isoform 1	gi|109070536	7.4828	6.8896	2.1295	3.4593	0	6.8489	0	0	6.6854	6.6433	0	0
82	coagulation factor XII (Hageman	gi|119605410 (+6)	1.4966	0.6263	0	1.7296	0	0	0	0	0	0	0	1.5898
83	hypothetical protein LOC53959	gi|149642659	0	0	0	17.296	0	7.4716	0	0	0	0	0	0
84	Chain A, Thyroxine-Binding Glob	gi|114793604 (+5)	0.7483	1.2527	0	0	0.729	1.2453	0	0	0.6685	0.6643	0	0
**85**	**complement component 2 prec**	**gi|14550407 (+4)**	**2.9931**	**2.5053**	**2.1295**	**3.4593**	**0**	**1.2453**	**0**	**2.0433**	**0**	**0**	**0**	**0**
86	keratin 6A [Homo sapiens]	gi|114644568 (+5)	6.7345	11.274	0	0	0	0	0	6.1298	8.0225	0	0	4.7695
87	pregnancy-zone protein isoform	gi|114643429 (+4)	0	0	0	10.378	14.58	12.453	14.328	12.26	18.051	17.273	0	9.539
88	hypothetical protein isoform 2	gi|114654573 (+4)	1.4966	1.2527	0	0	2.187	1.8679	0	0	0.6685	0	0	1.5898
89	Chain A, Structural Changes Of T	gi|1065033 (+143)	0.7483	0.6263	2.1295	0	0.729	1.2453	0	0	0.6685	0.6643	2.3073	0
90	hemoglobin alpha-1 globin chair	gi|13650074 (+22)	0.7483	0.6263	0	1.7296	0	1.2453	2.0469	0	0	0	0	3.1797
91	lumican isoform 1	gi|114646184 (+4)	0.7483	1.879	0	0	0	0	0	0	0	0	2.3073	0
92	unnamed protein product [Hom	gi|158257796 (+3)	0	2.5053	0	0	0	0	0	0	1.3371	2.6573	0	0
93	complement component 8, beta	gi|119627047 (+4)	1.4966	1.2527	0	1.7296	0.729	0	2.0469	0	0	0	0	1.5898

94	similar to Fibrinogen beta chain	gi|119908847	2.9931	3.1316	0	0	0	3.1132	0	0	3.3427	1.993	6.922	0
95	similar to apolipoprotein F	gi|114644115 (+3)	0.7483	0	0	0	0	1.2453	2.0469	0	0	0.6643	0	1.5898
96	lipoprotein CIII	gi|224917	0	0.6263	4.259	3.4593	0	0	0	0	0	0	4.6146	0
97	Human Vitamin D Binding Prote	gi|18655424 (+1)	13.469	11.9	8.518	10.378	0	13.075	0	12.26	14.039	0	0	15.898
98	complement component 8, alph	gi|114556800 (+5)	0.7483	1.2527	0	1.7296	0	0.6226	0	0	0.6685	0	0	0
99	attractin, isoform CRA_b [Homo	gi|119630936 (+6)	0	0	0	0	0	0	2.0469	2.0433	0	1.3287	2.3073	0
100	keratin associated protein 13.1	gi|41350563	0	0	0	6.9185	0	0.6226	0	0	0	0	0	0
101	hair keratin A1	gi|115495167	0	0	0	13.837	0	6.8489	0	0	0	0	0	0
102	fibulin 1 isoform 3	gi|109094533 (+5)	0.7483	0.6263	0	0	0	0.6226	0	0	1.3371	0	0	0
103	similar to fibrinogen, gamma ch	gi|73977992 (+2)	0	8.1422	0	6.9185	0	0	0	0	0	0	0	12.719
104	hypothetical protein	gi|119922176 (+2)	0	0	0	6.9185	0	0.6226	0	0	0	0	0	0
105	Chain A, Apo-Human Serum Tra	gi|110590597 (+6)	0	1.879	0	0	0	0	0	0	0	0	0	0
106	Chain A, Yeast Alcohol Dehydrog	gi|112491285 (+4)	0	1.2527	0	0	0	0	0	0	0	0	0	0
107	Keratin-associated protein 11-1	gi|52783049	0	0	0	5.1889	0	0	0	0	0	0	0	0

Charge state deconvolution and deisotoping were not performed. All MS/MS samples were analysed using Mascot (Matrix Science, London, UK). Mascot was set up to search the NCBInr database (33055681 entries). Mascot was searched with a fragment ion mass tolerance of 0.20Da and a parent tolerance of 0.25Da. Oxidation of methionine and iodoacetaminde derivative of cysteine were specified as variable modifications.

Scaffold was used to validate MS/MS based peptide and protein identifications. Peptide identifications were accepted if they could be established at greater than 95.0% probability by the Peptide Prophet algorithm. Protein identifications were accepted if they could be established at greater than 99.0% probability and contained at least 2 identified peptides. Protein probabilities were assigned by the Protein Prophet algorithm.
