# Supplementary material for: Upregulation of Glycolytic Enzymes, Mitochondrial Dysfunction and Increased Cytotoxicity in Glial Cells Treated with Alzheimer’s Disease Plasma
Source: PLoS One. 2015 Mar 18;10(3):e0116092. doi: 10.1371/journal.pone.0116092 (PMC4364672; doi:10.1371/journal.pone.0116092)
Supplement: S2 Table — Protein summary of identified proteins with unused score ≥ 1.3 in glial cells treated with control, MCI and AD plasma, showing unused score, percentage coverage, accession number, name, number of peptides, iTRAQ ratios and p-values for both biological replicates. Significantly upregulated proteins are highlighted in red and downregulated proteins in blue. (PDF) [file pone.0116092.s004.pdf]

Table S2 - Protein Summary for iTRAQ Experiment with mircoglia treated with human control, MCI and AD plasma from Protein Pilot v4.0

114: Miroglia treated with Control Plasma replicate 1; **115**: Microglia treated with MCI plasma replicate 1; **116**: Microglia treated with AD plasma replicate 1
117: Microglia treated with Fetal Bovine Serum
118: Microglia treated with Control plasma replicate 2; **119**: Microglia treated with MCI plasma replicate 2; **121**: Microglia treated with AD plasma replicate 2

Upregulated proteins are shown in red and downregulated proteins are shown in blue

Unused	%Cov (95)	Accession	Name	Peptides (95%)	114:117	PVal 114:117	115:117	PVal 115:117	116:117	PVal 116:117	118:117	PVal 118:117	119:117	PVal 119:117	121:117	PVal 121:117
50.27	72.54	gi|7669492	glyceraldehyde-3-phosphate dehydrogenase isoform 1 [Homo sapiens]	62	0.97414	0.52389	0.9826	0.676093	1.273859	3.43E-05	1.02487	0.60275	0.976086	0.65477	1.31801	0.0001
64.83	76.27	gi|4503571	alpha-enolase isoform 1 [Homo sapiens]	62	0.9323	0.11964	0.98826	0.719005	1.192321	0.00027	0.98271	0.75327	0.97853	0.55307	1.22469	0.000105
42.89	55.88	gi|4505763	phosphoglycerate kinase 1 [Homo sapiens]	27	1.07208	0.17501	1.0474	0.279231	1.203913	7.01E-05	1.04557	0.32215	1.062029	0.20679	1.30616	0.000111
38.22	61.48	gi|342187211	fructose-bisphosphate aldolase A isoform 2 [Homo sapiens]	27	0.98961	0.82864	1.01971	0.836449	1.204599	0.00446	1.00107	0.9894	1.000761	0.99085	1.2183	0.001405
77.64	69.11	gi|33286418	pyruvate kinase isozymes M1/M2 isoform a [Homo sapiens]	49	0.97698	0.74623	1.00909	0.869057	1.22076	0.00752	1.02407	0.72615	1.027908	0.59976	1.2245	0.001615
49.96	46.39	gi|4507521	transketolase isoform 1 [Homo sapiens]	34	0.9812	0.68558	1.04368	0.323134	1.130957	0.02218	1.01055	0.88087	0.987174	0.74884	1.15666	0.001681
74.49	23.07	gi|38176300	nestin [Homo sapiens]	42	0.98677	0.77329	0.96911	0.518102	0.943442	0.17346	0.99888	0.98412	1.036204	0.35449	0.85376	0.004498

37.03	38.85	gi|66933005	calnexin precursor [Homo sapiens]	22	0.99713	0.94162	0.94451	0.31514	0.924302	0.26575	0.9798	0.70005	0.932549	0.14875	0.87883	0.010136
59.84	72.51	gi|4503471	elongation factor 1-alpha 1 [Homo sapiens]	54	0.96687	0.49315	0.93464	0.225032	1.131237	0.0241	1.02401	0.75967	0.967436	0.56314	1.24135	0.014366
27.94	29.73	gi|19920317	cytoskeleton-associated protein 4 [Homo sapiens]	17	0.84689	0.04873	0.92014	0.229067	0.736276	0.0001	0.76848	0.01158	0.78562	0.03204	0.76711	0.018819
30.5	47.73	gi|5031973	protein disulfide-isomerase A6 precursor [Homo sapiens]	22	0.9654	0.42869	0.944	0.133813	0.957242	0.54622	0.97927	0.66221	1.002759	0.95171	0.90975	0.019165
20.99	59.3	gi|4505591	peroxiredoxin-1 [Homo sapiens]	15	1.01122	0.8714	1.00843	0.871252	1.071734	0.22303	0.95462	0.37925	0.963405	0.47862	1.1723	0.021238
5.22	13.15	gi|214010226	40S ribosomal protein S24 isoform d [Homo sapiens]	4	0.67916	0.06245	0.75061	0.010725	0.60023	0.06339	0.66105	0.01456	0.664317	0.08515	0.63548	0.021809
18.45	46.15	gi|4506713	ubiquitin-40S ribosomal protein S27a precursor [Homo sapiens]	11	0.98274	0.73773	1.05902	0.285887	0.836245	0.0664	0.82362	0.15854	1.037765	0.59399	0.77292	0.022464
13.76	19.9	gi|66346681	plasminogen activator inhibitor 1 RNA-binding protein isoform 2 [Homo sapiens]	7	1.04273	0.58745	1.00011	0.999016	0.966927	0.6123	0.98868	0.92202	0.927851	0.47639	0.78652	0.023336
21.89	15.51	gi|44680105	caldesmon isoform 1 [Homo sapiens]	11	0.90827	0.1536	0.89843	0.174139	0.874096	0.0854	0.97455	0.77315	0.936821	0.47815	0.85601	0.026671
41.45	38.13	gi|4505257	moesin [Homo sapiens]	23	1.07974	0.26539	1.11677	0.105786	1.224702	0.02088	1.13547	0.24289	1.029872	0.72063	1.20124	0.028037
38.53	62.14	gi|4502101	annexin A1 [Homo sapiens]	23	1.00008	0.99855	0.92705	0.161224	0.872665	0.02261	0.98702	0.84843	1.015028	0.79418	0.8338	0.042088
17.57	59.43	gi|4503057	alpha-crystallin B chain [Homo sapiens]	12	1.03866	0.42864	1.04427	0.449927	1.237261	0.00212	1.02433	0.71835	0.994397	0.93124	1.26081	0.042824
2.32	12.63	gi|54792069	small ubiquitin-related modifier 2 isoform a precursor [Homo sapiens]	2	1.14799	0.29051	1.45937	0.083382	1.231096	0.26043	1.01658	0.94786	1.407796	0.05217	1.28827	0.043238

9.54	13.73	gi|109637759	calpastatin isoform f [Homo sapiens]	7	0.86253	0.228	0.80906	0.239705	0.817004	0.20571	0.74525	0.1378	0.884395	0.46022	0.74361	0.045267
6.64	22.73	gi|4557585	fatty acid-binding protein, brain [Homo sapiens]	4	0.85065	0.21974	0.79912	0.227698	0.90105	0.24714	0.84292	0.17597	0.873763	0.35343	0.83176	0.049333
12.44	24.27	gi|4503143	cathepsin D preproprotein [Homo sapiens]	10	0.95791	0.58116	1.0536	0.628932	1.106723	0.5541	1.06446	0.69411	0.9615	0.74084	1.16945	0.058726
50.95	32.2	gi|153792590	heat shock protein HSP 90-alpha isoform 1 [Homo sapiens]	33	1.03008	0.67865	1.06218	0.512408	1.299528	0.03574	1.04757	0.60122	1.055205	0.4726	1.20463	0.058983
45.71	56.44	gi|21361657	protein disulfide-isomerase A3 precursor [Homo sapiens]	37	0.9619	0.42744	0.89756	0.012721	0.924691	0.11729	0.98181	0.65258	0.995083	0.87086	0.89188	0.059792
14.84	80	gi|5032057	protein S100-A11 [Homo sapiens]	16	1.04077	0.52822	0.94971	0.423999	0.863005	0.05467	1.04763	0.46558	0.98643	0.82636	0.86681	0.060101
15.61	63.49	gi|31543380	protein DJ-1 [Homo sapiens]	8	0.87038	0.05065	1.0421	0.587302	1.041927	0.75125	0.95154	0.45415	0.994383	0.95366	1.16939	0.069841
18.16	57.3	gi|66392203	NME1-NME2 protein [Homo sapiens]	11	1.05003	0.63669	1.05967	0.740323	1.119333	0.36696	1.10984	0.37772	1.046986	0.7303	1.22055	0.086404
25.24	38.67	gi|20149594	heat shock protein HSP 90-beta [Homo sapiens]	33	0.94769	0.31531	1.04297	0.30697	1.114919	0.12151	1.03159	0.49331	1.024515	0.70189	1.12563	0.088957
11.8	13.04	gi|56699409	RNA-binding motif protein, X chromosome isoform 1 [Homo sapiens]	6	0.92096	0.49133	0.87494	0.158879	0.877598	0.12292	1.0088	0.9293	0.966318	0.74655	0.87719	0.094431
14.68	48.24	gi|5803225	14-3-3 protein epsilon [Homo sapiens]	16	1.02601	0.76029	0.91136	0.328953	1.11497	0.15435	0.96656	0.78874	0.960346	0.44181	1.14985	0.097089
52.59	69.47	gi|50845388	annexin A2 isoform 1 [Homo sapiens]	52	1.03601	0.33861	0.89261	0.000799	0.952177	0.19231	1.09683	0.02102	0.986273	0.64679	0.93349	0.100108
7.85	53.99	gi|4885375	histone H1.2 [Homo sapiens]	16	0.87375	0.05937	0.7433	0.00219	0.542623	0.00103	0.73003	0.00159	0.947048	0.46755	0.75976	0.101682

9.2	24.44	gi|4506661	60S ribosomal protein L7a [Homo sapiens]	6	1.12561	0.37809	1.10248	0.529482	1.115835	0.16495	1.03566	0.61818	1.010542	0.93949	1.25406	0.104774
65.26	54.13	gi|16507237	78 kDa glucose-regulated protein precursor [Homo sapiens]	42	0.99068	0.80644	0.91924	0.037995	0.914667	0.10089	0.97414	0.72851	0.975383	0.64284	0.90792	0.107537
33.69	65.68	gi|21735621	malate dehydrogenase, mitochondrial precursor [Homo sapiens]	21	0.98306	0.72628	0.96187	0.550987	1.082983	0.19161	0.99879	0.98249	1.003288	0.95569	1.09901	0.108722
8.57	6.298	gi|4504763	integrin alpha-V isoform 1 precursor [Homo sapiens]	6	1.10731	0.34169	1.50407	0.12905	0.922235	0.67208	1.35084	0.4102	1.65825	0.06426	1.25584	0.110681
3.27	3.004	gi|38327625	citrate synthase, mitochondrial precursor [Homo sapiens]	2	0.90179	0.42891	1.04244	0.800114	0.948916	0.85177	1.10885	0.48796	0.896501	0.31286	1.30983	0.112072
2.03	1.04	gi|4505541	general vesicular transport factor p115 [Homo sapiens]	1	1.03	0.9444	1.16908	0.359291	1.468988	0.16016	1.34375	0.59047	1.403052	0.25749	1.86084	0.114754
11.64	4.447	gi|223029410	talin-1 [Homo sapiens]	7	0.7748	0.08765	1.01838	0.916424	0.810045	0.62668	0.53988	0.04862	0.830302	0.16782	0.80491	0.115327
15.01	30.56	gi|24431933	reticulon-4 isoform B [Homo sapiens]	9	1.03261	0.79202	0.92584	0.443218	0.86613	0.3369	0.98962	0.93193	0.999494	0.99403	0.75832	0.118577
2.22	40.45	gi|4502389	barrier-to-autointegration factor [Homo sapiens]	3	1.01481	0.90604	0.79065	0.497285	0.785352	0.47056	0.77148	0.48763	1.065285	0.63782	0.60144	0.122476
8.24	21.8	gi|15431297	60S ribosomal protein L13 isoform 1 [Homo sapiens]	6	1.00852	0.94051	1.04466	0.512794	0.999406	0.99678	0.99211	0.96695	1.008986	0.93835	0.85176	0.122519
6	11.89	gi|169404009	translocon-associated protein subunit alpha precursor [Homo sapiens]	3	0.95232	0.61818	0.87112	0.397806	0.856702	0.23079	1.06776	0.59316	0.959057	0.73145	0.80544	0.122538

4.09	37.5	gi|51036603	guanine nucleotide-binding protein G(I)/G(S)/G(O) subunit gamma-12 precursor [Homo sapiens]	3	0.82618	0.40915	0.66391	0.07069	0.823514	0.43842	0.91099	0.71981	0.765134	0.40552	0.64208	0.124749
19.89	60.61	gi|10863927	peptidyl-prolyl cis-trans isomerase A [Homo sapiens]	21	0.98345	0.72502	1.04141	0.522254	1.157293	0.14108	1.04379	0.67672	1.040531	0.7547	1.13552	0.125378
14.61	62.86	gi|4826898	profilin-1 [Homo sapiens]	11	0.93252	0.25277	0.9354	0.272724	1.1113	0.10339	0.95098	0.4023	0.930043	0.40442	1.13055	0.127681
13.22	18.74	gi|20127408	trifunctional enzyme subunit alpha, mitochondrial precursor [Homo sapiens]	9	0.93017	0.43003	0.88367	0.537465	0.804996	0.40295	1.0888	0.67464	0.886635	0.51654	0.85087	0.130116
12.73	20	gi|24797067	HLA class I histocompatibility antigen, A-1 alpha chain precursor [Homo sapiens]	8	0.88901	0.29354	0.94676	0.494894	0.929581	0.37731	0.97793	0.82815	0.935571	0.41568	0.85741	0.130658
6.37	14.08	gi|7019415	protein transport protein Sec61 subunit alpha isoform 1 [Homo sapiens]	5	0.79339	0.19075	0.97595	0.796917	0.744264	0.37933	0.62065	0.1723	0.702107	0.27466	0.70581	0.136075
8.35	6.289	gi|68509926	putative pre-mRNA-splicing factor ATP-dependent RNA helicase DHX15 [Homo sapiens]	5	0.62423	0.18212	0.47583	0.334467	1.130629	0.43257	0.77012	0.46447	0.811996	0.64092	0.49002	0.13646
2.12	4.664	gi|38201710	probable ATP-dependent RNA helicase DDX17 isoform 1 [Homo sapiens]	3	0.79915	0.61738	0.91094	0.620215	0.750311	0.21345	0.80939	0.28139	0.780581	0.24321	0.63829	0.138851

4.2	5.383	gi|4757732	apoptosis-inducing factor 1, mitochondrial isoform 1 precursor [Homo sapiens]	2	1.38271	0.07172	1.19782	0.407886	1.088847	0.41412	1.28989	0.41554	1.162864	0.38478	1.34436	0.140307
10.12	24.62	gi|238776833	THO complex subunit 4 [Homo sapiens]	6	0.83702	0.04946	0.94848	0.403375	0.86749	0.31273	0.87675	0.42273	0.897684	0.40794	0.88435	0.14495
12.3	43.94	gi|32189392	peroxiredoxin-2 isoform a [Homo sapiens]	10	1.34013	0.09637	1.27568	0.14954	1.190778	0.02567	1.19692	0.26779	1.15698	0.45387	1.28334	0.145991
1.35	2.03	gi|124494254	proliferation-associated protein 2G4 [Homo sapiens]	1	0.96538	0.89731	1.35889	0.039772	1.239406	0.2757	0.98931	0.97425	1.219182	0.19368	1.14722	0.146018
4.09	14.1	gi|19923437	GTP:AMP phosphotransferase, mitochondrial isoform a [Homo sapiens]	3	1.39625	0.08317	1.46051	0.046626	1.432712	0.05166	1.27401	0.37104	1.138681	0.44	1.42747	0.148779
20.35	55.61	gi|21361091	ubiquitin carboxyl-terminal hydrolase isozyme L1 [Homo sapiens]	12	0.94536	0.55362	1.07884	0.312914	1.099702	0.14413	0.92686	0.5639	0.969563	0.73363	1.14064	0.149277
5.99	11.48	gi|14141159	heterogeneous nuclear ribonucleoprotein H3 isoform b [Homo sapiens]	5	0.8342	0.1545	0.96127	0.711873	0.817383	0.18652	0.86804	0.22181	0.778232	0.09066	0.71379	0.153676
56.1	51.55	gi|5729877	heat shock cognate 71 kDa protein isoform 1 [Homo sapiens]	39	1.02418	0.63104	0.9707	0.403987	1.061664	0.19288	0.9861	0.86555	0.965753	0.41121	1.06446	0.153957
10.03	23.65	gi|4557032	L-lactate dehydrogenase B chain [Homo sapiens]	6	0.93851	0.60795	1.08169	0.304175	1.185435	0.06437	0.88422	0.54126	1.059711	0.73119	1.12483	0.154941

5.92	32	gi|48255933	non-histone chromosomal protein HMG-14 [Homo sapiens]	3	0.91603	0.6829	0.98843	0.959112	0.811181	0.18646	0.82704	0.40778	0.919272	0.33885	0.87101	0.160101
13	45.56	gi|4507651	tropomyosin alpha-4 chain isoform 2 [Homo sapiens]	13	0.9197	0.35893	0.94139	0.238868	0.978656	0.86419	0.77351	0.09697	0.898614	0.18298	0.88635	0.160554
2.03	11.8	gi|15451856	caveolin-1 isoform alpha [Homo sapiens]	1	0.99581	0.97294	0.88967	0.446766	0.866877	0.38527	0.89103	0.45106	0.978481	0.86208	0.68415	0.162168
7.77	28.87	gi|4506761	protein S100-A10 [Homo sapiens]	6	1.05949	0.39457	0.98038	0.763376	0.915157	0.21142	0.9942	0.92867	1.068271	0.33669	0.83821	0.162865
10.9	34.94	gi|94721252	vesicle-associated membrane protein-associated protein A isoform 2 [Homo sapiens]	8	1.227	0.01851	0.97704	0.71228	1.185619	0.34812	1.26298	0.03566	1.170574	0.09287	1.22005	0.164682
8.4	33.14	gi|4507669	translationally-controlled tumor protein [Homo sapiens]	6	1.08576	0.52472	1.09398	0.508183	1.116665	0.46569	1.13494	0.37263	1.084082	0.68352	1.23937	0.170834
2.02	10.25	gi|25188179	voltage-dependent anion-selective channel protein 3 isoform 1 [Homo sapiens]	3	1.02508	0.91757	1.16013	0.376609	1.484604	0.36729	1.26373	0.25659	0.81173	0.69302	1.54434	0.177514
2.17	10.84	gi|17158044	40S ribosomal protein S6 [Homo sapiens]	2	0.95076	0.6196	0.82852	0.091434	0.738315	0.04109	0.53979	0.06305	0.69574	0.1722	0.71755	0.1779
21.82	4.09	gi|33350932	cytoplasmic dynein 1 heavy chain 1 [Homo sapiens]	13	1.57248	0.29762	1.57923	0.217808	1.282865	0.35371	1.80211	0.13401	1.017224	0.96817	1.5064	0.181445
1.53	7.064	gi|50592988	cytochrome b-c1 complex subunit 2, mitochondrial precursor [Homo sapiens]	2	0.81533	0.4452	0.69962	0.31094	0.740066	0.414	0.68865	0.20942	0.423321	0.20012	0.70978	0.181631

9.16	12.75	gi|24307939	T-complex protein 1 subunit epsilon [Homo sapiens]	6	0.88391	0.83878	1.03728	0.930669	1.416377	0.6002	1.44063	0.48371	1.130974	0.78207	0.69751	0.182052
25.85	24.2	gi|19923142	importin subunit beta-1 [Homo sapiens]	17	1.05472	0.72744	1.03584	0.67655	1.133016	0.19697	1.16673	0.15872	0.998602	0.98495	1.13234	0.185815
6.49	17.1	gi|38372925	basigin isoform 2 [Homo sapiens]	4	1.01305	0.85925	1.00488	0.960318	0.988465	0.91265	0.947	0.49923	1.10776	0.58434	0.89814	0.189596
2.12	14.29	gi|78214522	60S ribosomal protein L38 [Homo sapiens]	1	0.96672	0.84305	0.91545	0.719896	0.828098	0.3865	0.66688	0.42456	1.096426	0.88091	0.40804	0.191292
3.66	11.28	gi|5729875	membrane-associated progesterone receptor component 1 [Homo sapiens]	3	1.29932	0.22776	1.19393	0.255245	0.922448	0.82759	1.2013	0.35792	1.273844	0.11659	1.30413	0.191907
1.96	2.809	gi|5803023	vesicular integral-membrane protein VIP36 precursor [Homo sapiens]	1	1.25437	0.44629	0.9238	0.572269	1.003053	0.9813	1.24652	0.57488	0.725378	0.19242	0.72577	0.192623
19.62	28.1	gi|5032093	neutral amino acid transporter B(0) isoform 1 [Homo sapiens]	14	0.99773	0.98539	0.97139	0.597192	0.993195	0.94792	1.06033	0.30024	1.012247	0.93042	0.90298	0.192785
9.28	12.15	gi|7657581	calcium-binding mitochondrial carrier protein Aralar2 isoform 2 [Homo sapiens]	5	1.24309	0.38294	1.04897	0.552228	1.137596	0.35039	1.21757	0.22643	1.018496	0.82502	0.77457	0.197527
18.48	34.01	gi|194248072	heat shock 70 kDa protein 1A/1B [Homo sapiens]	20	1.00579	0.94158	1.09057	0.121139	1.049526	0.65628	1.07026	0.41274	0.957757	0.53605	1.1032	0.200776
11.01	27.06	gi|11321585	guanine nucleotide-binding protein G(I)/G(S)/G(T) subunit beta-1 [Homo sapiens]	7	0.73007	0.19568	0.64127	0.141431	0.681516	0.19847	0.71464	0.34485	1.037866	0.77339	0.74014	0.204888

11.47	43.36	gi|4885381	histone H1.5 [Homo sapiens]	15	0.83443	0.01052	0.79034	0.005847	0.651536	0.00034	0.77306	0.05702	0.897578	0.16378	0.84588	0.205013
16.9	14.2	gi|156523968	poly [ADP-ribose] polymerase 1 [Homo sapiens]	10	1.21558	0.14288	1.17066	0.120212	1.051162	0.47741	1.0265	0.87789	1.061355	0.76352	1.172	0.207123
3.04	3.187	gi|77404397	staphylococcal nuclease domain-containing protein 1 [Homo sapiens]	3	1.17405	0.49812	1.22773	0.468987	1.488215	0.12081	1.08531	0.69621	1.110144	0.54598	1.36789	0.210295
4.76	5.495	gi|4503583	epoxide hydrolase 1 precursor [Homo sapiens]	2	0.84869	0.29741	0.72711	0.197375	1.081442	0.45904	0.87687	0.24494	0.884422	0.60119	0.86167	0.213117
8.71	24.41	gi|9845502	40S ribosomal protein SA [Homo sapiens]	6	1.03724	0.74771	1.08735	0.352101	1.152088	0.1949	1.2666	0.02453	0.950464	0.75669	1.16312	0.215801
4.14	24.35	gi|4506631	60S ribosomal protein L30 [Homo sapiens]	2	1.14158	0.69687	1.10043	0.803557	1.054163	0.80851	1.05796	0.56652	1.038772	0.81646	1.12587	0.218553
8.3	22.18	gi|11559923	eukaryotic translation initiation factor 4H isoform 1 [Homo sapiens]	5	1.12264	0.68798	1.25954	0.340565	1.042103	0.8514	1.01228	0.88499	1.066275	0.614	1.09901	0.225424
8.28	13.87	gi|40068518	6-phosphogluconate dehydrogenase, decarboxylating [Homo sapiens]	5	0.81545	0.14339	0.94935	0.623335	1.02256	0.78535	0.79003	0.11995	0.8653	0.35679	0.77777	0.227224
2.11	3.371	gi|24797106	FAS-associated factor 2 [Homo sapiens]	1	1.06334	0.65017	1.45771	0.417112	1.365147	0.33565	1.01105	0.94592	1.360478	0.20061	1.30254	0.231693
2.58	9.594	gi|5453549	peroxiredoxin-4 precursor [Homo sapiens]	3	1.09696	0.59119	1.16346	0.599874	1.087432	0.78277	1.19647	0.61772	1.284313	0.55549	1.31451	0.235158
3.24	7.754	gi|23397429	eukaryotic translation initiation factor 3 subunit M [Homo sapiens]	2	0.89871	0.47538	1.31111	0.222737	1.013742	0.93117	0.81456	0.2861	1.138485	0.56671	1.28961	0.236077

2.02	4.537	gi|162329583	cleavage and polyadenylation specificity factor subunit 6 [Homo sapiens]	1	0.90214	0.50643	1.11391	0.867037	1.458042	0.54873	1.06447	0.65983	1.296901	0.68157	1.30871	0.236755
18.46	13.39	gi|237681109	sodium/potassium-transporting ATPase subunit alpha-1 isoform c [Homo sapiens]	18	1.02552	0.80461	1.00024	0.998588	0.844812	0.12679	0.91605	0.50142	0.914821	0.38021	0.91843	0.239598
8.82	21.58	gi|14251209	chloride intracellular channel protein 1 [Homo sapiens]	6	0.90088	0.30114	0.95147	0.772095	1.09574	0.46797	0.96541	0.77434	0.984818	0.91828	0.88861	0.24316
4.53	6.239	gi|109389365	glucose-6-phosphate 1-dehydrogenase isoform a [Homo sapiens]	2	1.20426	0.33865	1.43066	0.189014	1.624618	0.2636	1.55257	0.22793	1.448891	0.23758	1.30435	0.249744
6.35	24.44	gi|7657532	protein S100-A6 [Homo sapiens]	3	0.98566	0.85809	1.09844	0.296211	1.059034	0.49517	1.03444	0.67966	1.031681	0.7023	1.1115	0.250352
21.3	42.45	gi|5803227	14-3-3 protein theta [Homo sapiens]	17	0.97905	0.87629	1.0435	0.605723	0.966232	0.87085	0.92408	0.61253	1.178148	0.16	1.1743	0.251827
2	8.744	gi|88953571	PREDICTED: POTE ankyrin domain family member I isoform 2 [Homo sapiens]	34	0.86459	0.70652	0.90932	0.524006	0.832067	0.32213	0.85508	0.3682	0.725671	0.19692	0.78485	0.253517
32.32	35.84	gi|5031877	lamin-B1 isoform 1 [Homo sapiens]	18	0.94349	0.56293	0.90136	0.275431	0.918949	0.44907	0.93501	0.4858	0.893712	0.32872	0.89476	0.254337
5.39	11.56	gi|4501853	3-ketoacyl-CoA thiolase, peroxisomal isoform a [Homo sapiens]	3	2.16834	0.1858	1.18441	0.641874	1.62163	0.13731	1.42343	0.17432	1.2076	0.45654	1.25897	0.259562
4.3	9.246	gi|7661920	eukaryotic initiation factor 4A-III [Homo sapiens]	2	1.29956	0.34769	1.01607	0.902547	1.212463	0.31844	1.27806	0.42026	0.783348	0.76584	0.79002	0.262826
50.42	31.55	gi|55956788	nucleolin [Homo sapiens]	29	1.05759	0.23708	1.02976	0.572142	0.98183	0.74371	1.09084	0.15784	1.007521	0.87671	1.05735	0.263211

2.95	3.655	gi|308818200	dihydropyrimidinase-related protein 3 isoform 1 [Homo sapiens]	2	1.34309	0.06857	1.2031	0.161372	0.967412	0.85001	1.52203	0.03584	1.210626	0.5645	1.23319	0.263459
24.27	32.08	gi|23308577	D-3-phosphoglycerate dehydrogenase [Homo sapiens]	17	1.06596	0.47514	0.97881	0.832898	1.203403	0.08204	1.02448	0.76084	1.029579	0.60888	1.15234	0.267369
25.86	13.07	gi|4758012	clathrin heavy chain 1 [Homo sapiens]	16	0.99052	0.92065	0.94667	0.522496	1.13722	0.24734	0.88892	0.13223	0.916398	0.48145	1.09911	0.268588
2.16	30.23	gi|4502985	cytochrome c oxidase subunit 6B1 [Homo sapiens]	2	0.90839	0.35899	0.88557	0.684512	0.735976	0.10006	0.76604	0.22395	0.772901	0.17148	0.81212	0.274825
31.87	8.225	gi|153945728	microtubule-associated protein 1B [Homo sapiens]	17	0.94326	0.58955	0.9836	0.825151	0.952414	0.62219	0.87584	0.26177	0.900398	0.23709	0.88394	0.275407
85.56	84.33	gi|62414289	vimentin [Homo sapiens]	111	0.96853	0.22969	0.8979	0.00024	0.966613	0.26142	0.99839	0.96544	0.992586	0.77803	0.96001	0.276668
85.56	84.33	gi|62414289	vimentin [Homo sapiens]	111	0.96853	0.22969	0.8979	0.00024	0.966613	0.26142	0.99839	0.96544	0.992586	0.77803	0.96001	0.276668
6.4	27.31	gi|5453555	GTP-binding nuclear protein Ran [Homo sapiens]	5	0.98859	0.87907	1.14585	0.42636	1.194501	0.25584	1.03131	0.69113	0.94485	0.71941	1.14543	0.276881
6.52	14.19	gi|4507171	SPARC precursor [Homo sapiens]	3	0.9158	0.47886	1.02166	0.864754	0.936253	0.51281	0.94402	0.83367	0.974326	0.78471	0.73391	0.27843
32.05	53.69	gi|32189394	ATP synthase subunit beta, mitochondrial precursor [Homo sapiens]	26	1.06947	0.33782	1.02526	0.810252	1.085629	0.19352	1.06683	0.36414	1.062145	0.36963	1.05613	0.282311
15.14	32.7	gi|4502551	calumenin isoform a precursor [Homo sapiens]	11	1.0541	0.35248	0.92044	0.374923	1.046819	0.65407	1.02137	0.84663	1.013005	0.8751	0.90294	0.28484
30.99	46.28	gi|4757900	calreticulin precursor [Homo sapiens]	22	0.97558	0.48559	0.9151	0.110982	0.924536	0.15201	1.02055	0.6353	1.000256	0.99424	0.94295	0.285087

3.78	13.38	gi|4506619	60S ribosomal protein L24 [Homo sapiens]	2	0.88742	0.57195	0.87413	0.254582	0.97148	0.78669	0.90032	0.34225	0.858709	0.57737	0.81159	0.285288
4.33	12.64	gi|19557691	surfeit locus protein 4 [Homo sapiens]	3	1.07383	0.75128	0.79406	0.111418	0.815363	0.65159	1.00065	0.99896	0.826807	0.64556	0.68096	0.286924
14.4	25.05	gi|34147630	elongation factor Tu, mitochondrial precursor [Homo sapiens]	8	1.24054	0.2593	1.00401	0.978324	1.262414	0.32398	1.23422	0.17278	1.179966	0.2319	1.20203	0.287698
4.03	20	gi|262118227	cytochrome c oxidase subunit 7A2, mitochondrial precursor [Homo sapiens]	2	1.1199	0.72902	0.90201	0.486512	0.756671	0.2722	1.38307	0.18831	0.946541	0.67701	1.2207	0.292914
9.55	32.21	gi|5901912	calmodulin [Homo sapiens]	4	0.97733	0.85572	1.0233	0.851035	1.04108	0.74264	0.91374	0.49865	1.008276	0.91537	0.83326	0.292997
6.37	5.497	gi|157694492	myb-binding protein 1A isoform 2 [Homo sapiens]	5	1.00363	0.98643	0.9421	0.906806	0.905539	0.52855	1.10364	0.52725	0.925595	0.78667	0.80372	0.296452
4.38	38.46	gi|4504253	histone H2A.x [Homo sapiens]	55	1.02226	0.91213	0.96013	0.770314	0.821872	0.25679	1.0363	0.92006	0.957424	0.76937	0.90772	0.304709
8.06	17.58	gi|4506003	serine/threonine-protein phosphatase PP1-alpha catalytic subunit isoform 1 [Homo sapiens]	6	1.48714	0.13004	1.10011	0.713846	1.277277	0.52397	1.33377	0.25545	1.172138	0.4535	1.28387	0.30728
11.93	21.53	gi|42794771	thioredoxin domain-containing protein 5 isoform 1 precursor [Homo sapiens]	7	1.14981	0.0688	1.00059	0.996582	1.070191	0.40696	0.96876	0.79451	0.947137	0.80295	1.09618	0.309544
2.19	4.61	gi|4504619	insulin-like growth factor-binding protein 7 isoform 1 precursor [Homo sapiens]	1	0.84172	0.29423	0.98065	0.834212	1.054621	0.66737	1.60677	0.49731	1.126455	0.67446	0.83734	0.310771

4.4	7.745	gi|4557809	ornithine aminotransferase, mitochondrial isoform 1 [Homo sapiens]	6	1.24849	0.12014	1.16548	0.620637	1.100635	0.88714	0.97603	0.91645	1.240592	0.14432	1.12319	0.31102
5.62	16.48	gi|5803013	endoplasmic reticulum resident protein 29 isoform 1 precursor [Homo sapiens]	4	0.84553	0.25641	1.10238	0.275949	1.055268	0.87182	0.88294	0.27119	0.94896	0.7498	1.17577	0.31394
6.07	16.79	gi|4505415	NAD(P)H dehydrogenase [quinone] 1 isoform a [Homo sapiens]	5	1.36635	0.37502	1.01123	0.930016	1.33663	0.34932	1.45685	0.29703	1.027479	0.83314	1.57378	0.314236
2.22	7.487	gi|4505621	phosphatidylethanolamine-binding protein 1 preproprotein [Homo sapiens]	1	0.96277	0.8957	1.09319	0.533952	0.903624	0.4929	1.02145	0.91865	1.219476	0.35161	1.19588	0.321973
2.68	4.412	gi|358356404	60S ribosomal protein L15 isoform 1 [Homo sapiens]	1	0.87189	0.36029	0.93705	0.674173	1.013222	0.93016	1.11807	0.32778	1.005237	0.93799	0.93831	0.322112
9.7	31.86	gi|13904870	40S ribosomal protein S5 [Homo sapiens]	7	0.9506	0.72658	1.0316	0.603441	0.924649	0.46719	0.99064	0.95691	1.001791	0.98677	1.091	0.322428
2.2	48.28	gi|4501881	actin, alpha skeletal muscle [Homo sapiens]	84	0.70402	0.19171	0.88213	0.452415	0.895226	0.49113	0.9076	0.68294	0.910421	0.74174	0.83025	0.335221
20.35	61.46	gi|4504517	heat shock protein beta-1 [Homo sapiens]	14	1.03033	0.75301	1.09164	0.398213	1.129264	0.45398	1.07204	0.36605	1.008009	0.93325	1.14863	0.335319
3	15.63	gi|23308579	prostaglandin E synthase 3 [Homo sapiens]	2	1.09274	0.60681	1.33151	0.112159	1.200575	0.47968	1.17833	0.45949	1.215746	0.48534	1.12312	0.341192
37.36	23.31	gi|100913206	ATP-dependent RNA helicase A [Homo sapiens]	25	1.01038	0.85557	0.98092	0.810188	1.026626	0.71249	0.99051	0.9034	0.908446	0.21786	0.93231	0.343017

6	15.03	gi|5453597	F-actin-capping protein subunit alpha-1 [Homo sapiens]	3	1.08743	0.8451	1.19702	0.574836	1.148574	0.63926	0.8406	0.67537	1.190998	0.50429	1.17969	0.344035
1.67	8.013	gi|7705855	estradiol 17-beta-dehydrogenase 12 [Homo sapiens]	2	1.35665	0.14963	1.58221	0.299346	1.327157	0.60819	0.82147	0.54246	2.201652	0.16227	1.17915	0.348877
32.68	55.25	gi|4885379	histone H1.4 [Homo sapiens]	18	0.86188	0.15805	0.79458	0.062715	0.581451	0.00631	0.783	0.05277	0.955935	0.61036	0.78812	0.349009
20.58	32.02	gi|4503529	eukaryotic initiation factor 4A-I isoform 1 [Homo sapiens]	13	1.04269	0.74889	1.03994	0.678853	1.271338	0.05202	1.0321	0.8665	0.954919	0.82177	1.16915	0.351373
4.88	14.38	gi|4506691	40S ribosomal protein S16 [Homo sapiens]	2	0.97562	0.80498	1.08435	0.534558	0.91199	0.46498	0.90795	0.25085	1.061548	0.63913	1.08996	0.352086
5.35	21.47	gi|89903012	cell division control protein 42 homolog isoform 1 [Homo sapiens]	4	0.84946	0.37036	0.96503	0.661392	0.873435	0.40147	0.85893	0.56613	0.93952	0.65745	0.8919	0.363234
4.28	8.456	gi|4826659	F-actin-capping protein subunit beta isoform 1 [Homo sapiens]	2	0.67802	0.35754	0.69959	0.172607	0.851274	0.65389	0.86143	0.37487	0.66671	0.15342	0.85952	0.369847
16.25	8.025	gi|7669550	vinculin isoform meta-VCL [Homo sapiens]	7	0.88768	0.17609	0.86082	0.075983	0.892825	0.45546	0.90485	0.43053	0.975215	0.86887	0.89204	0.37485
16.66	23.14	gi|35493916	dolichyl-diphosphooligosaccharide--protein glycosyltransferase subunit 2 isoform 1 precursor [Homo sapiens]	16	0.97184	0.85688	0.82452	0.080194	0.845658	0.09154	0.94712	0.71073	0.862415	0.34484	0.82828	0.378348
14	22.43	gi|4758112	spliceosome RNA helicase DDX39B [Homo sapiens]	11	0.76734	0.20451	1.11319	0.323403	0.756887	0.24072	1.25882	0.36395	0.926787	0.45626	0.75424	0.37859
11.59	15.71	gi|7549809	plastin-3 isoform 1 [Homo sapiens]	9	1.12989	0.64507	0.90527	0.733379	1.317888	0.17397	0.86056	0.69041	0.96376	0.83803	1.20141	0.379589

14.51	18.85	gi|21361399	serine/threonine-protein phosphatase 2A 65 kDa regulatory subunit A alpha isoform [Homo sapiens]	9	0.85201	0.64513	0.93269	0.807974	1.291011	0.53085	1.19777	0.7306	1.030423	0.92072	1.31892	0.380861
11.33	36.02	gi|4758504	3-hydroxyacyl-CoA dehydrogenase type-2 isoform 1 [Homo sapiens]	5	1.16862	0.44494	1.33119	0.096689	1.177838	0.49	1.0947	0.79475	1.573738	0.01477	1.13974	0.381154
3.4	19.47	gi|4503253	dolichyl-diphosphooligosaccharide--protein glycosyltransferase subunit DAD1 [Homo sapiens]	2	0.97294	0.92271	1.26859	0.287512	1.130249	0.50102	1.09105	0.81061	0.985191	0.90638	1.15704	0.386139
3.13	8.871	gi|5902076	serine/arginine-rich splicing factor 1 isoform 1 [Homo sapiens]	2	0.80818	0.51448	0.95702	0.88235	0.88861	0.73781	0.93516	0.86581	0.90713	0.71945	0.74119	0.387206
4.11	4.595	gi|4826686	ATP-dependent RNA helicase DDX1 [Homo sapiens]	2	0.96152	0.7616	0.50291	0.169483	0.979637	0.92474	1.28961	0.57091	0.61504	0.12877	0.86852	0.391631
10.29	11.88	gi|55956899	keratin, type I cytoskeletal 9 [Homo sapiens]	6	0.859	0.1927	0.99136	0.910421	1.174134	0.18032	1.15622	0.23457	0.965199	0.70957	1.10427	0.395095
5.06	24	gi|4506633	60S ribosomal protein L31 isoform 1 [Homo sapiens]	3	1.23708	0.47402	0.95535	0.724579	1.41888	0.35286	0.98525	0.96622	1.213987	0.63144	1.1551	0.397981
2.36	4.981	gi|5453710	LIM and SH3 domain protein 1 [Homo sapiens]	1	0.7038	0.17472	0.74995	0.303498	0.81862	0.29299	1.06878	0.74451	0.721986	0.50264	0.74921	0.401414
7.5	21.59	gi|4506723	40S ribosomal protein S3a isoform 1 [Homo sapiens]	6	1.12182	0.58683	1.07692	0.62046	1.139729	0.42901	1.17579	0.43977	1.065154	0.63398	1.10216	0.401429

25.7	38.78	gi|4506243	polypyrimidine tract-binding protein 1 isoform a [Homo sapiens]	18	1.06292	0.50756	1.06923	0.515736	1.03194	0.7921	1.05954	0.60728	1.071681	0.34712	1.09726	0.405096
9.37	13.6	gi|4503249	protein DEK isoform 1 [Homo sapiens]	6	0.90486	0.7789	0.82548	0.362847	0.887533	0.5517	0.78952	0.49732	0.937237	0.82238	0.72629	0.405633
14.64	25.24	gi|213511508	B-cell receptor-associated protein 31 isoform a [Homo sapiens]	10	0.9788	0.86518	0.94279	0.594634	0.912101	0.48864	1.007	0.95315	1.037329	0.68442	0.92084	0.409043
7.42	23.37	gi|219555707	eukaryotic translation initiation factor 5A-1 isoform A [Homo sapiens]	12	0.93983	0.54649	0.9442	0.764423	0.988686	0.90673	0.86285	0.22899	0.906192	0.37119	0.80789	0.411159
1.55	4.286	gi|151101292	thioredoxin-related transmembrane protein 1 precursor [Homo sapiens]	1	1.06382	0.75255	0.97657	0.857522	0.819195	0.30702	0.88399	0.46597	0.94309	0.88688	0.87165	0.412176
2.73	1.905	gi|65506891	4F2 cell-surface antigen heavy chain isoform c [Homo sapiens]	1	0.98317	0.93864	0.84624	0.244296	1.115642	0.53319	0.93078	0.55737	1.007492	0.96461	1.15408	0.413802
6.9	12.29	gi|4504897	importin subunit alpha-2 [Homo sapiens]	4	1.62496	0.16839	1.58207	0.135682	1.24339	0.36447	1.51981	0.50696	1.039731	0.89515	1.5101	0.415434
27.47	68.18	gi|226529917	triosephosphate isomerase isoform 2 [Homo sapiens]	18	0.89284	0.09459	0.9824	0.725009	1.039563	0.50875	0.86402	0.04131	0.996977	0.94683	1.0504	0.4184
26.12	45.92	gi|10835063	nucleophosmin isoform 1 [Homo sapiens]	27	1.05093	0.20853	0.98486	0.687201	0.932746	0.16808	1.0139	0.77562	0.993499	0.88674	0.96765	0.418435
19.75	52.68	gi|4506667	60S acidic ribosomal protein P0 [Homo sapiens]	10	1.07767	0.28966	0.92404	0.551593	0.973779	0.83745	1.06728	0.4878	1.123923	0.35376	1.08972	0.4209
9.86	54.29	gi|11128019	cytochrome c [Homo sapiens]	7	1.02581	0.90995	1.1736	0.485643	1.168768	0.23072	1.206	0.51323	1.136284	0.41989	1.14466	0.422321

1.67	3.499	gi|5803181	stress-induced-phosphoprotein 1 [Homo sapiens]	1	0.71762	0.41633	0.77017	0.230487	0.873029	0.40131	0.75715	0.48774	0.672277	0.46885	1.13446	0.423418
6.23	5.735	gi|149999606	mannosyl-oligosaccharide glucosidase isoform 1 [Homo sapiens]	4	1.08059	0.53739	1.14425	0.238759	1.1575	0.39612	1.20626	0.22942	1.027046	0.82377	1.22927	0.424115
2.06	1.744	gi|13375618	delta(24)-sterol reductase precursor [Homo sapiens]	1	0.96975	0.95351	1.02975	0.912942	0.726643	0.58232	1.01279	0.96941	0.809533	0.46642	0.69083	0.428542
4.59	33.59	gi|6912616	histone H2A.V isoform 1 [Homo sapiens]	9	0.95702	0.75379	0.85738	0.31234	0.799618	0.39941	0.95914	0.85394	0.973601	0.77491	0.91318	0.430038
12.27	17.53	gi|47132620	keratin, type II cytoskeletal 2 epidermal [Homo sapiens]	10	0.94036	0.69134	1.17098	0.215737	0.949491	0.52853	1.22133	0.14278	1.251278	0.05142	1.10158	0.431077
29.14	48.39	gi|24119203	tropomyosin alpha-3 chain isoform 2 [Homo sapiens]	17	1.09868	0.52294	1.13547	0.264856	1.02411	0.77098	1.07533	0.58977	1.025008	0.85158	1.10599	0.4331
4.54	1.504	gi|7305053	myoferlin isoform a [Homo sapiens]	3	1.03642	0.91549	1.07562	0.753873	0.677725	0.15841	1.17281	0.35344	0.804299	0.27121	0.80158	0.434082
5.17	11.54	gi|71772415	40S ribosomal protein S15a [Homo sapiens]	2	1.08261	0.30057	0.86993	0.526937	1.005628	0.96992	1.06918	0.64765	0.895366	0.53786	0.9438	0.435165
19.01	71.69	gi|5031635	cofilin-1 [Homo sapiens]	13	0.98335	0.79485	1.06505	0.609521	1.073792	0.50449	0.94654	0.66373	1.006219	0.93333	1.08085	0.437716
4.87	17.72	gi|5031741	dnaJ homolog subfamily A member 2 [Homo sapiens]	4	1.02126	0.95399	0.95642	0.65181	0.777159	0.21533	1.08462	0.83024	1.354666	0.12297	1.18166	0.440861
3.65	23.53	gi|20357529	guanine nucleotide-binding protein G(I)/G(S)/G(T) subunit beta-2 [Homo sapiens]	5	1.12035	0.48505	1.20046	0.396539	1.116905	0.28669	0.87631	0.53161	1.27117	0.30243	1.06684	0.441087
3.09	9.509	gi|38201714	ELAV-like protein 1 [Homo sapiens]	3	1.41868	0.19346	1.17549	0.435049	1.144978	0.407	1.38872	0.18715	1.15082	0.57414	1.18829	0.441784

13.12	30.82	gi|148833484	poly(rC)-binding protein 2 isoform c [Homo sapiens]	10	0.8692	0.5888	0.79919	0.127357	1.023241	0.9611	0.89854	0.74636	0.88669	0.61133	0.80637	0.442107
60.22	77.33	gi|4501887	actin, cytoplasmic 2 [Homo sapiens]	122	1.04669	0.76057	0.9772	0.835104	1.156228	0.46372	1.12886	0.57932	0.952823	0.68722	1.13584	0.443001
64.06	45.78	gi|27436946	lamin isoform A [Homo sapiens]	33	1.00508	0.89186	0.97829	0.718695	0.922786	0.10936	0.99433	0.90623	0.986285	0.69962	0.95111	0.446249
4.04	20.35	gi|4502847	cold-inducible RNA-binding protein [Homo sapiens]	4	0.77481	0.23807	0.96341	0.8403	0.974154	0.9343	0.84858	0.47177	0.883339	0.43224	0.88827	0.446678
2.34	40	gi|15617199	histone H2A type 3 [Homo sapiens]	42	1.09822	0.74304	0.87151	0.415005	0.765686	0.38284	1.16096	0.38969	1.043543	0.75415	0.88391	0.448851
1.81	6.704	gi|7706495	dnaJ homolog subfamily B member 11 precursor [Homo sapiens]	2	0.93474	0.87702	0.53691	0.101191	0.806413	0.2753	0.5963	0.38777	0.827227	0.3501	0.79794	0.450096
6.16	25.6	gi|4506707	40S ribosomal protein S25 [Homo sapiens]	4	1.05992	0.71046	0.99801	0.984935	0.891834	0.22233	1.00881	0.92385	1.001775	0.9825	0.93387	0.453378
24.08	31.16	gi|195972866	keratin, type I cytoskeletal 10 [Homo sapiens]	18	0.99279	0.96579	1.17686	0.25241	1.082097	0.65185	1.1831	0.40404	1.176889	0.26041	1.15457	0.453813
1.82	8.824	gi|72534660	serine/arginine-rich splicing factor 7 isoform 1 [Homo sapiens]	2	1.01438	0.96006	1.32191	0.462731	1.03348	0.93519	0.9051	0.57763	1.129918	0.43756	1.25841	0.454052
3.37	3.239	gi|21361282	serine/arginine-rich splicing factor 4 [Homo sapiens]	2	0.86496	0.58839	0.9941	0.985146	0.989135	0.93152	0.69451	0.50631	0.947242	0.68143	0.83973	0.454583
9.39	24.34	gi|11968182	40S ribosomal protein S18 [Homo sapiens]	4	0.9108	0.17598	0.9615	0.565209	0.836623	0.29264	0.96303	0.84958	1.048824	0.83849	0.92731	0.46041
9.36	13.58	gi|62750354	matrin-3 isoform a [Homo sapiens]	11	1.04516	0.83428	1.19344	0.652837	1.008595	0.97584	0.94902	0.77475	1.150566	0.455	1.23023	0.463137
10.86	9.359	gi|71773106	AP-2 complex subunit beta isoform a [Homo sapiens]	6	1.03159	0.80628	0.90953	0.514151	0.860359	0.37066	0.88525	0.43526	0.974676	0.93431	0.80142	0.464299

5.49	20.15	gi|51599151	calpain small subunit 1 [Homo sapiens]	3	1.0993	0.90564	1.2801	0.713734	1.693094	0.48935	1.52327	0.58126	0.975995	0.97003	1.2446	0.464515
6.79	1.756	gi|91208426	pre-mRNA-processing-splicing factor 8 [Homo sapiens]	4	0.74541	0.52013	0.93731	0.762332	0.802356	0.39367	0.788	0.63705	0.875564	0.80852	0.82589	0.46519
2.35	8.029	gi|13904866	60S ribosomal protein L28 isoform 2 [Homo sapiens]	1	1.1852	0.61711	1.19004	0.350877	1.095949	0.54876	1.1129	0.49895	1.056091	0.72529	1.26634	0.469429
1.7	22.18	gi|4506663	60S ribosomal protein L8 [Homo sapiens]	3	1.34108	0.1674	1.18917	0.596438	0.934418	0.82729	1.33352	0.31409	1.327422	0.05463	1.3069	0.473156
59.02	46.15	gi|4503483	elongation factor 2 [Homo sapiens]	39	0.94928	0.36084	1.04053	0.380718	1.053971	0.35544	0.9786	0.75631	0.929768	0.18854	1.04154	0.473738
4.45	4.67	gi|197276600	microtubule-associated protein 4 isoform 4 [Homo sapiens]	4	1.27162	0.35766	1.30783	0.138875	0.971935	0.77141	1.24298	0.34491	1.142233	0.44348	1.17	0.47386
59.33	62.4	gi|20070125	protein disulfide-isomerase precursor [Homo sapiens]	36	0.9796	0.56599	0.92769	0.057465	0.928316	0.02639	0.96119	0.35188	1.007923	0.82127	0.97761	0.477783
8.82	44.44	gi|5031749	non-histone chromosomal protein HMG-17 [Homo sapiens]	7	1.02829	0.76222	0.94558	0.568522	0.847099	0.15126	0.96638	0.60275	0.990646	0.88526	0.95379	0.477923
8.82	44.44	gi|5031749	non-histone chromosomal protein HMG-17 [Homo sapiens]	7	1.02829	0.76222	0.94558	0.568522	0.847099	0.15126	0.96638	0.60275	0.990646	0.88526	0.95379	0.477923
6.93	9.854	gi|48762932	T-complex protein 1 subunit theta [Homo sapiens]	4	0.92715	0.48706	0.96602	0.82075	0.929325	0.69985	0.87594	0.5857	1.082928	0.61024	0.86331	0.478863
4.73	2.383	gi|54112121	splicing factor 3B subunit 3 [Homo sapiens]	3	0.81032	0.48085	0.98602	0.920135	1.05857	0.67167	0.5949	0.49536	1.079662	0.84245	0.89031	0.48195

4.05	7.161	gi|4758756	nucleosome assembly protein 1-like 1 [Homo sapiens]	2	1.062	0.82004	1.2756	0.571647	1.408068	0.46674	1.48367	0.24859	1.196134	0.33178	1.30001	0.484108
16.2	62.57	gi|5453740	myosin regulatory light chain 12A [Homo sapiens]	12	1.03618	0.53408	1.07574	0.396769	1.10096	0.25569	1.06196	0.4021	1.059293	0.3758	0.9605	0.484747
8.01	11.87	gi|373251164	glutaminase kidney isoform, mitochondrial isoform 2 [Homo sapiens]	6	0.74804	0.21256	0.77018	0.315563	0.807316	0.31161	0.7773	0.4977	0.717889	0.29766	0.84106	0.487298
2.85	1.876	gi|41393608	reticulon-3 isoform b [Homo sapiens]	2	0.80302	0.36014	0.86762	0.389095	0.664497	0.27998	0.77081	0.7731	0.900955	0.48418	0.7174	0.493207
2.9	8.914	gi|156564403	pyruvate dehydrogenase E1 component subunit beta, mitochondrial isoform 1 precursor [Homo sapiens]	2	1.45512	0.16682	1.33691	0.487042	1.523417	0.43403	1.45412	0.24868	1.41206	0.69537	1.298	0.49854
51.12	71.17	gi|29788785	tubulin beta chain [Homo sapiens]	51	0.90405	0.36942	0.91409	0.416252	1.033772	0.7416	0.9256	0.47261	0.917076	0.43044	1.07465	0.499031
8.08	8.385	gi|17402900	far upstream element-binding protein 1 [Homo sapiens]	6	1.03031	0.66506	1.10446	0.493208	0.912083	0.35392	1.08702	0.37794	1.001769	0.9928	1.14004	0.499632
15.54	14.91	gi|5453832	hypoxia up-regulated protein 1 precursor [Homo sapiens]	10	1.03014	0.81577	0.97909	0.8833	0.952214	0.64282	0.98593	0.92964	0.952431	0.60746	0.87514	0.500821
5.32	5.253	gi|66933016	inosine-5'-monophosphate dehydrogenase 2 [Homo sapiens]	4	1.03226	0.89524	1.09494	0.50848	1.268973	0.42403	1.3762	0.15979	1.245079	0.48519	1.19792	0.502222

2.63	5.122	gi|4504957	lysosome-associated membrane glycoprotein 2 isoform A precursor [Homo sapiens]	2	0.54855	0.55156	1.08974	0.849683	0.484158	0.57545	0.37086	0.39568	0.6991	0.54638	0.51917	0.506245
4.23	31.03	gi|4826848	NADH dehydrogenase [ubiquinone] 1 alpha subcomplex subunit 5 [Homo sapiens]	2	1.46828	0.41391	1.14203	0.815197	0.917116	0.54645	1.22387	0.73155	1.096227	0.88499	1.28746	0.506837
6.17	6.658	gi|4503131	catenin beta-1 [Homo sapiens]	3	0.41141	0.15644	0.66533	0.51162	0.503723	0.46341	0.41106	0.25461	0.599967	0.48447	0.41318	0.507001
63.94	40.4	gi|12025678	alpha-actinin-4 [Homo sapiens]	36	0.97761	0.81249	1.05219	0.64193	1.086704	0.45213	1.05716	0.61573	0.980235	0.82704	1.07302	0.511242
4.35	15.43	gi|332801090	heterogeneous nuclear ribonucleoprotein D-like isoform b [Homo sapiens]	6	1.00871	0.91679	1.05032	0.446338	0.913776	0.26441	0.98511	0.90239	1.054689	0.41219	0.89372	0.512474
74.02	44.33	gi|4507677	endoplasmin precursor [Homo sapiens]	42	0.9961	0.93353	0.94848	0.248495	0.968327	0.51543	0.9863	0.81623	0.976286	0.57443	0.96779	0.512923
1.57	4.478	gi|5803149	transmembrane emp24 domain-containing protein 2 precursor [Homo sapiens]	1	1.41839	0.40694	1.2308	0.287656	1.26119	0.2614	1.52491	0.1496	1.161351	0.65161	1.39668	0.513323
2.03	62.25	gi|5174735	tubulin beta-4B chain [Homo sapiens]	42	0.85065	0.43007	0.9246	0.575418	0.790037	0.41365	0.90389	0.49652	1.074749	0.66239	1.09878	0.518424
11.66	16.6	gi|19923653	PC4 and SFRS1-interacting protein isoform 2 [Homo sapiens]	8	1.17679	0.73143	1.56402	0.358592	0.968929	0.9434	1.46777	0.32185	1.363601	0.25718	1.28947	0.522585

5.85	10.4	gi|60279268	splicing factor U2AF 65 kDa subunit isoform b [Homo sapiens]	4	1.2201	0.66253	1.02211	0.952784	1.130686	0.83639	0.93141	0.83825	1.173532	0.5247	1.13973	0.52352
5.72	7.914	gi|112380628	lysosome-associated membrane glycoprotein 1 precursor [Homo sapiens]	3	0.92406	0.43426	1.07253	0.481096	0.862072	0.21099	0.78483	0.09717	0.937752	0.51208	0.78737	0.525828
17.81	20.36	gi|4758138	probable ATP-dependent RNA helicase DDX5 [Homo sapiens]	9	1.02008	0.93999	1.03103	0.885579	1.087795	0.60812	0.90254	0.59855	0.900662	0.697	0.85903	0.527072
2.84	15.89	gi|22208971	high mobility group protein HMG-I/HMG-Y isoform a [Homo sapiens]	2	1.16637	0.22879	1.11889	0.314787	0.942611	0.76233	0.96675	0.72794	1.098282	0.38348	1.06576	0.530562
2.52	2.347	gi|30581111	minor histocompatibility antigen H13 isoform 3 [Homo sapiens]	1	0.96889	0.88579	1.0853	0.672652	0.932619	0.85235	0.80742	0.27991	0.966258	0.79095	0.57797	0.530633
17.71	16.9	gi|5031755	heterogeneous nuclear ribonucleoprotein R isoform 2 [Homo sapiens]	12	1.1786	0.36141	1.03955	0.91766	1.411819	0.05493	1.20407	0.33353	1.512006	0.06272	1.34337	0.531745
2.08	3.639	gi|21361361	acetolactate synthase-like protein [Homo sapiens]	2	0.96311	0.95496	1.11329	0.773986	1.2958	0.74466	1.02802	0.94228	0.89748	0.78731	1.2972	0.533523
1.75	4.592	gi|4506609	60S ribosomal protein L19 [Homo sapiens]	1	1.28745	0.58224	1.12827	0.259487	1.136556	0.75982	1.21129	0.29878	1.386144	0.3114	1.23701	0.533725
24.42	42.35	gi|5453539	multifunctional protein ADE2 isoform 2 [Homo sapiens]	14	1.00356	0.9713	1.00013	0.998729	1.148299	0.30568	1.09789	0.39572	0.967332	0.82108	1.06801	0.541305

33.86	6.371	gi|126032350	DNA-dependent protein kinase catalytic subunit isoform 2 [Homo sapiens]	22	1.20563	0.32156	1.04779	0.533682	1.107957	0.44994	1.05744	0.66483	1.165061	0.4342	1.10473	0.543078
6.03	17.34	gi|4502969	catechol O-methyltransferase isoform MB-COMT [Homo sapiens]	4	0.93424	0.61858	0.80864	0.279603	0.927369	0.90939	1.13765	0.41947	0.713404	0.1827	0.77919	0.546947
7.11	29.53	gi|5031851	stathmin isoform a [Homo sapiens]	5	1.00604	0.96349	1.00856	0.924299	1.159543	0.4121	0.99103	0.96627	0.904638	0.20568	1.16846	0.547923
26.6	25.31	gi|119395750	keratin, type II cytoskeletal 1 [Homo sapiens]	19	0.9346	0.43611	0.95497	0.621952	1.108099	0.40979	1.19886	0.10667	1.200578	0.05109	1.04931	0.549805
1.66	10.73	gi|4505641	proliferating cell nuclear antigen [Homo sapiens]	3	0.95874	0.74344	0.76238	0.351143	0.672644	0.18698	0.43342	0.07491	0.500235	0.27736	0.56731	0.556316
17.5	14.43	gi|24234750	interleukin enhancer-binding factor 3 isoform a [Homo sapiens]	10	1.2214	0.42885	1.08192	0.713069	1.193625	0.35476	1.21833	0.49341	0.91235	0.70867	1.04385	0.557202
17.62	37.67	gi|4501891	alpha-actinin-1 isoform b [Homo sapiens]	33	1.14984	0.38813	1.13194	0.32002	1.205048	0.21244	1.01236	0.92856	1.162788	0.20884	1.08371	0.560889
2.12	7.034	gi|4826734	RNA-binding protein FUS isoform 1 [Homo sapiens]	4	0.94968	0.70164	0.86002	0.376919	0.888291	0.45267	0.98973	0.93593	0.969715	0.81381	0.92116	0.568145
4.09	5.954	gi|295842307	splicing factor 1 isoform 4 [Homo sapiens]	3	0.94439	0.91318	1.24603	0.673259	0.931656	0.89132	1.241	0.722	1.023873	0.94625	1.28342	0.570229
5.48	6.397	gi|311078508	histone-binding protein RBBP7 isoform 1 [Homo sapiens]	3	1.10172	0.52866	0.99141	0.969002	0.914266	0.62674	0.9097	0.7392	1.184806	0.19987	0.89257	0.571518
9.97	6.485	gi|33620775	kinectin isoform a [Homo sapiens]	8	1.05966	0.69111	1.19364	0.149146	1.159778	0.26147	0.79517	0.22887	0.90011	0.62253	0.90417	0.572624

2.63	1.025	gi|32967311	ephrin type-A receptor 2 precursor [Homo sapiens]	1	0.9746	0.94795	1.05692	0.85878	1.210665	0.32138	1.1077	0.56043	1.387491	0.37876	1.08739	0.574518
40.89	35.24	gi|6005942	transitional endoplasmic reticulum ATPase [Homo sapiens]	25	0.97357	0.71935	0.95857	0.555716	1.12462	0.17086	1.01768	0.8572	0.946802	0.38291	0.95645	0.575353
6.99	21.4	gi|78000183	60S ribosomal protein L14 [Homo sapiens]	5	1.04335	0.72706	0.99861	0.994141	0.968287	0.89286	1.18263	0.33665	0.920667	0.68297	0.89227	0.575757
3.51	1.903	gi|4504747	integrin alpha-3 isoform a precursor [Homo sapiens]	2	1.226	0.2114	0.8335	0.417073	0.827589	0.60291	1.01131	0.98103	1.119362	0.30086	1.06507	0.578152
6.4	10.41	gi|91199540	dihydrolipoyl dehydrogenase, mitochondrial precursor [Homo sapiens]	4	1.1821	0.51111	1.02828	0.834679	1.282727	0.58737	1.05334	0.70556	1.129976	0.66848	1.19902	0.58019
2.25	3.558	gi|25777612	26S proteasome non-ATPase regulatory subunit 3 [Homo sapiens]	2	0.8381	0.48744	1.10067	0.583873	1.099142	0.73254	0.63133	0.3464	0.970719	0.81572	0.63441	0.581959
11.11	11.62	gi|48255891	glucosidase 2 subunit beta isoform 2 precursor [Homo sapiens]	6	1.03946	0.67934	1.13566	0.081367	1.012411	0.9142	1.13026	0.29235	1.162264	0.01179	1.05193	0.584009
18.97	17.57	gi|4503841	X-ray repair cross-complementing protein 6 [Homo sapiens]	11	0.97292	0.81085	1.00878	0.880034	0.998476	0.98044	0.92188	0.2548	1.058863	0.64942	0.9691	0.58695
7.5	20.54	gi|4506455	reticulocalbin-1 precursor [Homo sapiens]	4	0.9615	0.61475	1.05458	0.619085	0.942759	0.64057	1.04202	0.67339	0.937237	0.68695	0.92712	0.58783
11.48	49.46	gi|7661678	ras-related protein Rap-1b isoform 1 precursor [Homo sapiens]	9	1.04781	0.8059	0.91308	0.460369	1.104292	0.36436	1.11936	0.62485	0.863622	0.22512	1.13417	0.589483

12.36	18.53	gi|57863257	T-complex protein 1 subunit alpha isoform a [Homo sapiens]	6	0.99365	0.97173	1.10198	0.768095	0.820472	0.46263	0.92611	0.65421	1.094258	0.72633	1.15002	0.592124
4	19.33	gi|4506697	40S ribosomal protein S20 isoform 2 [Homo sapiens]	2	1.16724	0.3705	1.38228	0.267105	1.193196	0.66439	1.42943	0.38635	1.142585	0.66025	1.2463	0.592284
2.51	5.183	gi|70995211	delta(3,5)-Delta(2,4)-dienoyl-CoA isomerase, mitochondrial precursor [Homo sapiens]	1	1.02368	0.95347	1.20449	0.732047	0.8886	0.71644	0.98045	0.87509	1.373089	0.27118	1.07641	0.594433
4.25	15.23	gi|68160922	40S ribosomal protein S14 [Homo sapiens]	2	0.89167	0.72053	1.24591	0.353475	1.058112	0.75346	1.02913	0.93338	1.107532	0.68517	1.16347	0.601089
23.39	50	gi|4758950	peptidyl-prolyl cis-trans isomerase B precursor [Homo sapiens]	15	0.96317	0.36944	0.91885	0.055881	0.933665	0.26808	0.99647	0.94972	0.960674	0.37431	0.96843	0.603144
14.36	9.423	gi|154355000	far upstream element-binding protein 2 [Homo sapiens]	8	1.08689	0.50836	1.05982	0.68563	0.932229	0.61558	1.1761	0.473	1.185571	0.19675	1.09494	0.609018
3.71	14.94	gi|62244044	OCIA domain-containing protein 2 isoform 1 [Homo sapiens]	2	1.06593	0.75131	1.07831	0.465847	0.860725	0.46251	0.71107	0.43904	0.881257	0.32917	0.84963	0.609097
6.07	43.44	gi|4885377	histone H1.3 [Homo sapiens]	14	1.04454	0.53785	0.93529	0.437735	0.754462	0.01216	1.21122	0.38623	1.178831	0.3691	0.96051	0.612951
6.48	19.31	gi|4506695	40S ribosomal protein S19 [Homo sapiens]	3	1.18345	0.60921	1.09019	0.861537	0.929737	0.8499	1.1507	0.69475	1.276557	0.41324	1.20276	0.61489
8.68	22.49	gi|354721184	ras-related protein Rab-5C isoform b [Homo sapiens]	5	0.9894	0.89493	1.06862	0.461529	1.002682	0.98651	0.96801	0.8683	0.969061	0.76529	1.08543	0.617855
5.1	19.23	gi|4506743	40S ribosomal protein S8 [Homo sapiens]	7	1.17932	0.49985	1.18919	0.299259	1.224889	0.46026	1.20005	0.31305	1.077038	0.79546	1.1569	0.618747

11.6	25.41	gi|40254924	leucine-rich repeat-containing protein 59 [Homo sapiens]	7	1.17331	0.02623	1.05979	0.327971	0.949168	0.65078	1.1888	0.01927	1.124964	0.16462	1.04358	0.620272
46.54	43.1	gi|4758304	protein disulfide-isomerase A4 precursor [Homo sapiens]	29	0.94767	0.13639	0.94301	0.132998	0.926221	0.06328	0.91517	0.09507	0.961057	0.40127	1.02263	0.620681
183.84	45.56	gi|12667788	myosin-9 [Homo sapiens]	131	0.98833	0.75974	0.97703	0.492793	1.006742	0.82829	0.98619	0.77237	0.960691	0.2	1.01558	0.622695
10.13	10.97	gi|61743954	neuroblast differentiation-associated protein AHNAK isoform 1 [Homo sapiens]	12	0.96465	0.7331	1.01859	0.841517	0.948976	0.63379	0.99368	0.95963	1.066503	0.5159	0.95444	0.625648
4.3	8.462	gi|42734430	polymerase I and transcript release factor [Homo sapiens]	3	0.95085	0.86297	0.89311	0.699758	0.803026	0.67284	0.87765	0.59893	0.805946	0.69619	0.89028	0.628441
5.64	17.87	gi|34147513	ras-related protein Rab-7a [Homo sapiens]	5	0.95351	0.73379	0.76061	0.213252	0.854884	0.35362	0.81528	0.41645	1.001302	0.98901	0.94089	0.631549
6.11	17.26	gi|228008400	heterogeneous nuclear ribonucleoprotein Q isoform 6 [Homo sapiens]	14	1.21817	0.42309	1.24361	0.309094	1.08515	0.76039	1.27522	0.33853	1.195927	0.2873	1.09644	0.632009
11.49	35.05	gi|4506741	40S ribosomal protein S7 [Homo sapiens]	6	1.00409	0.9778	0.96526	0.812921	0.909794	0.66104	0.9513	0.54511	0.966876	0.7708	0.92787	0.634911
10.18	60.5	gi|4757826	beta-2-microglobulin precursor [Homo sapiens]	6	1.26003	0.50144	0.8665	0.306054	1.14102	0.77709	1.42354	0.55364	0.945291	0.79075	0.8252	0.638802
2.1	9.813	gi|223890243	60S ribosomal protein L10 isoform a [Homo sapiens]	2	1.14824	0.20993	1.03798	0.754032	1.268798	0.08304	1.15213	0.28899	1.087423	0.68281	1.04897	0.641001

16.25	45.18	gi|153070260	myristoylated alanine-rich C-kinase substrate [Homo sapiens]	9	0.983	0.80578	0.9737	0.694966	0.988063	0.84891	0.99415	0.92598	0.997562	0.96911	1.03013	0.641041
34.42	55.7	gi|156071459	ADP/ATP translocase 2 [Homo sapiens]	29	1.03376	0.68358	0.9262	0.376592	0.988751	0.95299	0.97673	0.78837	0.96945	0.77703	0.9515	0.641079
2.17	0.4867	gi|54607053	translational activator GCN1 [Homo sapiens]	1	1.28676	0.24123	1.07193	0.906543	0.741843	0.68752	0.71728	0.66122	1.331493	0.53099	0.83414	0.644476
2.39	5.917	gi|4502981	cytochrome c oxidase subunit 4 isoform 1, mitochondrial precursor [Homo sapiens]	1	0.86484	0.25424	0.99029	0.964486	1.020714	0.83282	0.8429	0.23318	1.026772	0.81804	1.0846	0.645195
1.43	0.9151	gi|29826341	calmodulin-binding transcription activator 2 isoform 1 [Homo sapiens]	2	1.02264	0.97348	1.1667	0.370435	1.013866	0.97412	1.00199	0.99841	0.997553	0.99617	0.82669	0.646072
18	89.63	gi|4504981	galectin-1 [Homo sapiens]	13	0.94609	0.56748	1.06686	0.337019	0.998775	0.99189	0.98275	0.86625	0.982519	0.85361	0.96945	0.646657
30.18	31.63	gi|34932414	non-POU domain-containing octamer-binding protein isoform 1 [Homo sapiens]	18	1.02179	0.71872	0.95014	0.56712	0.987357	0.85793	1.00228	0.97442	0.979062	0.76057	0.95323	0.661185
20.86	21.51	gi|18201905	glucose-6-phosphate isomerase isoform 2 [Homo sapiens]	14	0.8891	0.11886	0.99188	0.914596	1.007987	0.93038	0.92749	0.67746	1.005971	0.94939	1.03989	0.662333
1.69	10.67	gi|4758040	cytochrome c oxidase subunit 6C proprotein [Homo sapiens]	2	0.85609	0.37626	1.19072	0.565045	0.999303	0.99786	1.33632	0.67643	1.130728	0.64572	1.06285	0.662841
6.13	6.647	gi|87196351	ATP-dependent RNA helicase DDX3X isoform 1 [Homo sapiens]	3	1.18019	0.51813	1.21877	0.602725	0.919683	0.75389	0.73428	0.20041	1.18035	0.41588	1.05972	0.665489

37.8	58.07	gi|14043072	heterogeneous nuclear ribonucleoproteins A2/B1 isoform B1 [Homo sapiens]	24	0.99729	0.99214	0.89746	0.632954	0.951845	0.82147	1.04794	0.90827	0.952007	0.78644	0.91491	0.666499
9.87	15.36	gi|15055539	40S ribosomal protein S2 [Homo sapiens]	4	0.98191	0.94926	1.00864	0.979743	0.878588	0.57863	1.02254	0.91929	0.956993	0.88491	1.1123	0.670417
4.67	11.41	gi|4506649	60S ribosomal protein L3 isoform a [Homo sapiens]	3	0.86881	0.71238	0.92019	0.286429	0.938384	0.8218	0.99791	0.9954	0.829736	0.47181	0.90558	0.673162
3.95	2.855	gi|122939155	NAD(P) transhydrogenase, mitochondrial [Homo sapiens]	3	1.00797	0.94948	1.13779	0.597416	1.143784	0.49699	0.8888	0.4475	0.881076	0.48103	1.13072	0.673925
11.05	29.93	gi|63252900	tropomyosin alpha-1 chain isoform 4 [Homo sapiens]	10	1.08825	0.37639	1.04674	0.732007	1.130557	0.28152	1.16461	0.14713	0.981288	0.89392	1.04555	0.674296
22.02	20.65	gi|4826998	splicing factor, proline- and glutamine-rich [Homo sapiens]	19	0.97461	0.91414	1.03224	0.88877	0.887348	0.62919	0.97859	0.93981	1.022428	0.92594	0.90595	0.674537
4.58	17.62	gi|251831110	cytochrome c oxidase subunit II [Homo sapiens]	3	0.80403	0.69103	0.86297	0.299261	0.722846	0.28015	0.6475	0.10402	0.866816	0.66375	0.91801	0.675132
6.22	19.68	gi|4506607	60S ribosomal protein L18 isoform 1 [Homo sapiens]	3	0.96035	0.7603	0.87805	0.465616	0.962498	0.839	0.88382	0.53872	1.032148	0.81521	0.89035	0.675519
16.23	74.51	gi|4504523	10 kDa heat shock protein, mitochondrial [Homo sapiens]	8	0.99268	0.88544	0.97341	0.600927	0.974775	0.61892	0.97012	0.56798	0.974523	0.6162	1.02344	0.675919
9.51	19.44	gi|67189747	60S ribosomal protein L6 [Homo sapiens]	5	1.02062	0.76923	0.84308	0.303084	1.054461	0.51107	1.04638	0.62651	0.978707	0.85244	0.95504	0.677122
7.3	29.17	gi|67944630	60S ribosomal protein L9 [Homo sapiens]	5	1.15467	0.38103	1.15642	0.137453	1.229212	0.2788	0.96642	0.85104	1.288677	0.12373	0.93631	0.677782

2.18	2.71	gi|19923193	hsc70-interacting protein [Homo sapiens]	1	0.95037	0.83989	0.96798	0.821141	0.800799	0.76442	1.21002	0.30454	0.833078	0.31592	0.84992	0.678324
13.2	29.47	gi|55956921	heterogeneous nuclear ribonucleoprotein A/B isoform b [Homo sapiens]	9	1.0958	0.21875	0.95419	0.514181	0.998728	0.98187	1.06164	0.42607	1.077823	0.36657	1.03855	0.682577
4.49	15.75	gi|217330646	activated RNA polymerase II transcriptional coactivator p15 [Homo sapiens]	2	1.00545	0.95064	0.93514	0.513028	0.866581	0.5256	0.9196	0.27018	0.941666	0.70261	0.93123	0.689454
7.02	12.25	gi|10835067	lupus La protein [Homo sapiens]	4	0.89702	0.81509	1.02335	0.970048	0.912524	0.7839	0.7299	0.73114	0.740666	0.69847	0.84575	0.690483
4.6	48.99	gi|156071462	ADP/ATP translocase 3 [Homo sapiens]	27	1.12804	0.72644	1.12233	0.745559	1.261938	0.52519	1.17129	0.68662	1.088808	0.69381	1.11963	0.691645
4.14	16.17	gi|39725636	transmembrane emp24 domain-containing protein 9 precursor [Homo sapiens]	3	1.09319	0.53296	1.04374	0.761259	1.333761	0.26105	0.73663	0.19907	0.970606	0.87339	1.08416	0.691946
4.38	12.82	gi|16117789	60S ribosomal protein L34 [Homo sapiens]	2	0.87852	0.5603	0.88838	0.470693	0.772053	0.45763	0.59708	0.13049	0.960721	0.77356	0.75407	0.692607
28.84	30.2	gi|50345984	ATP synthase subunit alpha, mitochondrial isoform a precursor [Homo sapiens]	21	0.95209	0.43118	0.97584	0.817633	0.944751	0.56027	0.99797	0.98171	0.942627	0.43909	1.03342	0.693136
20.15	55.88	gi|4504297	histone H3.1 [Homo sapiens]	20	1.11344	0.49506	0.89675	0.505913	0.998635	0.99393	1.19646	0.59173	0.925642	0.59705	0.94698	0.696575
19.47	39.46	gi|221307584	prohibitin-2 isoform 1 [Homo sapiens]	14	1.03343	0.73666	0.93609	0.6425	0.962379	0.72427	0.95351	0.83651	0.943864	0.59801	1.0389	0.697615
18.66	33.42	gi|93141020	core histone macro-H2A.1 isoform 2 [Homo sapiens]	12	1.03657	0.80111	0.98151	0.885185	1.032144	0.80074	0.93467	0.77516	1.039574	0.78897	0.94747	0.698714

8.92	17.45	gi|47132595	phosphate carrier protein, mitochondrial isoform b precursor [Homo sapiens]	5	1.06729	0.32103	0.95402	0.461468	1.101687	0.16278	0.99838	0.98638	1.049733	0.44725	1.04113	0.699173
6.19	27.91	gi|17017988	cytochrome c oxidase subunit 5B, mitochondrial precursor [Homo sapiens]	4	1.01292	0.91838	1.27302	0.344714	0.87403	0.55306	0.53226	0.34154	1.014991	0.92652	0.92066	0.701532
16.02	11.59	gi|48255935	CD44 antigen isoform 1 precursor [Homo sapiens]	9	0.99745	0.96815	1.05606	0.470139	0.974859	0.77069	1.02163	0.80288	0.952887	0.7142	0.95638	0.702046
6.26	41.78	gi|83376130	elongation factor 1-beta [Homo sapiens]	5	1.06939	0.52176	0.99888	0.998173	1.26491	0.11474	1.26122	0.61166	1.299958	0.42094	1.17718	0.702982
24.64	16.54	gi|23510340	ubiquitin-like modifier-activating enzyme 1 [Homo sapiens]	17	0.86945	0.13185	0.89243	0.154395	1.047217	0.59847	0.8588	0.06201	0.98632	0.84641	1.03481	0.70466
2.08	13.04	gi|5453559	ATP synthase subunit d, mitochondrial isoform a [Homo sapiens]	1	1.54649	0.1424	1.23522	0.663545	1.086338	0.55674	1.28814	0.2495	1.425763	0.40471	1.21003	0.705296
12.34	2.626	gi|41322916	plectin isoform 1 [Homo sapiens]	10	0.95217	0.8024	1.34488	0.401625	1.034696	0.93365	1.19649	0.63772	1.423922	0.15327	1.12662	0.707928
16.48	32.53	gi|14043070	heterogeneous nuclear ribonucleoprotein A1 isoform b [Homo sapiens]	23	1.05591	0.75886	0.96459	0.690319	1.149365	0.41417	1.20924	0.58318	1.019104	0.89852	1.07093	0.708404
4.16	12.32	gi|6912634	60S ribosomal protein L13a isoform 1 [Homo sapiens]	3	0.95479	0.81055	0.87337	0.259546	1.140071	0.34559	0.89491	0.55672	1.120049	0.45983	0.95566	0.711813
10.8	26.28	gi|21614499	ezrin [Homo sapiens]	15	1.06182	0.49832	1.05333	0.555483	1.307711	0.03294	0.94707	0.71822	1.133167	0.4463	0.89818	0.712349

24.74	21.58	gi|4506675	dolichyl-diphosphooligosaccharide--protein glycosyltransferase subunit 1 precursor [Homo sapiens]	12	1.05826	0.57928	0.982	0.858546	0.991824	0.94215	1.03226	0.81042	0.962578	0.72136	1.03882	0.713397
7.75	14.9	gi|19743875	fumarate hydratase, mitochondrial [Homo sapiens]	5	1.23102	0.54474	0.98192	0.924664	1.139205	0.30063	0.85337	0.73457	1.058899	0.90011	1.1213	0.713871
5.8	15.3	gi|52630342	HLA class I histocompatibility antigen, Cw-1 alpha chain precursor [Homo sapiens]	6	0.97171	0.88809	1.02558	0.936087	1.027002	0.79844	0.88162	0.32984	0.853302	0.58819	0.90592	0.715453
20.77	65.58	gi|380837121	vesicle-trafficking protein SEC22b precursor [Homo sapiens]	20	1.10024	0.05719	1.03229	0.495044	1.038818	0.5283	1.06556	0.32572	0.996953	0.97062	1.03531	0.723297
4.52	5.752	gi|394025723	very long-chain specific acyl-CoA dehydrogenase, mitochondrial isoform 3 [Homo sapiens]	3	0.74699	0.59182	0.76043	0.55318	0.988837	0.95255	0.65897	0.26758	0.911972	0.79251	0.72594	0.725427
10.1	22.11	gi|42476281	voltage-dependent anion-selective channel protein 2 isoform 2 [Homo sapiens]	7	0.98794	0.90701	0.94137	0.558142	0.967298	0.81086	1.19774	0.20741	0.999744	0.998	0.97538	0.726116
4.15	7.612	gi|21536286	creatine kinase B-type [Homo sapiens]	2	0.93056	0.85493	0.75114	0.745502	0.851942	0.75556	0.70757	0.23455	0.726465	0.72965	0.86017	0.728049
9.25	22.58	gi|15431288	60S ribosomal protein L10a [Homo sapiens]	5	1.13923	0.20613	1.19379	0.097845	1.405531	0.07869	1.30502	0.03712	1.272751	0.04776	1.06428	0.731592

4.26	6.093	gi|4885281	glutamate dehydrogenase 1, mitochondrial precursor [Homo sapiens]	3	1.03466	0.82658	1.31687	0.384031	0.988878	0.95217	1.03104	0.84229	0.9713	0.90753	1.04757	0.734971
18.88	39.92	gi|378548190	40S ribosomal protein S3 isoform 1 [Homo sapiens]	10	1.05379	0.67765	0.90108	0.325676	0.998495	0.98928	0.95251	0.73441	1.080897	0.54587	1.05154	0.736446
8.52	28.89	gi|4502205	ADP-ribosylation factor 4 [Homo sapiens]	5	1.12509	0.85061	0.88053	0.643544	1.213056	0.76438	0.7777	0.57084	0.860941	0.67797	0.83245	0.740092
12.97	11.04	gi|52632383	heterogeneous nuclear ribonucleoprotein L isoform a [Homo sapiens]	6	1.1947	0.19772	1.03175	0.790467	1.158331	0.37641	1.06595	0.63342	0.987748	0.89712	1.04132	0.742688
3.73	10.65	gi|321117084	RPS10-NUDT3 protein [Homo sapiens]	3	1.01139	0.94355	1.0143	0.970992	0.922407	0.5861	1.00405	0.96666	1.059227	0.64926	0.96851	0.743459
7.8	50.71	gi|4506605	60S ribosomal protein L23 [Homo sapiens]	5	0.87865	0.74322	0.95354	0.771566	0.815871	0.53752	1.04347	0.881	0.760368	0.11783	0.92817	0.750798
3.98	18.67	gi|190885499	cytochrome c oxidase subunit 5A, mitochondrial precursor [Homo sapiens]	3	1.04812	0.65287	1.12766	0.567805	0.993509	0.97116	0.77747	0.09934	1.026415	0.78893	1.04016	0.752238
1.35	27.27	gi|194688135	high mobility group protein B2 [Homo sapiens]	7	1.06464	0.85993	1.01726	0.972515	1.015592	0.97689	1.02219	0.96616	0.999257	0.99421	0.91965	0.760339
7.61	21.92	gi|98986464	transmembrane emp24 domain-containing protein 10 precursor [Homo sapiens]	6	0.88786	0.72101	0.78503	0.248243	0.818962	0.61315	0.91388	0.79432	0.896156	0.53912	0.92621	0.763469
9.7	4.637	gi|19913406	DNA topoisomerase 2-alpha [Homo sapiens]	7	1.19997	0.81665	1.39591	0.184234	1.329365	0.30032	1.49119	0.45954	1.127594	0.8554	1.14999	0.764389

5.87	19.02	gi|5803036	heterogeneous nuclear ribonucleoprotein A0 [Homo sapiens]	5	1.05968	0.83796	0.97227	0.854566	1.114078	0.49743	1.07709	0.79112	1.012741	0.96513	1.05259	0.765253
19.03	48.66	gi|4758638	peroxiredoxin-6 [Homo sapiens]	9	0.96744	0.76701	1.1843	0.204936	1.193197	0.07085	0.98306	0.94703	1.00903	0.97102	1.07972	0.767286
9.78	10.43	gi|4502285	sarcoplasmic/endoplasmic reticulum calcium ATPase 2 isoform a [Homo sapiens]	7	1.10151	0.66208	1.04782	0.815902	1.335057	0.31594	1.0606	0.84338	1.01103	0.93739	1.06943	0.767545
10.29	26.43	gi|73760405	thymopoietin isoform beta [Homo sapiens]	7	1.05181	0.61144	1.12399	0.350742	0.91077	0.54197	0.84554	0.35827	0.975897	0.80234	0.96391	0.768285
8.66	73.91	gi|4506671	60S acidic ribosomal protein P2 [Homo sapiens]	7	1.08556	0.34581	1.0435	0.605096	1.058493	0.49566	1.11581	0.23312	1.056699	0.50924	1.0239	0.768286
5.06	12.77	gi|163644321	cytochrome b-c1 complex subunit Rieske, mitochondrial [Homo sapiens]	3	0.75455	0.71922	0.57239	0.407115	0.771567	0.45298	0.86607	0.39779	0.948623	0.69406	0.84441	0.772512
4.75	14.44	gi|311771647	actin-related protein 2/3 complex subunit 4 isoform c [Homo sapiens]	2	1.08265	0.87616	0.96313	0.925078	1.106502	0.78565	0.72989	0.67988	0.828008	0.34231	1.1508	0.773513
62.88	65.79	gi|41399285	60 kDa heat shock protein, mitochondrial [Homo sapiens]	57	1.00869	0.83404	1.0126	0.787426	1.000185	0.99618	0.99905	0.9821	0.998881	0.96892	1.00977	0.773751
20.62	36.93	gi|14110414	heterogeneous nuclear ribonucleoprotein D0 isoform c [Homo sapiens]	11	0.99292	0.95083	0.93943	0.765849	0.999996	1	0.90384	0.55038	0.911699	0.637	0.96899	0.774905
6.7	31.82	gi|14277700	40S ribosomal protein S12 [Homo sapiens]	5	1.01814	0.89192	0.83278	0.636779	1.036141	0.79088	1.09946	0.53195	0.947092	0.70597	1.03925	0.774943

8.55	43.03	gi|4506597	60S ribosomal protein L12 [Homo sapiens]	5	1.12104	0.41768	0.99867	0.991624	1.142792	0.27762	1.15111	0.36571	1.168818	0.22156	0.97404	0.775656
2.14	1.183	gi|69354671	ATP-binding cassette sub-family F member 1 isoform a [Homo sapiens]	1	0.97085	0.91323	1.37039	0.194106	1.471155	0.16004	0.99946	0.99909	1.19076	0.84283	0.88838	0.777605
8.3	15.77	gi|38455427	T-complex protein 1 subunit delta isoform a [Homo sapiens]	9	1.00106	0.99697	0.9324	0.78378	0.94696	0.89777	0.73934	0.19109	0.953969	0.86496	0.88244	0.781297
3.55	3.568	gi|25777600	26S proteasome non-ATPase regulatory subunit 1 isoform 1 [Homo sapiens]	2	0.5996	0.2571	0.72519	0.44323	0.696634	0.17297	0.78612	0.64533	0.951232	0.78597	0.90594	0.783538
11.9	37.56	gi|4758988	ras-related protein Rab-1A isoform 1 [Homo sapiens]	6	0.99181	0.96521	0.92253	0.748232	0.935789	0.76268	0.90523	0.4775	0.908909	0.59598	0.95635	0.786256
3.24	16.67	gi|7706244	protein CutA isoform 2 precursor [Homo sapiens]	2	0.67103	0.589	1.19653	0.60623	1.002143	0.99217	0.80534	0.65969	0.637288	0.47229	0.87021	0.787415
2.13	1.915	gi|338827685	AP-2 complex subunit alpha-2 isoform 1 [Homo sapiens]	2	0.71448	0.18213	1.31353	0.221365	1.383797	0.18813	0.99862	0.9911	0.962428	0.76468	0.92112	0.789026
9.74	10.09	gi|20070197	dolichyl-diphosphooligosaccharide--protein glycosyltransferase 48 kDa subunit precursor [Homo sapiens]	4	1.04981	0.70743	0.9536	0.607867	1.072354	0.48364	1.0482	0.65371	1.145003	0.10928	0.95791	0.795514
5.68	1.27	gi|114155142	nucleoprotein TPR [Homo sapiens]	3	0.95437	0.71923	1.55827	0.139745	1.144505	0.69242	1.20935	0.80201	1.199909	0.83418	1.21081	0.797233
6.36	5.361	gi|42544159	heat shock protein 105 kDa [Homo sapiens]	5	1.03791	0.83751	0.71001	0.495345	0.921183	0.61123	0.87039	0.52533	0.997984	0.98476	0.95339	0.800479

11.37	31.56	gi|4502491	complement component 1 Q subcomponent-binding protein, mitochondrial precursor [Homo sapiens]	9	1.00508	0.9556	1.08064	0.456452	1.010557	0.92983	1.20337	0.33467	1.019807	0.90324	0.91517	0.802382
5.75	7.296	gi|21361368	delta-1-pyrroline-5-carboxylate synthase isoform 1 [Homo sapiens]	3	0.9781	0.9669	0.85497	0.50659	0.897332	0.73533	0.78013	0.6573	0.641257	0.47964	1.06857	0.80268
6.4	18.54	gi|4506685	40S ribosomal protein S13 [Homo sapiens]	3	0.99031	0.93996	0.84468	0.333348	1.051426	0.71728	0.7756	0.29608	0.912275	0.4263	0.97195	0.802778
14.95	30.7	gi|4502923	calponin-3 [Homo sapiens]	15	1.02544	0.70414	1.05062	0.341917	0.941943	0.35536	0.95816	0.58435	1.044902	0.49742	1.01242	0.806483
8.48	9.269	gi|29029559	exportin-2 isoform 1 [Homo sapiens]	5	1.09982	0.8148	0.8955	0.765058	1.387637	0.56142	0.88907	0.77044	0.874732	0.85614	1.12938	0.809937
8.48	9.269	gi|29029559	exportin-2 isoform 1 [Homo sapiens]	5	1.09982	0.8148	0.8955	0.765058	1.387637	0.56142	0.88907	0.77044	0.874732	0.85614	1.12938	0.809937
14.77	50.49	gi|77539758	histone H4 [Homo sapiens]	28	1.11512	0.252	0.94207	0.44027	1.094707	0.25106	1.24563	0.0212	0.995098	0.94742	1.09363	0.814591
4.48	43.75	gi|4506613	60S ribosomal protein L22 proprotein [Homo sapiens]	5	0.92582	0.39406	0.88941	0.431866	0.985671	0.87993	1.04055	0.82157	0.807588	0.15141	1.03663	0.815932
24.64	51.1	gi|4505773	prohibitin [Homo sapiens]	12	0.96556	0.73689	1.04412	0.592809	1.132968	0.46259	1.04172	0.8169	1.082056	0.61104	0.97867	0.817195
11.24	18.99	gi|4503481	elongation factor 1-gamma [Homo sapiens]	6	1.03165	0.89414	0.94509	0.835196	1.099946	0.45211	0.90037	0.57359	1.077196	0.62388	1.04039	0.819465
6.64	18.97	gi|7330335	chloride intracellular channel protein 4 [Homo sapiens]	4	1.02164	0.91147	1.13859	0.422729	1.466823	0.16472	1.11304	0.48267	1.016055	0.92994	0.97107	0.819961
4.29	30.6	gi|20544151	chromobox protein homolog 3 [Homo sapiens]	3	1.08761	0.76023	1.14249	0.606873	0.916121	0.50274	1.29959	0.53768	0.975734	0.92692	1.07487	0.821474

8.04	9.255	gi|5730027	KH domain-containing, RNA-binding, signal transduction-associated protein 1 [Homo sapiens]	5	1.06558	0.65957	0.93595	0.427594	0.998715	0.99478	0.95233	0.74678	0.92066	0.80624	0.96775	0.822087
4.95	3.383	gi|19743823	integrin beta-1 isoform 1A precursor [Homo sapiens]	3	1.32055	0.10167	1.13929	0.259019	1.350968	0.06939	1.04784	0.8101	1.521222	0.03788	1.08382	0.82873
172.41	48.02	gi|160420317	filamin-A isoform 2 [Homo sapiens]	119	0.97842	0.41197	0.99518	0.85802	0.967653	0.27161	0.98363	0.61147	0.984986	0.55125	0.99244	0.828854
15.29	20.61	gi|16579885	60S ribosomal protein L4 [Homo sapiens]	7	1.07615	0.33021	1.01311	0.924931	1.084191	0.48575	1.04919	0.72858	0.997751	0.98856	0.9789	0.833723
14.5	32.11	gi|4504041	guanine nucleotide-binding protein G(i) subunit alpha-2 isoform 1 [Homo sapiens]	9	1.13704	0.56315	0.96894	0.813532	1.020694	0.96158	0.93776	0.64576	1.055722	0.81975	1.02806	0.8344
6.42	16.02	gi|5802974	thioredoxin-dependent peroxide reductase, mitochondrial isoform a precursor [Homo sapiens]	4	0.96798	0.81028	0.95634	0.515702	1.033963	0.62264	1.22076	0.55074	0.991637	0.90516	1.02995	0.835193
2.7	9.494	gi|4506681	40S ribosomal protein S11 [Homo sapiens]	2	0.99409	0.97465	1.13149	0.545044	1.047803	0.88522	1.08859	0.77372	0.982615	0.92298	1.06109	0.835841
8.42	9.772	gi|154354966	mitochondrial inner membrane protein isoform 3 [Homo sapiens]	5	1.0206	0.93464	1.03532	0.871746	0.988825	0.96777	0.9628	0.86904	1.0296	0.89968	0.96498	0.837598
4.45	61.11	gi|28173554	histone H2B type 3-B [Homo sapiens]	43	1.02072	0.90655	0.96056	0.861582	1.037087	0.84719	1.18373	0.44769	0.948548	0.65963	1.0357	0.838179
6.43	18.29	gi|4506901	serine/arginine-rich splicing factor 3 [Homo sapiens]	3	0.9852	0.96279	1.1985	0.438019	1.078372	0.69505	1.06264	0.90255	0.984337	0.93061	0.92951	0.838933

2.06	18.42	gi|4507793	ubiquitin-conjugating enzyme E2 N [Homo sapiens]	1	1.16792	0.65472	1.21634	0.809184	1.932162	0.44384	1.52095	0.40788	0.920573	0.83344	1.21791	0.840396
1.92	7.042	gi|5174449	histone H1x [Homo sapiens]	1	0.85343	0.28064	0.85981	0.345885	0.741735	0.07861	0.83554	0.26722	0.963425	0.82536	1.04754	0.841578
2.64	38.34	gi|10835049	transforming protein RhoA precursor [Homo sapiens]	8	0.99703	0.98135	0.79242	0.729798	0.851819	0.80345	1.07826	0.72817	0.687668	0.33072	0.822	0.841902
3.96	3.659	gi|166795299	solute carrier family 2, facilitated glucose transporter member 1 [Homo sapiens]	2	1.04935	0.91109	0.94918	0.915556	0.754969	0.25409	1.11376	0.68062	1.154167	0.39811	1.02541	0.847455
2.29	20.38	gi|5803137	putative RNA-binding protein 3 [Homo sapiens]	1	1.08481	0.88279	0.85746	0.624625	0.849487	0.80461	1.20187	0.86022	0.779833	0.50567	0.93073	0.84798
2.51	2.204	gi|7661936	scaffold attachment factor B2 [Homo sapiens]	2	1.01899	0.91967	1.04755	0.836359	1.058761	0.80586	0.94253	0.81642	1.236486	0.51996	0.95485	0.848025
9.61	16.74	gi|73486658	aspartate aminotransferase, mitochondrial precursor [Homo sapiens]	5	0.96036	0.87103	0.97537	0.860831	1.070459	0.6887	1.07102	0.52078	1.011362	0.91313	1.03523	0.849832
6.79	29.52	gi|50592994	thioredoxin isoform 1 [Homo sapiens]	4	0.87285	0.42193	1.06975	0.476514	1.075307	0.44631	0.97736	0.91538	0.937442	0.49301	0.9463	0.854211
23.82	61.48	gi|4507879	voltage-dependent anion-selective channel protein 1 [Homo sapiens]	14	0.97731	0.69263	0.92668	0.34112	0.913339	0.34029	0.97341	0.74774	0.978336	0.79238	1.0166	0.858966
3.32	12.62	gi|4758018	calponin-2 isoform a [Homo sapiens]	3	1.19158	0.71105	1.19474	0.741549	0.728561	0.79122	1.01345	0.96606	0.868569	0.78228	1.14008	0.860278
4.75	26.37	gi|299523086	mesencephalic astrocyte-derived neurotrophic factor precursor [Homo sapiens]	5	1.11545	0.57209	1.11679	0.585957	1.029277	0.91557	1.01628	0.94928	1.076269	0.66921	1.04584	0.860548

7.7	20.63	gi|34740329	heterogeneous nuclear ribonucleoprotein A3 [Homo sapiens]	10	1.10445	0.17571	0.9032	0.316306	1.145317	0.40173	1.07986	0.8933	1.122994	0.34016	1.02019	0.860986
6.05	10.77	gi|117168248	3-hydroxyacyl-CoA dehydratase 3 [Homo sapiens]	4	0.84957	0.44745	0.8334	0.161112	1.038632	0.8902	0.95966	0.90221	1.045877	0.88382	0.9249	0.862774
26.92	24.93	gi|14141152	heterogeneous nuclear ribonucleoprotein M isoform a [Homo sapiens]	17	1.08628	0.35293	1.03725	0.695026	1.026806	0.73823	0.99633	0.96725	1.016342	0.83824	0.98941	0.873553
3.23	12.07	gi|170763500	protein SET isoform 1 [Homo sapiens]	4	1.0848	0.69043	1.03571	0.725061	1.088743	0.67097	0.98089	0.84488	1.116083	0.53368	0.97681	0.873979
12.24	64.24	gi|88999583	myosin light polypeptide 6 isoform 2 [Homo sapiens]	15	0.94267	0.343	0.9248	0.245371	0.932394	0.26936	0.99883	0.9843	0.985798	0.8209	0.9908	0.876608
2.04	11.88	gi|63025214	vacuolar ATPase assembly integral membrane protein VMA21 [Homo sapiens]	1	0.90619	0.51417	0.99561	0.972821	1.057661	0.75898	1.18775	0.61068	1.014332	0.97255	0.96105	0.878343
7.9	9.387	gi|29789090	protein RCC2 [Homo sapiens]	4	1.11316	0.66394	1.06546	0.807756	1.060682	0.8303	0.96863	0.86843	1.225477	0.32522	1.03343	0.879579
27.24	41.98	gi|117190254	heterogeneous nuclear ribonucleoproteins C1/C2 isoform b [Homo sapiens]	17	1.02698	0.67678	0.92177	0.182329	0.974944	0.74372	1.04255	0.69517	0.976558	0.74628	0.99	0.88068
18.26	15.39	gi|189458819	transferrin receptor protein 1 [Homo sapiens]	11	1.11446	0.42576	0.99792	0.991522	1.124781	0.5508	1.14668	0.4871	0.988161	0.94215	0.94789	0.881912
8.03	10.46	gi|63162572	T-complex protein 1 subunit gamma isoform a [Homo sapiens]	4	0.82695	0.56059	0.81169	0.577272	1.153473	0.80554	0.82045	0.75663	1.050539	0.85656	0.93205	0.890922

2.38	5.536	gi|11056044	inorganic pyrophosphatase [Homo sapiens]	1	1.04929	0.9395	0.99655	0.995222	1.184591	0.80537	1.15813	0.82207	1.036608	0.79363	1.15318	0.891584
2.5	0.6877	gi|56676335	telomere-associated protein RIF1 isoform 1 [Homo sapiens]	1	1.08279	0.66024	0.87066	0.777724	0.618766	0.34994	0.55636	0.52788	0.984399	0.9835	0.92906	0.892763
4.92	16.95	gi|315221152	60S ribosomal protein L11 isoform 2 [Homo sapiens]	4	1.06126	0.56421	0.99761	0.975118	1.065146	0.43782	0.95199	0.536	1.085682	0.32948	0.98499	0.89321
11.85	27.44	gi|4504425	high mobility group protein B1 [Homo sapiens]	8	1.025	0.85145	0.92685	0.563656	0.927637	0.65527	1.01212	0.93795	0.957028	0.6268	0.97689	0.893937
13.77	28.61	gi|24308201	adipocyte plasma membrane-associated protein [Homo sapiens]	10	0.83601	0.21067	1.06307	0.479644	0.886176	0.51837	0.86221	0.20778	0.968326	0.63722	1.03196	0.894531
4.42	26.57	gi|4506701	40S ribosomal protein S23 [Homo sapiens]	3	0.87112	0.40648	0.72728	0.199117	0.817343	0.42284	0.7103	0.30555	1.074612	0.66576	1.01694	0.896721
8.13	45.19	gi|4506693	40S ribosomal protein S17 [Homo sapiens]	6	0.99431	0.97242	0.97255	0.722537	1.038886	0.75248	1.03874	0.67553	1.034698	0.66409	0.98707	0.899802
10.13	20.93	gi|5453603	T-complex protein 1 subunit beta isoform 1 [Homo sapiens]	6	1.09951	0.79704	1.08657	0.641929	1.428159	0.05029	1.03563	0.83282	0.912354	0.81703	1.02791	0.899862
5.67	46.15	gi|21464101	14-3-3 protein gamma [Homo sapiens]	15	1.02568	0.83239	1.03997	0.769753	0.969569	0.85613	0.90003	0.52358	0.991797	0.94948	0.96655	0.900023
16.61	9.229	gi|256222411	filamin-B isoform 1 [Homo sapiens]	20	0.98336	0.8992	1.02555	0.853117	1.118949	0.54084	0.99812	0.9923	0.991695	0.90764	0.97554	0.900285
4.3	11.45	gi|388240801	lamin-B2 [Homo sapiens]	7	0.87942	0.71958	1.10445	0.77971	0.78914	0.61079	0.91952	0.83412	0.95001	0.85965	0.94256	0.901109
1.3	2.058	gi|39753957	torsin-1A-interacting protein 1 isoform 2 [Homo sapiens]	1	0.83348	0.33185	0.96138	0.867541	1.103016	0.62859	0.87927	0.53707	1.083103	0.74121	1.05113	0.903757

9.32	22.16	gi|14141193	40S ribosomal protein S9 [Homo sapiens]	5	0.99992	0.99954	0.92862	0.763386	0.945508	0.76024	0.95503	0.83905	1.075762	0.60233	1.01905	0.904019
8.11	27.47	gi|34098946	nuclease-sensitive element-binding protein 1 [Homo sapiens]	5	1.07413	0.56558	1.14988	0.476996	1.06747	0.59239	1.0434	0.82634	1.220969	0.45264	1.0157	0.90823
2.15	8.894	gi|42822874	ribonuclease inhibitor [Homo sapiens]	2	0.80302	0.27929	0.90655	0.700213	1.344467	0.6081	1.12269	0.45072	1.136887	0.41847	0.98588	0.909263
3.12	2.452	gi|225543215	antigen KI-67 isoform 2 [Homo sapiens]	4	1.03006	0.8092	0.87668	0.728422	1.203502	0.26766	1.24771	0.44123	1.27859	0.18565	1.03634	0.910268
3.52	50.74	gi|4885385	histone H3.3 [Homo sapiens]	11	0.96893	0.81563	0.94274	0.67602	1.054957	0.7012	0.9351	0.87652	1.046815	0.7414	1.02837	0.910912
7.22	22.4	gi|4506457	reticulocalbin-2 precursor [Homo sapiens]	5	1.38471	0.39167	1.2916	0.445955	1.296941	0.47927	1.43781	0.41555	1.536593	0.26422	1.06997	0.911035
1.65	2.682	gi|50659095	nucleolar RNA helicase 2 isoform 1 [Homo sapiens]	2	1.01952	0.95996	1.38491	0.187652	1.017043	0.89225	1.16703	0.36259	0.984875	0.91557	0.97993	0.915927
3.63	9.016	gi|5031777	isocitrate dehydrogenase [NAD] subunit alpha, mitochondrial precursor [Homo sapiens]	2	1.16688	0.3754	1.19345	0.716846	1.497555	0.3531	1.59903	0.20131	1.23718	0.57148	1.03029	0.917579
12.62	25	gi|15431301	60S ribosomal protein L7 [Homo sapiens]	7	0.92425	0.48551	1.03374	0.876283	0.95133	0.5483	0.99818	0.99234	1.082148	0.57258	1.0118	0.917983
4.7	24	gi|256222019	ras-related protein Rab-10 [Homo sapiens]	5	0.94102	0.6384	0.90518	0.403307	0.902896	0.68559	0.80889	0.38432	0.987171	0.95238	0.95457	0.918074

3.12	2.979	gi|22749415	dolichyl-diphosphooligosaccharide--protein glycosyltransferase subunit STT3A [Homo sapiens]	1	1.06993	0.62139	1.02313	0.947688	0.891197	0.48906	1.44778	0.43354	1.22369	0.29182	0.94878	0.91885
2.09	5.682	gi|4758788	NADH dehydrogenase [ubiquinone] iron-sulfur protein 3, mitochondrial precursor [Homo sapiens]	1	1.09954	0.75239	1.00194	0.98818	1.04381	0.83939	1.10044	0.84765	1.189316	0.20548	1.01421	0.919937
9.08	21.92	gi|4503607	electron transfer flavoprotein subunit alpha, mitochondrial isoform a [Homo sapiens]	7	0.745	0.58946	1.25831	0.663793	0.924426	0.79947	0.83736	0.64983	0.555631	0.57613	0.93131	0.921689
2.45	7.143	gi|4759160	small nuclear ribonucleoprotein Sm D3 [Homo sapiens]	1	0.90276	0.57568	0.9279	0.468609	0.88426	0.48308	0.81171	0.52085	0.831009	0.17558	0.98806	0.923847
5.42	23.08	gi|4758302	enhancer of rudimentary homolog [Homo sapiens]	3	1.03392	0.80889	1.12985	0.460855	1.232816	0.30435	1.13608	0.57846	0.945932	0.6978	1.02672	0.926136
2.46	3.648	gi|5031703	ras GTPase-activating protein-binding protein 1 [Homo sapiens]	1	0.92013	0.57087	0.85965	0.452881	0.880045	0.48158	0.95338	0.81138	0.917742	0.69749	0.95912	0.926288
4.52	7.756	gi|56549640	septin-2 [Homo sapiens]	3	0.99332	0.99483	1.23575	0.823806	1.353351	0.76378	1.72484	0.58133	1.284647	0.70863	0.93565	0.926332
1.68	5.263	gi|4758356	flap endonuclease 1 [Homo sapiens]	1	1.15011	0.39223	1.10884	0.675855	1.067793	0.6274	1.00338	0.98366	0.94599	0.67436	1.01114	0.929123
11.37	23.19	gi|4506725	40S ribosomal protein S4, X isoform X isoform [Homo sapiens]	5	0.97449	0.7355	0.92138	0.507416	0.964998	0.73662	1.12599	0.23149	0.953784	0.56743	1.00613	0.930998

6.34	23.31	gi|7305503	stomatin-like protein 2 [Homo sapiens]	5	1.17661	0.76853	1.01792	0.956275	0.970179	0.89195	1.06593	0.66163	0.877609	0.82736	1.03079	0.931181
10.33	10.26	gi|55770844	catenin alpha-1 [Homo sapiens]	6	1.03773	0.77724	1.42203	0.622809	1.56616	0.3328	1.23645	0.37736	0.997016	0.98129	1.04962	0.932653
9.63	46.12	gi|4507953	14-3-3 protein zeta/delta [Homo sapiens]	15	0.89929	0.60586	0.98527	0.856421	1.099533	0.64591	0.782	0.35808	0.893897	0.20654	1.01769	0.935496
4.47	42.89	gi|50592996	tubulin beta-3 chain isoform 1 [Homo sapiens]	29	0.78939	0.25372	1.16165	0.795319	0.842534	0.33496	0.84947	0.34915	0.973308	0.83089	1.03767	0.937168
6.89	5.112	gi|332688210	catenin delta-1 isoform 1AC [Homo sapiens]	4	1.02514	0.97096	0.55873	0.469676	1.062146	0.89209	1.20211	0.85366	0.898966	0.87101	0.97397	0.939865
6.98	58.88	gi|4507729	tubulin beta-2A chain [Homo sapiens]	41	0.948	0.76834	0.9999	0.99978	0.903984	0.61965	0.88799	0.79613	0.752115	0.29768	0.96998	0.940554
16.43	40.26	gi|24234747	interleukin enhancer-binding factor 2 isoform 1 [Homo sapiens]	13	1.0511	0.7347	0.91691	0.632643	0.969474	0.89625	0.95433	0.83549	0.978358	0.87143	0.98794	0.942942
9.34	25.2	gi|4505753	phosphoglycerate mutase 1 [Homo sapiens]	8	0.96526	0.90588	1.29984	0.237936	0.99391	0.9809	1.07441	0.81875	0.812698	0.54835	1.02478	0.944817
5.71	5.972	gi|15991831	hexokinase-1 isoform HKI-ta/tb [Homo sapiens]	4	1.10822	0.50056	1.13319	0.676442	1.011951	0.97474	1.03129	0.9428	0.989503	0.97605	0.98934	0.947689
3.41	14.19	gi|4506625	60S ribosomal protein L27a [Homo sapiens]	2	0.99323	0.96296	1.09852	0.728854	0.889413	0.73997	1.01465	0.96828	0.90663	0.53302	1.02292	0.950836
5.31	19.58	gi|4757908	calcyphosin isoform a [Homo sapiens]	4	0.7789	0.18217	1.03856	0.851888	0.898004	0.30411	0.85694	0.39045	0.957459	0.62455	0.98766	0.952912
3.08	4.58	gi|11386147	proactivator polypeptide isoform a preproprotein [Homo sapiens]	2	0.7416	0.68952	1.1469	0.767531	0.803347	0.76691	0.88676	0.90596	1.279128	0.79946	1.05125	0.953217

36.71	60.31	gi|4502107	annexin A5 [Homo sapiens]	28	1.02188	0.71923	1.04762	0.283796	1.041221	0.53751	1.02478	0.79402	1.003106	0.9381	1.00371	0.960815
5.52	11.11	gi|4758984	ras-related protein Rab-11A isoform 1 [Homo sapiens]	2	1.05913	0.823	0.92678	0.757553	1.219468	0.2448	1.02015	0.94044	0.980833	0.91924	1.01765	0.961985
7.02	9.559	gi|127139033	NADPH--cytochrome P450 reductase [Homo sapiens]	5	1.46665	0.26812	1.35078	0.78343	1.750845	0.28485	1.50179	0.58197	1.396446	0.19557	1.01903	0.963064
8.63	9.428	gi|304555583	elongation factor 1-delta isoform 1 [Homo sapiens]	4	1.05626	0.86542	1.13667	0.76818	1.069415	0.889	1.01608	0.96552	1.236347	0.55616	1.01694	0.964416
19.2	40	gi|4504245	histone H2A type 1-C [Homo sapiens]	42	1.03017	0.86464	0.91872	0.699991	0.933817	0.74092	1.19479	0.32328	0.974854	0.83978	0.99492	0.967249
25.54	15.89	gi|38202257	neutral alpha-glucosidase AB isoform 2 precursor [Homo sapiens]	14	1.04371	0.617	1.12201	0.3039	1.067465	0.42923	1.09288	0.44729	1.079871	0.33047	1.00402	0.967442
21.31	32.07	gi|5031753	heterogeneous nuclear ribonucleoprotein H [Homo sapiens]	18	0.97527	0.83475	0.82622	0.394135	1.084716	0.50783	0.92009	0.68814	0.934815	0.65578	0.99293	0.972627
27.69	17	gi|31621305	leucine-rich PPR motif-containing protein, mitochondrial precursor [Homo sapiens]	16	1.03366	0.87957	0.98367	0.912034	1.06177	0.80072	1.01847	0.89318	1.135167	0.43715	0.99557	0.974808
27.69	17	gi|31621305	leucine-rich PPR motif-containing protein, mitochondrial precursor [Homo sapiens]	16	1.03366	0.87957	0.98367	0.912034	1.06177	0.80072	1.01847	0.89318	1.135167	0.43715	0.99557	0.974808
26.86	80.9	gi|4507357	transgelin-2 [Homo sapiens]	44	0.94206	0.15285	0.98498	0.707045	0.982281	0.65704	0.95133	0.34752	0.973675	0.50977	0.99881	0.976356
4.24	33.33	gi|4506715	40S ribosomal protein S28 [Homo sapiens]	2	1.06887	0.59919	1.27592	0.261705	1.038603	0.6719	1.2457	0.32914	1.089153	0.60128	1.00461	0.986721

37.3	37.85	gi|24234688	stress-70 protein, mitochondrial precursor [Homo sapiens]	26	0.98254	0.76659	1.0399	0.634538	0.983587	0.83881	1.00545	0.95484	0.989735	0.89661	1.00057	0.990667
30.94	67.46	gi|18105048	histone H2B type 1-K [Homo sapiens]	47	1.08079	0.28917	0.9607	0.567832	0.967995	0.66044	1.16835	0.15734	1.040797	0.56995	0.99872	0.990732
16.64	14.34	gi|10863945	X-ray repair cross-complementing protein 5 [Homo sapiens]	8	1.01635	0.9172	0.98945	0.940811	1.036516	0.81601	0.87213	0.45505	1.077628	0.58733	0.99791	0.992423
2.67	8.974	gi|4826852	acyl carrier protein, mitochondrial precursor [Homo sapiens]	1	0.9768	0.97217	0.99151	0.99244	1.00298	0.99757	1.00566	0.99457	1.070932	0.8989	1.00317	0.993962
25.28	39.52	gi|14165435	heterogeneous nuclear ribonucleoprotein K isoform b [Homo sapiens]	15	0.95513	0.29475	0.95093	0.441387	0.970964	0.63696	0.97352	0.67837	1.055012	0.42691	1.00023	0.997159
4.86	20.75	gi|5803048	ER lumen protein retaining receptor 1 [Homo sapiens]	3	1.05895	0.89643	0.89579	0.811545	0.803206	0.31489	1.08902	0.89204	0.932645	0.86832	1.00017	0.999305
51.57	34.67	gi|74136883	heterogeneous nuclear ribonucleoprotein U isoform a [Homo sapiens]	34	0.71668		0.61704		0.608761		0.57835		0.824188		0.60273	
49.17	64.97	gi|57013276	tubulin alpha-1B chain [Homo sapiens]	63	0.93023		1.05083		0.983988		1.04234		0.83438		1.05425	
15.9	7.121	gi|4506787	ras GTPase-activating-like protein IQGAP1 [Homo sapiens]	9	0.81002		0.8467		1.040909		0.76514		1.094655		0.71155	
11.38	8.671	gi|5453998	importin-7 [Homo sapiens]	8												

9.52	11.28	gi|28178832	isocitrate dehydrogenase [NADP], mitochondrial precursor [Homo sapiens]	5	1.30054	0.97425	1.043614	0.95524	1.064548	1.35882
9.41	12.65	gi|285002233	glycerol-3-phosphate dehydrogenase, mitochondrial precursor [Homo sapiens]	8	0.63612	0.43177	0.605118	0.60499	0.806922	0.5378
9.02	44.56	gi|28395033	rho-related GTP-binding protein RhoC precursor [Homo sapiens]	6	1.92031	0.99363	1.700983	1.06466	1.078723	1.42801
8.89	4.503	gi|38201623	eukaryotic translation initiation factor 4 gamma 1 isoform 5 [Homo sapiens]	9	0.66325	1.16796	0.670461	0.73769	1.420639	0.48499
8.26	6.069	gi|4507943	exportin-1 [Homo sapiens]	4						
8	79.82	gi|4506669	60S acidic ribosomal protein P1 isoform 1 [Homo sapiens]	7	1.06983	0.97872	1.0065	1.03349	0.986462	0.95046
7.94	8.063	gi|45387945	extended synaptotagmin-2 [Homo sapiens]	4						
7.63	13.58	gi|4507353	TATA-binding protein-associated factor 2N isoform 2 [Homo sapiens]	5						
7.35	56.82	gi|11056061	thymosin beta-4 [Homo sapiens]	5	0.92823	1.03146	1.020059	0.8738	0.987755	1.09835

7.22	4.92	gi|4503509	eukaryotic translation initiation factor 3 subunit A [Homo sapiens]	5	2.39279	4.4105	2.463701	3.54179	2.01806	2.10314
6.78	7.069	gi|385298680	116 kDa U5 small nuclear ribonucleoprotein component isoform c [Homo sapiens]	6						
6.54	3.137	gi|40217847	U5 small nuclear ribonucleoprotein 200 kDa helicase [Homo sapiens]	6	1.14194	1.62901	0.86916	0.90805	1.30212	0.33775
6.25	4.864	gi|21361370	glycogen phosphorylase, brain form [Homo sapiens]	4	0.74647	0.88615	0.815465	0.71197	0.782035	1.25844
6.23	3.298	gi|54112117	splicing factor 3B subunit 1 isoform 1 [Homo sapiens]	3						
6.23	5.349	gi|71773329	annexin A6 isoform 1 [Homo sapiens]	3	4.50272	3.54499	4.594796	3.09253	3.811192	5.69826
6.15	5.579	gi|109148542	alanine--tRNA ligase, cytoplasmic [Homo sapiens]	3	0.82244	0.9845	0.950517	0.84237	0.686344	1.03448
6.15	20.49	gi|7706322	UPF0568 protein C14orf166 [Homo sapiens]	5						
6.09	25.17	gi|194097323	enoyl-CoA hydratase, mitochondrial [Homo sapiens]	7	2.03732	1.74468	2.134784	1.98122	1.267649	3.15589
6.08	28.65	gi|222352151	poly(rC)-binding protein 1 [Homo sapiens]	8	0.92485	0.81808	1.163378	1.00833	0.545206	1.00614
6.05	30.2	gi|50593002	U2 small nuclear ribonucleoprotein A' [Homo sapiens]	6	1.12271	0.91509	1.310521	1.0248	1.543845	1.43563

5.09	9.605	gi|4502643	T-complex protein 1 subunit zeta isoform a [Homo sapiens]	4	0.66609	0.49984	1.35727	1.1912	0.776804	0.82225
4.98	6.839	gi|45827806	atlastin-3 [Homo sapiens]	3						
4.97	6.071	gi|38327039	heat shock 70 kDa protein 4 [Homo sapiens]	3						
4.75	27.57	gi|6912238	peroxiredoxin-5, mitochondrial isoform a precursor [Homo sapiens]	5	0.7021	1.18907	1.478837	0.4313	1.39936	1.21253
4.67	7.559	gi|4758786	NADH dehydrogenase [ubiquinone] iron-sulfur protein 2, mitochondrial isoform 1 precursor [Homo sapiens]	2						
4.63	6.966	gi|18677735	protein PRRC1 [Homo sapiens]	3						
4.5	14.94	gi|23110942	proteasome subunit alpha type-5 isoform 1 [Homo sapiens]	3						
4.46	12.4	gi|269914128	OCIA domain-containing protein 1 isoform 4 [Homo sapiens]	2	1.21801	1.37961	1.346004	1.48184	1.117946	1.18723
4.44	10.44	gi|4502049	aldose reductase [Homo sapiens]	4	1.47454	1.36634	1.409654	0.99615	0.923845	1.00292
4.41	9.053	gi|5453595	adenylyl cyclase-associated protein 1 [Homo sapiens]	4	1.57866	1.2196	2.380994	1.61705	1.994657	2.52747
4.4	5.615	gi|4506341	ATP-binding cassette sub-family D member 3 isoform a [Homo sapiens]	3	0.32324	0.75219	1.198383	1.30105	1.203778	1.54799

4.39	3.725	gi|6005757	FACT complex subunit SPT16 [Homo sapiens]	2						
4.37	6.442	gi|56788366	phosphatidylinositol-binding clathrin assembly protein isoform 1 [Homo sapiens]	3	0.84523	0.80269	1.0253	0.9725	0.986006	0.97061
4.32	6.795	gi|308818195	dihydropyrimidinase-related protein 2 isoform 1 [Homo sapiens]	2						
4.29	6.369	gi|4505891	procollagen-lysine,2-oxoglutarate 5-dioxygenase 3 precursor [Homo sapiens]	5						
4.17	1.681	gi|153946395	tenascin precursor [Homo sapiens]	2						
4.17	5.479	gi|319655561	alpha-aminoadipic semialdehyde dehydrogenase isoform 2 [Homo sapiens]	3						
4.14	3.894	gi|17999541	vacuolar protein sorting-associated protein 35 [Homo sapiens]	3						
4.13	6.225	gi|116805340	glycine--tRNA ligase precursor [Homo sapiens]	2	0.80401	0.7306	0.752112	0.67938	0.6988	0.817
4.12	5.368	gi|302344762	non-specific lipid-transfer protein isoform 6 proprotein [Homo sapiens]	2						

4.11	2.758	gi|148536855	coatomer subunit alpha isoform 1 [Homo sapiens]	3	0.84495	1.14378	0.879331	1.20518	0.78377	0.92041
4.11	12.28	gi|53692187	actin-related protein 2 isoform a [Homo sapiens]	3						
4.11	6.528	gi|5803187	transaldolase [Homo sapiens]	3						
4.11	6.742	gi|6598323	rab GDP dissociation inhibitor beta isoform 1 [Homo sapiens]	2	1.31164	1.35366	1.719467	1.22879	1.907645	1.82516
4.11	17.34	gi|5454090	translocon-associated protein subunit delta isoform 2 precursor [Homo sapiens]	2	1.09614	1.01594	1.240244	1.2115	1.02137	1.06552
4.1	2.276	gi|21361794	cullin-associated NEDD8-dissociated protein 1 [Homo sapiens]	3						
4.1	2.816	gi|38569421	ATP-citrate synthase isoform 1 [Homo sapiens]	2	1.47681	1.78721	2.177375	1.43963	1.056422	1.85165
4.1	12.5	gi|19923315	serine hydroxymethyltransfe rase, mitochondrial isoform 1 precursor [Homo sapiens]	5	2.17269	1.45205	1.733907	1.84592	1.6707	1.17943
4.09	3.904	gi|109134349	coatomer subunit gamma-2 [Homo sapiens]	2						
4.08	7.196	gi|195539395	26S protease regulatory subunit 10B [Homo sapiens]	2	0.78017	0.46331	1.391888	1.14351	0.599761	1.27963

4.03	14.64	gi|30410792	proteasome activator complex subunit 2 [Homo sapiens]	3	1.35076	1.30523	0.949045	1.36713	1.264915	0.92517
4.02	8.119	gi|166795301	prenylcysteine oxidase 1 precursor [Homo sapiens]	3	1.05175	0.98525	1.0761	0.91641	0.986455	0.77613
4.02	4.662	gi|8922712	septin-11 [Homo sapiens]	2						
4.01	5.568	gi|37537716	eukaryotic translation initiation factor 5 [Homo sapiens]	3						
4.01	29.13	gi|17933772	protein S100-A16 [Homo sapiens]	2	0.91551	0.57837	1.177884	1.32834	1.245144	1.04009
4	64.97	gi|393715091	tubulin alpha-1A chain isoform 1 [Homo sapiens]	61	0.86763	1.09195	1.091364	0.95509	0.999499	1.17204
4	17.76	gi|151108473	mitochondrial fission 1 protein [Homo sapiens]	2						
4	41.07	gi|7705501	transmembrane protein 14C [Homo sapiens]	2						
3.91	27.97	gi|21956645	myotrophin [Homo sapiens]	3						
3.85	4.626	gi|25777602	26S proteasome non-ATPase regulatory subunit 2 [Homo sapiens]	3						
3.84	1.638	gi|110611220	ribosome-binding protein 1 [Homo sapiens]	2						
3.68	4.777	gi|4758648	kinesin-1 heavy chain [Homo sapiens]	4						
3.6	19.44	gi|4759212	tubulin-specific chaperone A [Homo sapiens]	4	0.72985	0.79919	0.8261	0.89374	0.765348	0.87837

3.53	6.383	gi|4501993	alkyldihydroxyacetonephosphate synthase, peroxisomal precursor [Homo sapiens]	3	1.3973	1.20706	1.229661	1.30968	0.870439	0.9839
3.47	6.713	gi|9951915	adenosylhomocysteinase isoform 1 [Homo sapiens]	3	0.91699	1.54572	0.960423	0.51959	1.187545	1.25412
3.47	11.58	gi|397138571	PREDICTED: putative trypsin-6 isoform 2 [Homo sapiens]	6	0.6452	0.71299	0.688758	0.84167	0.871762	0.4976
3.42	5.369	gi|4503971	rab GDP dissociation inhibitor alpha [Homo sapiens]	2						
3.41	3.436	gi|56676330	heterochromatin protein 1-binding protein 3 [Homo sapiens]	3	0.93177	1.38266	0.985569	0.63015	1.159698	0.87353
3.39	16.67	gi|28933465	syntaxin-12 [Homo sapiens]	3	0.65228	1.28782	1.08027	0.56742	0.870332	1.17819
3.35	8.754	gi|14591909	60S ribosomal protein L5 [Homo sapiens]	2	0.96889	1.29402	0.971669	1.24532	1.17296	0.77783
3.33	3.212	gi|45827771	enhancer of mRNA-decapping protein 4 [Homo sapiens]	2						
3.33	6.25	gi|38150007	small nuclear ribonucleoprotein-associated proteins B and B' isoform B' [Homo sapiens]	2	0.94294	0.69364	0.869764	0.63095	0.803134	0.49355
3.32	1.404	gi|148529014	DNA damage-binding protein 1 [Homo sapiens]	2						

3.27	10.32	gi|4503139	cathepsin B preproprotein [Homo sapiens]	2	0.70471	0.80279	0.654314	0.69681	0.634219	0.7471
3.25	11.01	gi|346644849	DNA-(apurinic or apyrimidinic site) lyase [Homo sapiens]	3	1.2326	1.04638	1.573288	1.95319	1.46752	1.26419
3.15	13.95	gi|7705696	thioredoxin domain-containing protein 12 precursor [Homo sapiens]	2						
3.06	9.44	gi|6005721	erlin-2 isoform 1 [Homo sapiens]	3	1.05746	1.13755	0.971272	1.06971	0.825092	1.21269
2.96	3.714	gi|157389005	calpain-2 catalytic subunit isoform 1 [Homo sapiens]	2						
2.93	20.61	gi|5802966	destrin isoform a [Homo sapiens]	3	0.86667	1.08644	1.071251	0.54751	0.656449	0.63406
2.93	6.395	gi|217272851	prolyl 4-hydroxylase subunit alpha-1 isoform 3 precursor [Homo sapiens]	2	1.05682	0.51686	1.175281	1.00522	0.697477	1.48189
2.85	0.8478	gi|194595509	spectrin alpha chain, brain isoform 1 [Homo sapiens]	2						
2.85	9.562	gi|5454088	acidic leucine-rich nuclear phosphoprotein 32 family member B [Homo sapiens]	2						
2.81	18.62	gi|7657257	mitochondrial import receptor subunit TOM20 homolog [Homo sapiens]	2	1.12647	0.96503	0.830369	1.03807	0.875227	0.89786
2.71	9.603	gi|386781571	radixin isoform 1 [Homo sapiens]	6	0.81532	1.25499	1.601472	0.54108	0.565041	1.1877

2.67	9.375	gi|42761474	CD59 glycoprotein preproprotein [Homo sapiens]	1	0.86805	0.97073	0.997505	1.10319	0.866608	0.84686
2.65	1.65	gi|9257257	WD repeat-containing protein 1 isoform 1 [Homo sapiens]	1						
2.64	7.125	gi|365192532	myosin-10 isoform 1 [Homo sapiens]	15	0.66064	0.65413	1.10042	0.93428	0.603853	0.98694
2.64	5.534	gi|219283152	apoptosis inhibitor 5 isoform a [Homo sapiens]	4	0.85311	1.16414	1.634897	1.38697	1.133337	1.00298
2.61	7.116	gi|30410796	proteasome activator complex subunit 3 isoform 2 [Homo sapiens]	3	0.63085	0.92394	0.72022	0.36527	0.9777	0.76639
2.61	12.16	gi|4507231	single-stranded DNA-binding protein, mitochondrial precursor [Homo sapiens]	3	1.58337	1.341	1.461128	1.51503	1.563343	1.89598
2.6	7.182	gi|7657441	28 kDa heat- and acid-stable phosphoprotein [Homo sapiens]	1	0.85503	0.87906	0.343909	0.40988	0.740731	1.13019
2.58	10.82	gi|13569879	acidic leucine-rich nuclear phosphoprotein 32 family member E isoform 1 [Homo sapiens]	3						
2.51	1.262	gi|283436220	ATPase family AAA domain-containing protein 3A isoform 1 [Homo sapiens]	1	0.57696	0.62027	0.424078	0.20236	0.411318	0.31566
2.49	2.839	gi|4885409	vigilin isoform a [Homo sapiens]	2						

2.45	5.919	gi|12056465	rRNA 2'-O-methyltransferase fibrillarin [Homo sapiens]	2	0.84699	1.04848	0.77802	0.72476	0.814099	1.04849
2.43	7.67	gi|312283701	malate dehydrogenase, cytoplasmic isoform 1 [Homo sapiens]	2	0.88475	0.75548	0.903711	0.51043	0.991972	0.72992
2.42	16.39	gi|21040371	ATP-dependent RNA helicase DDX39A [Homo sapiens]	5						
2.42	9.394	gi|19923181	PDZ and LIM domain protein 4 isoform 1 [Homo sapiens]	3	0.74782	1.03132	1.006054	0.97864	1.449987	1.22062
2.4	32.52	gi|4507949	14-3-3 protein beta/alpha [Homo sapiens]	14	1.25318	1.06152	1.807213	1.40418	0.817041	1.31435
2.38	1.307	gi|11225260	DNA topoisomerase 1 [Homo sapiens]	1	0.66957	0.99773	1.318882	0.92511	1.109218	1.38873
2.36	5.966	gi|7657649	tropomodulin-3 [Homo sapiens]	1	0.54916	0.58171	0.468889	0.03686	0.571124	0.66021
2.35	14.81	gi|4505357	NADH dehydrogenase [ubiquinone] 1 alpha subcomplex subunit 4 [Homo sapiens]	1	1.14039	0.96507	1.457507	0.9558	1.061194	1.20029
2.34	1.223	gi|124494238	unconventional myosin-Ic isoform a [Homo sapiens]	1	0.62802	0.55643	0.50495	0.18695	0.410643	0.63169
2.33	1.135	gi|30581135	structural maintenance of chromosomes protein 1A [Homo sapiens]	2						

2.33	2.57	gi|21536326	heterogeneous nuclear ribonucleoprotein U-like protein 1 isoform a [Homo sapiens]	1	0.27416	0.26981	0.297418	0.21473	0.517851	0.40407
2.32	4.837	gi|38202214	protein transport protein Sec23A [Homo sapiens]	2						
2.31	1.844	gi|10190742	ubiquitin carboxyl-terminal hydrolase 29 [Homo sapiens]	1						
2.3	12.24	gi|66737374	protein S100-A13 [Homo sapiens]	1	1.52857	0.65298	0.87031	1.73263	1.020324	1.2015
2.29	10.38	gi|373432684	ubiquitin-conjugating enzyme E2 L3 isoform 4 [Homo sapiens]	3						
2.29	6.407	gi|4759034	eukaryotic peptide chain release factor subunit 1 [Homo sapiens]	2	0.79436	0.80778	0.666296	0.44907	0.246555	0.56426
2.28	4.723	gi|316983160	NADH-ubiquinone oxidoreductase 75 kDa subunit, mitochondrial isoform 5 [Homo sapiens]	2						
2.27	23.2	gi|4502203	ADP-ribosylation factor 3 [Homo sapiens]	4	1.20672	1.21644	1.051839	1.28095	0.886377	1.39553
2.26	1.534	gi|33356174	pinin [Homo sapiens]	1	0.85067	0.83291	1.13135	0.86094	1.075418	0.5121
2.26	4.172	gi|33469968	DNA replication licensing factor MCM7 isoform 1 [Homo sapiens]	2	0.66264	0.98625	1.075919	0.92704	0.936778	0.82464

2.25	1.436	gi|296317244	extended synaptotagmin-1 isoform 1 [Homo sapiens]	2						
2.25	13.1	gi|4506687	40S ribosomal protein S15 [Homo sapiens]	2	0.72722	0.74515	0.691769	0.38782	0.804124	0.74194
2.25	7.981	gi|4502303	ATP synthase subunit O, mitochondrial precursor [Homo sapiens]	2	1.18023	1.17369	0.871478	0.88042	0.799421	0.82527
2.25	10.62	gi|31563374	eukaryotic translation initiation factor 6 isoform c [Homo sapiens]	1						
2.24	1.957	gi|11863154	coatomer subunit delta isoform 1 [Homo sapiens]	1						
2.24	1.579	gi|51944971	nodal modulator 2 isoform 1 precursor [Homo sapiens]	1						
2.24	1.062	gi|21237808	SWI/SNF complex subunit SMARCC2 isoform b [Homo sapiens]	1						
2.24	4.889	gi|7661922	ras-related protein Rab-21 [Homo sapiens]	1	1.2084	0.76928	1.115088	0.71112	0.929048	0.73102
2.23	0.9009	gi|27477134	nuclear pore membrane glycoprotein 210 precursor [Homo sapiens]	1						
2.23	1.794	gi|24797086	importin-5 [Homo sapiens]	1	1.02307	1.20567	1.213528	1.21964	0.937203	0.83983

2.22	1.463	gi|61742777	leucyl-cystinyl aminopeptidase isoform 1 [Homo sapiens]	1						
2.22	2.968	gi|4826960	glutamine--tRNA ligase [Homo sapiens]	2						
2.21	1.286	gi|31377697	procollagen galactosyltransferase 1 precursor [Homo sapiens]	1	0.89469	0.93139	1.864245	1.28507	2.11273	1.20325
2.2	1.372	gi|19924129	DNA repair protein RAD50 [Homo sapiens]	1						
2.19	2.111	gi|14043022	methionine--tRNA ligase, cytoplasmic [Homo sapiens]	1						
2.18	5.882	gi|4506623	60S ribosomal protein L27 [Homo sapiens]	1	1.10035	0.91496	0.965733	1.13191	0.864173	1.03142
2.17	1.894	gi|6912482	LETM1 and EF-hand domain-containing protein 1, mitochondrial precursor [Homo sapiens]	1	0.71105	0.92143	1.40973	1.03453	0.92632	0.9231
2.17	1.799	gi|4504373	beta-hexosaminidase subunit beta preproprotein [Homo sapiens]	1						
2.16	0.8333	gi|21264365	nuclear pore complex protein Nup98-Nup96 isoform 1 [Homo sapiens]	1						

2.16	0.65	gi|52630326	chromodomain-helicase-DNA-binding protein 3 isoform 1 [Homo sapiens]	1						
2.16	7.489	gi|38505222	protein disulfide-isomerase TMX3 precursor [Homo sapiens]	3	0.79121	0.71153	0.996528	0.97663	0.65292	0.96825
2.16	6.297	gi|4504511	dnaJ homolog subfamily A member 1 [Homo sapiens]	1	0.7664	1.08557	0.631588	0.45396	0.520625	0.50215
2.15	0.742	gi|21040326	protein SON isoform F [Homo sapiens]	2	0.50701	0.5684	0.514403	1.07732	0.679063	1.01611
2.15	1.086	gi|112382237	synemin isoform A [Homo sapiens]	1						
2.15	0.8696	gi|22202611	carboxypeptidase D isoform 1 precursor [Homo sapiens]	1	0.98697	0.81086	1.696009	1.31237	1.248185	1.63565
2.15	1.732	gi|17149828	N-alpha-acetyltransferase 15, NatA auxiliary subunit [Homo sapiens]	1						
2.15	0.7216	gi|254939537	unconventional myosin-XIX isoform 2 [Homo sapiens]	1						
2.15	1.514	gi|333033787	nascent polypeptide-associated complex subunit alpha isoform a [Homo sapiens]	1	1.09531	0.98972	1.223688	0.95587	1.039843	0.96309

2.15	17.17	gi|27436901	39S ribosomal protein L12, mitochondrial [Homo sapiens]	2	1.43183	1.09845	1.298743	1.5196	0.923348	0.5195
2.15	3.024	gi|5730023	ruvB-like 2 [Homo sapiens]	1						
2.15	3.024	gi|5730023	ruvB-like 2 [Homo sapiens]	1						
2.14	5.155	gi|4505119	methyl-CpG-binding domain protein 3 [Homo sapiens]	1						
2.14	16.38	gi|7524346	adenylate kinase 2, mitochondrial isoform b [Homo sapiens]	4	1.3076	1.68282	1.177596	1.40287	1.297324	1.02061
2.12	4.242	gi|30089972	peroxisomal acyl-coenzyme A oxidase 1 isoform a [Homo sapiens]	3	0.68112	0.64966	1.304939	0.76658	0.415005	1.11262
2.12	0.7937	gi|62241042	bifunctional glutamate/proline--tRNA ligase [Homo sapiens]	1						
2.12	3.109	gi|37594471	3-hydroxyisobutyryl-CoA hydrolase, mitochondrial isoform 1 precursor [Homo sapiens]	1	1.03883	1.04724	0.897887	0.7685	0.744229	0.95015
2.12	0.8649	gi|7662180	centrosomal protein of 104 kDa [Homo sapiens]	1	0.93559	0.78752	0.838642	0.90171	0.888156	0.80893
2.12	8.264	gi|4826964	UV excision repair protein RAD23 homolog A isoform 1 [Homo sapiens]	1						

2.12	3.587	gi|237820620	glutamate-rich WD repeat-containing protein 1 [Homo sapiens]	1							
2.11	5.419	gi|5453629	dynactin subunit 2 isoform 1 [Homo sapiens]	2	1.03997	0.76595	1.000128	0.98143	0.874996	1.13523	
2.11	6.579	gi|7706497	UMP-CMP kinase isoform a [Homo sapiens]	2	0.84369	0.62042	1.25376	1.12914	1.027527	0.9796	
2.11	12.78	gi|5901926	cleavage and polyadenylation specificity factor subunit 5 [Homo sapiens]	1	1.40339	1.13078	2.007566	1.89092	1.45933	1.30963	
2.1	2.178	gi|4506103	interferon-induced, double-stranded RNA-activated protein kinase isoform a [Homo sapiens]	1							
2.1	16.81	gi|5902102	small nuclear ribonucleoprotein Sm D1 [Homo sapiens]	1	1.06963	1.11104	1.238307	1.14907	0.987501	1.01882	
2.09	10.23	gi|153791352	POTE ankyrin domain family member F [Homo sapiens]	45							
2.09	1.441	gi|27477136	zinc finger CCCH-type antiviral protein 1 isoform 1 [Homo sapiens]	1							
2.09	5.329	gi|45827712	protein quaking isoform HQK-7B [Homo sapiens]	2							

2.08	7.322	gi|5031631	lysosome membrane protein 2 isoform 1 precursor [Homo sapiens]	2						
2.08	7.717	gi|21389315	tricarboxylate transport protein, mitochondrial isoform a precursor [Homo sapiens]	1						
2.08	2.143	gi|4826870	nucleobindin-2 precursor [Homo sapiens]	1						
2.08	6.186	gi|223555917	protein LYRIC [Homo sapiens]	1						
2.08	4.658	gi|14719402	ribosome biogenesis regulatory protein homolog [Homo sapiens]	1	1.01198	0.95753	0.794942	1.12435	1.176603	0.75437
2.08	3.464	gi|255958289	perilipin-3 isoform 2 [Homo sapiens]	1						
2.08	10.11	gi|4506203	proteasome subunit beta type-7 proprotein [Homo sapiens]	2						
2.07	15.66	gi|4826760	heterogeneous nuclear ribonucleoprotein F [Homo sapiens]	10						
2.07	63.64	gi|10863895	thymosin beta-10 [Homo sapiens]	4	0.95092	1.06945	1.129401	0.97323	1.04227	1.02178
2.07	1.448	gi|133925811	transportin-1 isoform 1 [Homo sapiens]	1						
2.07	2.82	gi|20070228	nucleobindin-1 precursor [Homo sapiens]	1						

2.07	3.761	gi|42542379	serine/arginine repetitive matrix protein 1 [Homo sapiens]	2						
2.07	10.68	gi|83641885	transcription factor BTF3 isoform A [Homo sapiens]	2	0.84445	0.94586	0.965018	1.10656	0.886324	1.11327
2.07	5.515	gi|17402904	exosome complex component MTR3 [Homo sapiens]	1	0.73338	0.7553	0.937293	1.04302	0.94509	1.08138
2.06	3.664	gi|117938759	protein ALEX XLas [Homo sapiens]	2	0.79719	1.3224	0.93709	1.06557	1.270463	1.04841
2.06	0.7087	gi|57164975	treacle protein isoform b [Homo sapiens]	1	0.78893	0.91721	0.515189	0.61893	0.519783	0.68702
2.06	10.12	gi|291621647	membrane-associated progesterone receptor component 2 [Homo sapiens]	2	1.41716	1.55355	1.276417	1.66223	1.337127	1.38874
2.06	3.593	gi|284448551	NADH-cytochrome b5 reductase 3 isoform 3 [Homo sapiens]	1	0.67991	0.59962	1.002967	0.87319	0.970829	0.83486
2.06	2.756	gi|4758340	phenylalanine--tRNA ligase alpha subunit [Homo sapiens]	1						
2.06	1.923	gi|48762920	6-phosphofructokinase, liver type [Homo sapiens]	1						
2.06	2.917	gi|19913424	V-type proton ATPase catalytic subunit A [Homo sapiens]	1						

2.06	6.557	gi|13376840	WD repeat-containing protein 61 [Homo sapiens]	1	1.08326	0.77932	0.993702	1.19976	0.904646	0.81726
2.06	4.157	gi|109452587	protein FAM98B isoform 1 [Homo sapiens]	1	0.92196	1.00054	1.440914	1.02121	1.427297	0.86005
2.06	20.33	gi|212276121	malignant T-cell-amplified sequence 1 isoform 2 [Homo sapiens]	2						
2.05	1.351	gi|217416374	coiled-coil domain-containing protein 164 [Homo sapiens]	1						
2.05	2.848	gi|83700233	eukaryotic translation initiation factor 3 subunit C [Homo sapiens]	1						
2.05	2.116	gi|123173757	ribonucleoprotein PTB-binding 1 [Homo sapiens]	1						
2.05	1.729	gi|21361659	importin-9 [Homo sapiens]	1						
2.05	7.317	gi|371872778	transmembrane and coiled-coil domain-containing protein 1 isoform b [Homo sapiens]	1	1.24359	1.33452	1.106128	0.63591	1.539528	0.99547
2.05	5.414	gi|13027602	DDRGK domain-containing protein 1 precursor [Homo sapiens]	1						
2.05	2.146	gi|148596949	nucleolar and coiled-body phosphoprotein 1 [Homo sapiens]	1	0.95672	0.96839	0.740136	1.56721	0.678062	0.57317
2.05	8.072	gi|209969700	coiled-coil domain-containing protein 124 [Homo sapiens]	2						

2.05	3.07	gi|4506753	ruvB-like 1 [Homo sapiens]	1						
2.05	8.974	gi|4506181	proteasome subunit alpha type-2 [Homo sapiens]	2	1.01666	1.14002	0.840779	0.73922	0.93359	0.91044
2.05	6	gi|83921614	cytochrome b5 type B [Homo sapiens]	1	1.75772	4.65792	2.081941	1.80761	1.731172	1.11116
2.04	1.815	gi|150378533	ubiquitin carboxyl-terminal hydrolase 7 [Homo sapiens]	1						
2.04	3.695	gi|4506209	26S protease regulatory subunit 7 isoform 1 [Homo sapiens]	1	0.55722	1.89039	2.196407	2.30166	1.325583	1.15757
2.04	6	gi|42491362	inhibitor of nuclear factor kappa-B kinase-interacting protein isoform 2 [Homo sapiens]	1	1.33451	0.97453	0.847668	1.18622	1.052938	0.72269
2.04	2.044	gi|21070997	stromal interaction molecule 1 precursor [Homo sapiens]	1						
2.04	3.795	gi|81295407	acyl-coenzyme A thioesterase 9, mitochondrial isoform a precursor [Homo sapiens]	1						
2.04	9.302	gi|215490089	histone deacetylase complex subunit SAP18 [Homo sapiens]	1						
2.04	4.984	gi|16445421	secretory carrier-associated membrane protein 3 isoform 2 [Homo sapiens]	1						

2.04	16.13	gi|13775198	SH3 domain-binding glutamic acid-rich-like protein 3 [Homo sapiens]	1	1.17325	1.93406	1.533712	0.74937	2.796483	1.02523
2.04	5.556	gi|4885413	histidine triad nucleotide-binding protein 1 [Homo sapiens]	1	1.08696	1.08068	1.320304	1.09545	0.955904	1.13904
2.03	0.8224	gi|12083581	1-phosphatidylinositol 4,5-bisphosphate phosphodiesterase beta-1 isoform a [Homo sapiens]	1						
2.03	6.912	gi|24308295	grpE protein homolog 1, mitochondrial precursor [Homo sapiens]	2						
2.03	2.493	gi|47458820	signal transducer and activator of transcription 3 isoform 3 [Homo sapiens]	1						
2.03	2.513	gi|33457336	interferon regulatory factor 2-binding protein-like [Homo sapiens]	1	0.85583	0.76036	1.050461	0.80003	0.814673	0.70424
2.03	1.183	gi|112789528	cohesin subunit SA-2 isoform a [Homo sapiens]	1	0.43056	0.45479	0.738483	0.83165	0.690343	0.78841
2.03	2.108	gi|156416003	succinate dehydrogenase [ubiquinone] flavoprotein subunit, mitochondrial [Homo sapiens]	1	1.91777	2.26216	2.547209	1.10328	2.323811	1.762

2.03	1.116	gi|375298678	tripartite motif-containing protein 46 isoform 3 [Homo sapiens]	1						
2.03	7.303	gi|5031597	actin-related protein 2/3 complex subunit 3 [Homo sapiens]	1	1.13189	1.17068	1.715736	1.03642	0.949944	1.34422
2.03	4.286	gi|156631005	26S proteasome non-ATPase regulatory subunit 8 [Homo sapiens]	1						
2.03	1.783	gi|20127450	protein kinase C beta type isoform 2 [Homo sapiens]	1						
2.03	4.306	gi|5729991	26S protease regulatory subunit 6B isoform 1 [Homo sapiens]	1						
2.03	4.375	gi|46593007	cytochrome b-c1 complex subunit 1, mitochondrial precursor [Homo sapiens]	2						
2.03	5.389	gi|156416000	ubiquitin-fold modifier-conjugating enzyme 1 [Homo sapiens]	1	1.02711	0.9376	1.189223	1.25373	0.952967	1.2758
2.02	2.243	gi|164519136	endothelin-converting enzyme 1 isoform 3 [Homo sapiens]	1	0.46947	1.08796	0.734908	0.60167	0.984969	0.70464
2.02	2.919	gi|27262628	nuclear autoantigenic sperm protein isoform 2 [Homo sapiens]	1	1.17216	1.29347	1.231107	1.42517	0.964626	1.11173

2.02	6.757	gi|8922331	protein mago nashi homolog 2 [Homo sapiens]	1	0.96856	0.79949	0.987411	0.42568	0.737719	0.60271
2.02	1.11	gi|82799484	cAMP-specific 3',5'-cyclic phosphodiesterase 4B isoform 3 [Homo sapiens]	1						
2.02	6.667	gi|23065552	glutathione S-transferase Mu 3 [Homo sapiens]	1						
2.02	3.957	gi|42476108	neuronal membrane glycoprotein M6-a isoform 1 [Homo sapiens]	1	0.96611	0.86918	0.528097	0.77639	0.964402	0.7135
2.02	7.282	gi|4502419	flavin reductase (NADPH) [Homo sapiens]	1	0.82492	0.76531	0.45059	0.59814	0.256164	0.86739
2.02	7.627	gi|4759158	small nuclear ribonucleoprotein Sm D2 isoform 1 [Homo sapiens]	1	0.81977	0.97189	0.852765	1.01776	0.888338	1.01979
2.02	10.87	gi|118402586	lactoylglutathione lyase [Homo sapiens]	1						
2.01	12.95	gi|31543397	phosphoglycerate kinase 2 [Homo sapiens]	6						
2.01	4.326	gi|40538799	ubiquilin-4 [Homo sapiens]	2	1.18846	1.60339	0.711944	1.67087	1.463316	1.57243
2.01	2.414	gi|262359914	excitatory amino acid transporter 1 isoform 2 [Homo sapiens]	2	1.73275	2.19773	1.971571	2.32741	1.602276	1.20143
2.01	3.667	gi|5031599	actin-related protein 2/3 complex subunit 2 [Homo sapiens]	1	2.27054	1.63589	2.338493	1.29921	0.955917	0.99613

2.01	4.645	gi|4506387	UV excision repair protein RAD23 homolog B isoform 1 [Homo sapiens]	2						
2.01	2.193	gi|48526509	mitochondrial import inner membrane translocase subunit TIM50 [Homo sapiens]	1						
2.01	7.339	gi|10801345	eukaryotic translation initiation factor 3 subunit K [Homo sapiens]	2						
2.01	5.859	gi|21361565	ATP synthase subunit b, mitochondrial precursor [Homo sapiens]	1						
2.01	9.434	gi|4506629	60S ribosomal protein L29 [Homo sapiens]	1	1.17387	1.89628	1.432563	1.65658	1.126862	1.46405
2.01	11.5	gi|5032133	eukaryotic translation initiation factor 1 [Homo sapiens]	1						
2	21.54	gi|4507951	14-3-3 protein eta [Homo sapiens]	10						
2	5.545	gi|109240550	paraspeckle component 1 [Homo sapiens]	3	0.87887	1.04839	0.999175	0.77477	0.444983	0.87935
2	9.79	gi|5453599	F-actin-capping protein subunit alpha-2 [Homo sapiens]	2	1.68511	1.74422	1.030252	0.81911	1.008209	0.92859
2	11.84	gi|8923557	protein C20orf11 [Homo sapiens]	2						

2	6.806	gi|4504001	gap junction alpha-1 protein [Homo sapiens]	2						
2	1.751	gi|16306548	serine--tRNA ligase, cytoplasmic [Homo sapiens]	1						
2	4.595	gi|40806190	TMEM189-UBE2V1 fusion protein [Homo sapiens]	2	1.07323	0.77767	0.860442	0.77759	0.910226	1.2448
2	2.063	gi|32307144	procollagen-lysine,2-oxoglutarate 5-dioxygenase 1 precursor [Homo sapiens]	2						
2	4.665	gi|31377663	armadillo repeat-containing protein 10 isoform a [Homo sapiens]	1						
2	8.78	gi|22538465	proteasome subunit beta type-3 [Homo sapiens]	2						
2	3.169	gi|116256489	septin-9 isoform c [Homo sapiens]	1						
2	3.409	gi|24430151	26S protease regulatory subunit 4 [Homo sapiens]	2						
2	2.318	gi|14589889	cadherin-2 preproprotein [Homo sapiens]	1						
2	8.553	gi|4505893	proteolipid protein 2 [Homo sapiens]	1	1.02496	0.98235	1.004735	1.11342	0.972551	1.01897
2	16.76	gi|13376717	optic atrophy 3 protein isoform b [Homo sapiens]	1						
2	2.195	gi|112789562	gamma-interferon-inducible protein 16 isoform 2 [Homo sapiens]	1	1.037	0.5475	0.795671	0.88866	0.763422	0.71412

2	4.583	gi|149193321	G-rich sequence factor 1 isoform 1 [Homo sapiens]	1						
2	3.788	gi|58761500	obg-like ATPase 1 isoform 1 [Homo sapiens]	2						
2	10.14	gi|7705636	vesicle transport protein GOT1B [Homo sapiens]	1	6.49616	2.27653	4.414598	4.37636	2.95415	3.01594
2	5.234	gi|4506491	replication factor C subunit 4 [Homo sapiens]	1						
2	9.418	gi|5174723	mitochondrial import receptor subunit TOM40 homolog [Homo sapiens]	2						
2	3.65	gi|21264355	SWI/SNF-related matrix-associated actin-dependent regulator of chromatin subfamily E member 1 [Homo sapiens]	1						
2	11.96	gi|4507129	small nuclear ribonucleoprotein E [Homo sapiens]	3	0.9772	0.90978	1.049372	1.20251	1.077493	0.90789
2	7.246	gi|70608174	tumor protein D52 isoform 2 [Homo sapiens]	1						
2	12.82	gi|13491174	MARCKS-related protein [Homo sapiens]	2	1.18191	1.19468	1.390571	1.32367	1.278538	1.18775
2	9.028	gi|260763955	NADH dehydrogenase [ubiquinone] 1 alpha subcomplex subunit 13 [Homo sapiens]	1						

2	4.016	gi|222080062	NADH dehydrogenase [ubiquinone] flavoprotein 2, mitochondrial precursor [Homo sapiens]	1						
2	6.771	gi|93277094	protein FAM210B [Homo sapiens]	1	1.49494	1.14174	2.094259	3.25341	2.226591	2.03697
2	4.864	gi|217035102	cleavage and polyadenylation specificity factor subunit 7 isoform 1 [Homo sapiens]	1						
2	10.56	gi|206725530	peptidyl-prolyl cis-trans isomerase FKBP2 precursor [Homo sapiens]	1						
2	15.79	gi|4507133	small nuclear ribonucleoprotein G [Homo sapiens]	1						
2	6.18	gi|6005794	PRA1 family protein 2 [Homo sapiens]	1	1.1698	1.31058	1.179651	1.26741	1.467394	1.16345
2	7.568	gi|6005884	translocon-associated protein subunit gamma [Homo sapiens]	1	1.20281	1.13479	1.173929	1.2955	0.868648	0.98349
2	14.74	gi|11024700	mitochondrial import inner membrane translocase subunit Tim13 [Homo sapiens]	4						
2	10.11	gi|5453704	PRA1 family protein 3 [Homo sapiens]	1	2.88799	0.96158	2.671536	0.63546	1.864812	1.05061
2	7.254	gi|4507605	CD70 antigen [Homo sapiens]	1						

2	3.175	gi|5870893	sodium-coupled neutral amino acid transporter 3 [Homo sapiens]	1	0.79604	1.03163	0.501218	0.83569	0.968156	0.28512
2	3.686	gi|25470886	DAZ-associated protein 1 isoform b [Homo sapiens]	1						
2	5.24	gi|282165814	transmembrane emp24 domain-containing protein 5 isoform 1 precursor [Homo sapiens]	1						
2	8.553	gi|4758714	microsomal glutathione S-transferase 3 [Homo sapiens]	2	1.07214	0.77882	1.157946	0.94076	1.049032	1.0345
2	15.23	gi|5453678	epididymal secretory protein E1 precursor [Homo sapiens]	1						
2	4.58	gi|18379349	synaptic vesicle membrane protein VAT-1 homolog [Homo sapiens]	2						
2	10.78	gi|46276863	parathymosin [Homo sapiens]	1	0.75017	1.47755	1.608629	1.14413	1.249661	1.06365
2	20.2	gi|14211889	protein dpy-30 homolog [Homo sapiens]	1	1.06193	0.74713	0.785391	1.0061	0.706888	0.70462
2	8.791	gi|7657176	protein canopy homolog 2 isoform 1 precursor [Homo sapiens]	1						
2	14.53	gi|7657609	signal peptidase complex catalytic subunit SEC11A [Homo sapiens]	1						

2	4.911	gi|4503727	peptidyl-prolyl cis-trans isomerase FKBP3 [Homo sapiens]	1	0.6618	0.88653	0.711606	0.64077	0.791742	0.88282
2	5.425	gi|5032069	splicing factor 3B subunit 4 [Homo sapiens]	1						
2	4.032	gi|221316575	lymphocyte function-associated antigen 3 isoform 2 [Homo sapiens]	1	1.10848	0.95693	1.071785	0.83056	0.940833	0.95787
2	8.475	gi|341915584	PREDICTED: prothymosin alpha-like [Homo sapiens]	1	1.05324	1.3211	1.186233	1.03384	1.033843	1.26186
1.95	2.64	gi|259155315	mitochondrial 2-oxoglutarate/malate carrier protein isoform 2 [Homo sapiens]	1	1.82798	2.05946	1.756294	1.94721	1.415	1.33975
1.93	11.86	gi|5729850	guanine nucleotide-binding protein G(k) subunit alpha [Homo sapiens]	3	0.56981	0.66885	0.601442	0.40964	0.354968	0.60163
1.92	6.077	gi|4502227	ADP-ribosylation factor-like protein 1 [Homo sapiens]	1						
1.85	7.519	gi|4503659	ubiquitin-like protein fubi and ribosomal protein S30 precursor [Homo sapiens]	1	1.93251	0.52726	0.873157	1.16378	0.939255	1.03131
1.83	1.74	gi|52485606	formin-like protein 2 [Homo sapiens]	2						
1.8	3.93	gi|4505585	platelet-activating factor acetylhydrolase IB subunit beta isoform a [Homo sapiens]	1						

1.8	2.545	gi|68508955	CD97 antigen isoform 3 preproprotein [Homo sapiens]	1						
1.79	3.438	RRRRgi|45021	REVERSED annexin A5 [Homo sapiens]	1						
1.77	10.26	gi|73912720	mitochondrial inner membrane organizing system protein 1 isoform a [Homo sapiens]	1	1.21962	0.9844	0.784586	0.64946	1.253601	1.76032
1.76	1.531	gi|157388967	coiled-coil domain-containing protein 87 [Homo sapiens]	2	0.85902	0.99372	1.045406	0.93941	1.111446	0.61127
1.7	35.73	gi|14141161	heterogeneous nuclear ribonucleoprotein U isoform b [Homo sapiens]	34	1.76342	1.10382	1.33401	1.0886	1.327369	1.53358
1.7	11.64	gi|334688844	glial fibrillary acidic protein isoform 3 [Homo sapiens]	7	0.91713	0.81444	1.222358	1.12989	1.145062	1.32143
1.7	5.932	gi|6005890	transcription elongation factor B polypeptide 2 isoform a [Homo sapiens]	1						
1.7	11.64	gi|334688844	glial fibrillary acidic protein isoform 3 [Homo sapiens]	7	0.91713	0.81444	1.222358	1.12989	1.145062	1.32143
1.69	19.01	gi|4506587	replication protein A 14 kDa subunit [Homo sapiens]	1						
1.68	4.93	gi|50659074	ATP synthase lipid-binding protein, mitochondrial isoform A precursor [Homo sapiens]	1	0.92016	0.90905	0.935273	1.02621	1.145511	0.92555

1.66	11.2	gi|7705477	tRNA methyltransferase 112 homolog [Homo sapiens]	1						
1.64	1.229	gi|122939157	gem-associated protein 4 [Homo sapiens]	2						
1.62	4.93	gi|4504347	hemoglobin subunit alpha [Homo sapiens]	1	1.59508	0.66166	1.879694	0.99311	1.061659	0.51581
1.62	5.797	gi|6678271	TAR DNA-binding protein 43 [Homo sapiens]	2						
1.6	1.143	gi|116256464	uncharacterized protein C2orf55 [Homo sapiens]	2						
1.6	2.105	gi|28178838	isocitrate dehydrogenase [NAD] subunit gamma, mitochondrial isoform b precursor [Homo sapiens]	1						
1.6	2.256	RRgi|166235	REVERSED DNA damage-regulated autophagy modulator protein 2 [Homo sapiens]	1						
1.57	0.5336	gi|297207099	AT-rich interactive domain-containing protein 1B isoform 2 [Homo sapiens]	1						
1.55	3.918	gi|311893345	copine-1 isoform c [Homo sapiens]	1						
1.54	0.503	gi|61676188	E3 ubiquitin-protein ligase HUWE1 [Homo sapiens]	1	1.09776	1.27215	0.877703	1.14276	0.851826	0.89419

1.54	7.469	gi|4506193	proteasome subunit beta type-1 [Homo sapiens]	1	1.43887	2.04849	1.395369	1.22527	1.514252	1.1148
1.51	3.981	gi|4557237	acetyl-CoA acetyltransferase, mitochondrial precursor [Homo sapiens]	1	1.24819	1.1585	1.084654	0.95572	0.973047	1.16623
1.51	3.233	gi|68799814	AP-2 complex subunit mu isoform b [Homo sapiens]	1						
1.5	0.8475	gi|30578410	dolichyl-diphosphooligosaccharide--protein glycosyltransferase subunit STT3B [Homo sapiens]	1						
1.5	0.8706	gi|39780588	pre-rRNA-processing protein TSR1 homolog [Homo sapiens]	1	0.90888	1.05537	0.932494	0.94253	1.05303	1.22672
1.5	1.675	gi|333360851	serpin H1 precursor [Homo sapiens]	1	0.72935	0.7743	0.915738	0.72061	0.754026	0.75084
1.49	4.762	gi|70906441	deoxyuridine 5'-triphosphate nucleotidohydrolase, mitochondrial isoform 1 precursor [Homo sapiens]	1	2.0039	0.45709	1.823647	0.98667	1.09756	1.21117
1.48	4.02	gi|312596881	26S protease regulatory subunit 8 isoform 2 [Homo sapiens]	1						
1.47	1.849	gi|18426915	drebrin isoform a [Homo sapiens]	1						

1.47	1.98	gi|4503915	trifunctional purine biosynthetic protein adenosine-3 isoform 1 [Homo sapiens]	1	1.39568	1.34603	0.916473	0.99879	1.676535	1.02895
1.47	1.44	gi|296841091	protein kinase C and casein kinase substrate in neurons protein 2 isoform A [Homo sapiens]	1						
1.47	1.626	gi|139394599	hypermethylated in cancer 2 protein [Homo sapiens]	1	1.13664	1.05848	0.96557	1.0482	1.011859	0.96627
1.46	1.559	gi|8051636	exportin-T [Homo sapiens]	1						
1.45	1.241	gi|359279861	dihydropyrimidinase-related protein 5 [Homo sapiens]	1						
1.44	3.081	gi|4503519	eukaryotic translation initiation factor 3 subunit F [Homo sapiens]	1						
1.38	7.634	gi|4757926	RNA-binding protein 39 isoform b [Homo sapiens]	2						
1.36	4.233	gi|115430112	receptor expression-enhancing protein 5 [Homo sapiens]	1	0.81179	1.07698	1.398986	0.48126	1.126422	1.05482
1.31	59.91	gi|14389309	tubulin alpha-1C chain [Homo sapiens]	53						
1.31	3.704	gi|52426743	luc7-like protein 3 [Homo sapiens]	1						

1.3	3.965	gi|8923390	coiled-coil-helix-coiled-coil-helix domain-containing protein 3, mitochondrial precursor [Homo sapiens]	1	0.85987	0.85484	0.750112	1.52743	0.919463	0.55202
