# Supplementary material for: Upregulation of Glycolytic Enzymes, Mitochondrial Dysfunction and Increased Cytotoxicity in Glial Cells Treated with Alzheimer’s Disease Plasma
Source: PLoS One. 2015 Mar 18;10(3):e0116092. doi: 10.1371/journal.pone.0116092 (PMC4364672; doi:10.1371/journal.pone.0116092)
Supplement: S3 Table — FDR analysis summary at the protein, peptide and spectral levels for iTRAQ experiment from Protein Pilot v4. (PDF) [file pone.0116092.s005.pdf]

Table S3
Protein, Peptide and Spectral level False Discovery Rates Analysis results from Protein Pilot v4.0

Single Table Summary of All Results

	Data Level	FDR Type	FDR	ID Yield
Identification Yield at FDR Threshold	Protein	Local	1%	721
			5%	750
			10%	773
		Global	1%	803
			5%	882
			10%	951
	Distinct peptide	Local	1%	5033
			5%	6325
			10%	6869
		Global	1%	6733
			5%	8297
			10%	9351
	Spectral	Local	1%	26526
			5%	33626
			10%	36751
		Global	1%	35876
			5%	44801
			10%	50901
Corresponding Confidence in ProteinPilot™ Software	Protein	Local	1%	99.0%
			5%	97.7%
			10%	96.6%
		Global	1%	93.4%
			5%	81.4%
			10%	69.1%
	Distinct peptide	Local	1%	99.1%
			5%	93.9%
			10%	86.3%
		Global	1%	88.8%
			5%	53.8%
			10%	38.2%
	Spectral	Local	1%	97.4%
			5%	79.1%
			10%	62.0%
		Global	1%	67.0%
			5%	27.9%
			10%	16.1%
